# Uncovering the molecular identity of cardiosphere-derived cells (CDCs) by single-cell RNA sequencing

**DOI:** 10.1007/s00395-022-00913-y

**Published:** 2022-03-08

**Authors:** Palgit-S. Kogan, Felix Wirth, Archana Tomar, Jonatan Darr, Raffaele Teperino, Harald Lahm, Martina Dreßen, Nazan Puluca, Zhong Zhang, Irina Neb, Nicole Beck, Tatjana Luzius, Luis de la Osa de la Rosa, Kathrin Gärtner, Corinna Hüls, Reinhard Zeidler, Deepak Ramanujam, Stefan Engelhardt, Catharina Wenk, Lesca M. Holdt, Mimmi Mononen, Makoto Sahara, Julie Cleuziou, Jürgen Hörer, Rüdiger Lange, Markus Krane, Stefanie A. Doppler

**Affiliations:** 1 School of Medicine and Health, Department of Cardiovascular Surgery, Institute Insure, Technical University of Munich, German Heart Center Munich, Lazarettstrasse 36, 80636 Munich, Germany; 2 Institute of Experimental Genetics, Helmholtz Zentrum München, German Research Center for Environmental Health, Neuherberg, Germany; 3 German Center for Diabetes Research (DZD), Neuherberg, Germany; 4 Research Unit Gene Vectors, Helmholtz Center Munich German Research Center for Environmental Health, Munich, Germany; 5 Department of Otorhinolaryngology, Klinikum der Universität (KUM), Munich, Germany; 6 DZHK (German Center for Cardiovascular Research)-Partner Site Munich Heart Alliance, Biedersteiner Straße 29, 80802 Munich, Germany; 7 Institute of Pharmacology and Toxicology, Technische Universität München, Biedersteiner Str. 29, 80802 Munich, Germany; 8 Institute of Laboratory Medicine, University Hospital, Ludwig Maximilians University Munich, Munich, Germany; 9 Department of Cell and Molecular Biology, Karolinska Institutet, 171 77 Stockholm, Sweden; 10 Department of Surgery, Yale University School of Medicine, CN06510 New Haven, CT USA; 11 School of Medicine and Health, Department of Pediatric and Congenital Heart Surgery, Institute Insure, Technical University of Munich, Lazarettstraße 36, 80636 Munich, Germany; 12 School of Medicine and Health, Department of Pediatric and Congenital Heart Surgery, Technical University of Munich, German Heart Center Munich, Lazarettstraße 36, 80636 Munich, Germany; 13 Division of Congenital and Pediatric Heart Surgery, University Hospital of Munich, Ludwig-Maximilians-Universität, Munich, Germany; 14 Division of Cardiac Surgery, Department of Surgery, Yale University School of Medicine, New Haven, CT USA

**Keywords:** Cardiosphere-derived cells (CDCs), Cardiac non-myocyte cells, Cardiac fibroblasts, Right atrial biopsy, Single-cell RNA sequencing, Extracellular vesicles

## Abstract

Cardiosphere-derived cells (CDCs) generated from human cardiac biopsies have been shown to have disease-modifying bioactivity in clinical trials. Paradoxically, CDCs’ cellular origin in the heart remains elusive. We studied the molecular identity of CDCs using single-cell RNA sequencing (sc-RNAseq) in comparison to cardiac non-myocyte and non-hematopoietic cells (cardiac fibroblasts/CFs, smooth muscle cells/SMCs and endothelial cells/ECs). We identified CDCs as a distinct and mitochondria-rich cell type that shared biological similarities with non-myocyte cells but not with cardiac progenitor cells derived from human-induced pluripotent stem cells. *CXCL6* emerged as a new specific marker for CDCs. By analysis of sc-RNAseq data from human right atrial biopsies in comparison with CDCs we uncovered transcriptomic similarities between CDCs and CFs. By direct comparison of infant and adult CDC sc-RNAseq data, infant CDCs revealed GO-terms associated with cardiac development. To analyze the beneficial effects of CDCs (pro-angiogenic, anti-fibrotic, anti-apoptotic), we performed functional in vitro assays with CDC-derived extracellular vesicles (EVs). CDC EVs augmented in vitro angiogenesis and did not stimulate scarring. They also reduced the expression of pro-apoptotic *Bax* in NRCMs. In conclusion, CDCs were disclosed as mitochondria-rich cells with unique properties but also with similarities to right atrial CFs. CDCs displayed highly proliferative, secretory and immunomodulatory properties, characteristics that can also be found in activated or inflammatory cell types. By special culture conditions, CDCs earn some bioactivities, including angiogenic potential, which might modify disease in certain disorders.

**Supplementary Information:**

The online version contains supplementary material available at 10.1007/s00395-022-00913-y.

## Introduction

Cardiosphere-derived cells (CDCs) are cells of intrinsic cardiac origin [67] attributed with anti-fibrotic, anti-inflammatory, and pro-angiogenic properties. CDCs are generated from human heart biopsies by well-established protocols sometimes including growth factor treatment. These cells have been used in several clinical trials showing evidence of disease-modifying bioactivity [11, 29, 30, 40, 41, 51, 54, 60]. The CADUCEUS trial, treating adult patients post-myocardial infarction with left ventricular dysfunction by autologous CDCs, did not observe significant functional improvement [41]. Pediatric patients with single-ventricle (SV) physiology who received autologous CDC-transplantation showed beneficial changes in ventricular function compared to controls (phase I TICAP trial) [30]. The phase II trial (PERSEUS) revealed that CDC treatment is associated with improved ventricular volumes, somatic growth, increased trophic factor production and quality of life in SV patients which was confirmed by a 2-year follow-up [29, 51].

Despite this widespread clinical use, molecular cell characteristics of CDCs have not been analyzed in detail and their cellular origin in the heart is still not elucidated. Previously described characteristics of CDCs are the absence of the hematological marker CD45 and the ubiquitous expression of the mesenchymal marker CD105 [41]. Heterogeneity of CDCs was shown by inconsistent expression of the fibroblast marker THY1 (CD90) ranging from 25% [41] to over 60% [30] and deviations concerning the expression of the endothelial marker CD31 or c-KIT [55]. However, it has been shown that c-KIT is not relevant for the therapeutic effects of CDCs since the active fraction is composed of CD105 + /CD90-/c-KIT-cells [12]. Anyway, the presence of c-KIT as an indicator for cardiac stem or progenitor cells has been sharply criticized [13]. Nowadays, the accepted paradigm is that CDCs have paracrine effects, partly mediated by macrophages [17], rather than being a type of heart resident progenitor cell [14]. Increasing evidence indicated that these paracrine effects are mediated by extracellular vesicles (EVs) secreted by CDCs [23, 28, 64].

In this manuscript, we sought to elucidate the molecular identity of CDCs and their cellular origin in the adult heart. We compared this artificially generated cell type to the major groups of non-myocyte and non-hematopoietic cells of the human heart: cardiac fibroblasts (CFs), endothelial cells (ECs) and smooth muscle cells (SMCs) by single-cell RNA sequencing (sc-RNAseq). In addition, we analyzed human right atrial tissue by sc- as well as single-nucleus (sn) RNAseq to gain information about the cellular origin of the CDC population. To analyze CDCs’ paracrine function, we further applied EVs to different cardiac cell types and investigated angiogenesis, fibrosis and cardiomyocyte apoptosis. Additionally, we compared infant- and adult-derived CDCs concerning their molecular signature and paracrine effects.

## Materials and methods

Please find the complete materials and methods section in the supplemental material online.

### Patient-derived samples

Human primary cells (CDCs, AFs, CFs, SMCs, ECs) were generated from biopsies derived from adipose, atrial or vessel tissue from patients undergoing heart surgery (age: 5 days to 76 years) (Suppl Table 1 and 2). Human right atrial appendage tissue for single nuclei (sn) and single cell (sc) RNA sequencing (RNAseq) was also obtained from patients undergoing heart surgery (Suppl Table 1, Suppl Table 9). All patients had signed an informed consent. In the case of infants, their parents or legal guardians signed the informed consents. The local ethics committee of the Technical University of Munich Medical School supervised and approved the study (project number 570/16S). Tissue sampling within the framework of the cardiovascular biobank at the German Heart Center Munich was also approved by the local ethics committee of the Medical School of the Technical University of Munich (project number 5943/13). All experimental procedures were performed in accordance with the principles outlined in the Declaration of Helsinki.

### Animals

Murine cardiac fibroblasts (CFs) were generated from adult hearts of transgenic Nkx2.5 cardiac enhancer eGFP mice [68]. The mice were anesthetized with isoflurane and then euthanized by cervical dislocation to extract their hearts. Neonatal rat cardiomyocytes (NRCMs) were isolated from 0–1 day old Sprague Dawley rats after decapitation.

Mice and rats were housed in accredited facilities in compliance with the European Community Directive related to laboratory animal protection (2010/63/EU). All animals sacrificed for harvesting organs were approved by the relevant authority “Regierung von Oberbayern” [Regional Government of Upper Bavaria], German TierSchG (Animal protection law). All animal experiments (organ extractions) were performed in accordance with the European guidelines and regulations for animal care and handling (Directive 2010/63/EU).

## Results

### Characterization of adult cardiosphere-derived cells (CDCs) compared to cardiac non-myocyte cell types

**Fig. 1:**
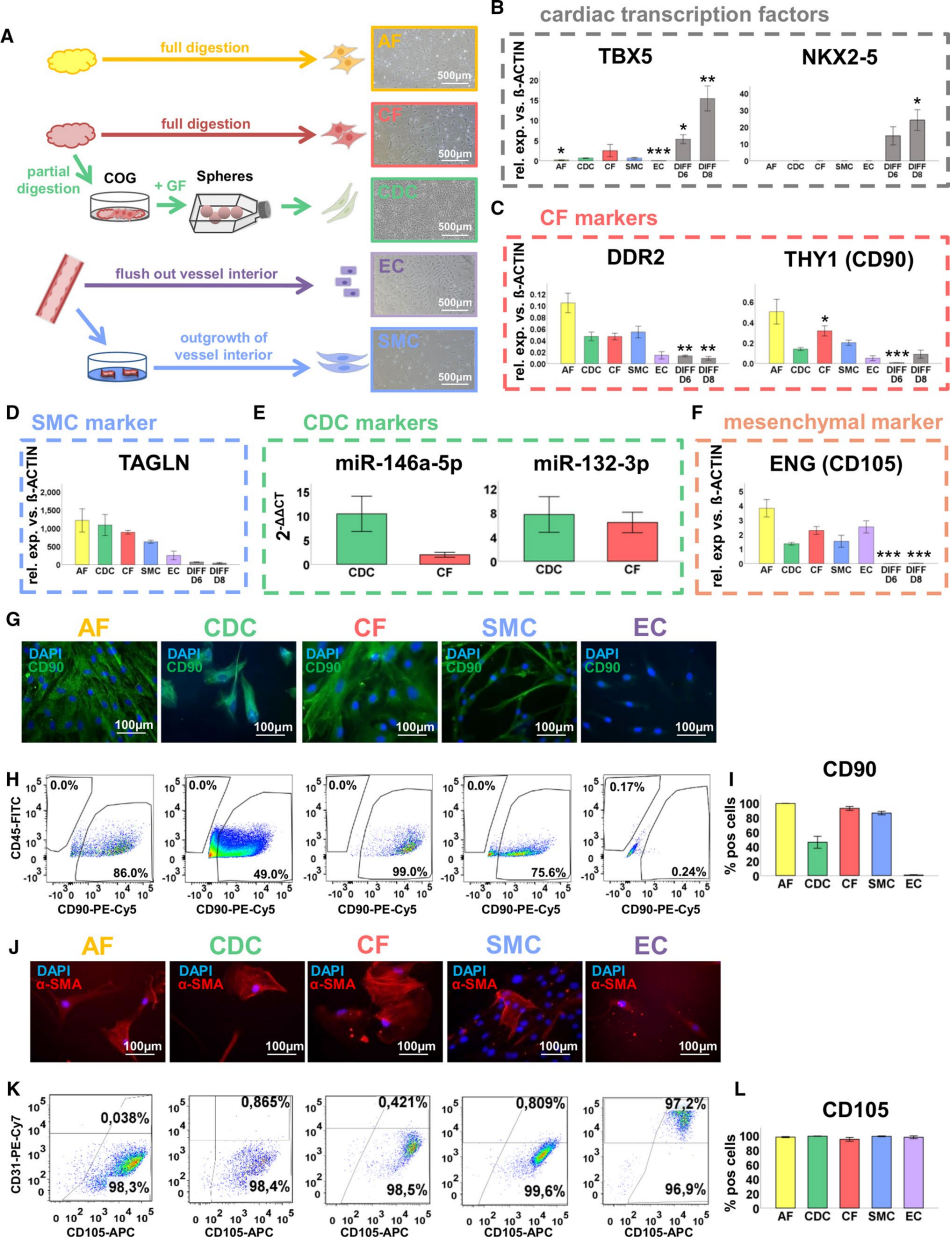
Characterization of adult CDCs compared to other primary non-myocyte cell types. **A** Generation of adipose tissue-derived fibroblasts (AF), cardiac fibroblasts (CF), cardiosphere-derived cells (CDC), endothelial cells (EC) and smooth muscle cells (SMC). Abbreviations: COG, cardiac outgrowth; GF, growth factors. **B–F** Gene expression analysis of CDCs compared to primary cells and human-induced pluripotent stem cell-derived cardiac progenitor cells from day 6 (DIFF D6) and immature cardiomyocytes from day 8 of cardiac differentiation (DIFF D8) (only significant differences against CDCs are depicted). Relative RNA expression versus *β-ACTIN* is illustrated for **B** cardiac transcription factors *TBX5* and *NKX2-5*, **C** CF markers *DDR2* and *THY1* (*CD90*), **D** SMC marker *TAGLN*, **E** CDC-typical microRNAs miR-146a-5p and miR-132-3p, and **F** mesenchymal marker *ENG* (*CD105*). **G** Immunocytochemical (ICC) staining against CD90 showed ubiquitous CD90 expression in AFs, CDCs, CFs and SMCs but not in ECs. **H–I** Flow cytometry analysis with CD90 antibodies (conjugated with PE-Cy5) confirmed ICC results but only 40–60% of CDCs expressed CD90. **H** Exemplary dot plots and **I** percentage of CD90-positive cells. **J** ICC staining against SMC marker α-smooth muscle actin (α-SMA) revealed ubiquitous expression in AFs, CDCs, CFs and SMCs and to a lower extent also in ECs. **K-L** Flow cytometry analysis with CD105 antibodies (conjugated with APC) depicted ubiquitous CD105 expression in AFs, CDCs, CFs, SMCs and ECs. **K** Exemplary dot plots and **L** percentage of CD105-positive cells. Data are represented as means ± SE, *p < 0.05, **p < 0.01, ***p < 0.001 (only significances against CDCs are depicted). A complete overview of *p*-values in Suppl. Table 3 (qRT-PCR) and Suppl. Table 4 (Flow cytometry). Parts of the figure were created with Biorender.com

Cardiosphere-derived cells (CDCs) were generated from adult right atrial appendage tissue from patients undergoing cardiac surgery (Suppl Table 1, mean age: 63.92 years ± 8.23 years). CDCs were established by the production of “3D-cardiospheres” including stimulation with growth factors (Fig. 1A) [30, 43]. To assess the CDCs’ distinct molecular profile, they were compared to cardiac fibroblasts (CFs), smooth muscle cells (SMCs), endothelial cells (ECs), and adipose tissue-derived fibroblasts (AFs) as a non-cardiac cell type. CFs and AFs were isolated from cardiac or subcutaneous adipose tissue, respectively (Fig. 1A; Suppl Table 1). ECs and SMCs were generated from thoracic vessels of patients undergoing coronary artery bypass graft (CABG) surgery (Fig. 1A; Suppl Table 1). Cardiac progenitor cells derived from differentiated human induced pluripotent stem cells (hiPSCs [57]) on day six (DIFF D6) and immature cardiomyocytes on day eight (DIFF D8) were further used as controls for the expression of cardiac transcription factors [7] (Suppl Fig. S1A).

First, gene expression levels of several markers were assessed by qRT-PCR (Fig. 1B–F; Suppl Fig. S1B–E). Cardiac transcription factors such as *TBX5*, *NKX2-5* and *GATA4* have been described to be highly expressed in CDCs compared to fibroblasts [30]. Here, *GATA4*, *TBX5* and *NKX2-5* [22, 24, 27] were significantly upregulated in DIFF D6 and DIFF D8 (only *TBX5* and *NKX2-5*) compared to CDCs. No significant differences were detected between CDCs and CFs (Fig. 1B, Suppl Fig. S1B). *GATA4* was albeit significantly lower expressed in AFs, SMCs and ECs compared to CDCs. CF markers *DDR2* and *PDGFRA* [19] or cardiac fibrosis associated microRNA (miR)-21 [49, 62] were not significantly higher expressed in CFs compared to CDCs, whereas CF markers *ALDH1A2* [18] and *THY1* (CD90) [19] were significantly increased in CFs compared to CDCs (Suppl Fig. S1C, Fig. 1C). Well-known SMC markers *TAGLN* [3] and *PDGFRB* [42] were also expressed in fibroblasts and CDCs (Fig. 1D; Suppl Fig. S1D). ECs were clearly distinguishable from all other cell types by high expression of typical EC markers *PECAM1* (CD31) and *CDH5* (Suppl Fig. S1E) [18]. Interestingly, CDCs exhibited significantly higher *CDH5* expression than AFs, CFs and SMCs. MiR-146a, described as a marker for CDC-derived exosomes and as partially responsible for their beneficial effects [28, 64], was threefold higher expressed in CDCs compared to CFs but did not reach significance (Fig. 1E). MiR-132, enriched in EVs derived from cardiac outgrowth cells [4], was likewise not upregulated in CDCs versus CFs (Fig. 1E). The mesenchymal marker *ENG* (CD105), a quality marker for CDCs [41], was equally expressed in all non-myocyte cells and CDCs but was downregulated in DIFF D6 and DIFF D8 (Fig. 1F).

Immunocytochemical stainings or flow cytometry evaluated protein levels of selected markers. Whereas DDR2 was ubiquitously abundant in all analyzed cell types (Suppl Fig. S1F), flow cytometry revealed that only 40–60% of the CDCs expressed CD90 (Fig. 1G–I). Smooth-muscle actin alpha (α-SMA, *ACTA2*), a SMC marker [69], was expressed to a comparable level in fibroblasts and CDCs (Fig. 1J). High abundance of CD31 (*PECAM1*) in ECs was confirmed by immunocytochemistry and flow cytometry (Suppl Fig. S1G–I). CD105 was found to be expressed ubiquitously in all analyzed cell types (Fig. 1K, L). Flow cytometry (Suppl Fig. S1J) confirmed the absence of the hematopoietic marker CD45 in CDCs [41] and for all non-myocyte cell types.

To conclude, CDCs showed similar molecular characteristics like non-myocyte cell types. However, CDCs clearly differed from veritable cardiac progenitor cells (DIFF D6) and immature cardiomyocytes (DIFF D8). Well-described CDC markers, such as CD105, were equally expressed in AFs, CFs, SMCs, and ECs.

### Single-cell RNA sequencing of CDCs compared to the main cardiac non-myocyte cell types

We next assessed the differences between CDCs and the three main cardiac non-myocyte cell types CFs, SMCs and ECs in more detail using sc-RNAseq. All cells were generated from discarded tissue during CABG surgery as mentioned above (Suppl Table 5, age: 61–66 years; Fig. 1A). As a quality control, we carefully checked cell morphology (Suppl Fig. S2A) and some of the above-mentioned specific markers (Suppl Fig. S2B, C). Single-cell transcriptional profiling was performed using the 10 × chromium platform, followed by bioinformatical analyses with the Seurat software suite. To assess transcriptional differences between cell types single-cell data were integrated and canonical correlation analysis (CCA) was performed [8] to remove potential batch effects. To remove low-quality cells and doublets, cells with very high mitochondrial gene percentage were filtered out [38] (Suppl Fig. S2D–F, Suppl Table 6). Finally, 2815 cells were analyzed with a median of 3428 genes per cell and 17,078 UMI counts per cell (Suppl Fig. S2G, H, Suppl Table 7).

CDCs exhibited a higher median percentage of mitochondrial genes (6%) compared to CFs (1.3%), SMCs (2.5%) and ECs (3.0%) (Suppl Fig. S2F, Suppl Table 7) pointing to a mitochondria-rich cell type with high energy needs.

**Fig. 2:**
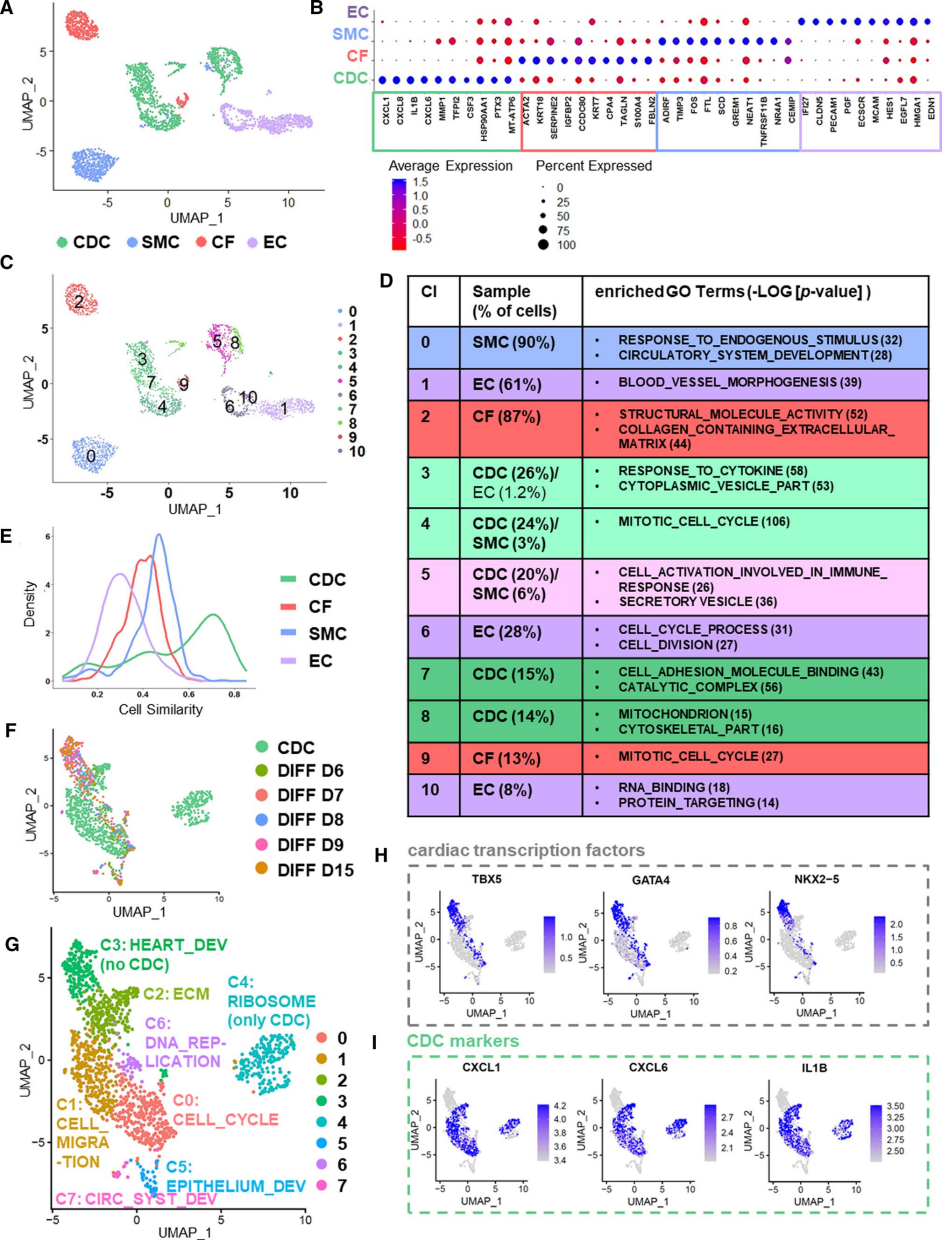
Single-cell RNA sequencing (sc-RNAseq) of CDCs compared to the main cardiac non-myocyte cell types (CFs, SMCs, ECs) and differentiating hESCs. **A** UMAP plot of analyzed cell samples from sc-RNAseq colored by sample identifier. **B** Top ten upregulated differential expressed genes (uDEGs) for each cell type sorted by the average log fold change (avg_logFC) compared to all other cell samples (filtering parameters *p* < 0.05, avg_logFC ≥ 0.25). **C** UMAP plot of analyzed cell samples in sc-RNAseq colored by cluster. Eleven clusters were identified. **D** GO (gene ontology) terms significantly enriched for each cluster (Cl) analyzed by gene set enrichment analysis (GSEA). Column two reports the amount of cells for each sample included per cluster (percentages < 1% are not displayed). **E** Transcriptional similarity plots of CDCs, CFs, SMCs and ECs generated from sc-RNAseq data. **F** UMAP plot of sc-RNAseq-CDCs integrated with sc-RNAseq data of differentiating hESCs [44] at various stages (DIFF D6—DIFF D15) colored by sample identifier. **G** UMAP plot of sc-RNAseq-CDCs integrated with sc-RNAseq data of differentiating hESCs [44] at various stages (DIFF D6—DIFF D15) colored by clusters (unsupervised clustering). **H** UMAP plots generated from sc-RNAseq data showing expression levels of cardiac transcription factors *TBX5, GATA4 and NKX2-5.* **I** UMAP plots generated from sc-RNAseq data showing expression levels of CDC markers (**B**) *CXCL1, CXCL6 and IL1B*

Subsequently, we performed unsupervised clustering using Seurat [8, 39, 52]. Figure 2A depicts the cell types according to their cell identity. For CDCs, CFs, SMCs and ECs, the top ten of upregulated genes were identified (Fig. 2B). Among those cell types, CDCs exclusively expressed genes encoding for chemokine ligands (such as *CXCL1/8/6*) or cytokines (*IL1B* and *CSF3*). *CXCL6/8* as well as *IL1B* expression was specific for CDCs compared to the other non-myocyte cell types (Suppl Fig. S2I, S3A). Only *CXCL1* was also expressed in a few ECs (Suppl Fig. S2I). *FBLN2, S100A4, ACTA2* and *TAGLN* were not specific for CFs, but also expressed in SMCs and partly in CDCs (not *FBLN2*) (Fig. 2B, Suppl Fig. S3B, C). Many genes highly expressed in SMCs were also expressed in CFs and CDCs (Suppl Fig. S3D). The most upregulated genes in ECs *IFI27*, *CLDN5,* and *PECAM1* (*CD31*) were specific for ECs (Fig. 2B; Suppl Fig. S3E, F). Looking at these 40 markers in Fig. 2B CDCs express 17 markers in common with CFs (42.5%), 18 markers in common with SMCs (45%) but only 10 markers in common with ECs (25%).

Sc-RNAseq expression profiles of markers already analyzed by qRT-PCR (Fig. 1; Suppl Fig. S1) corresponded to previous results (Suppl Fig. S4A, B). The reliability of sc-RNAseq data was confirmed by validating selected gene expression by qRT-PCR in independent samples (Suppl Fig. S4C–E).

Unsupervised global subclustering of CFs, SMCs, ECs, and CDCs according to their specific gene expression subdivided them into eleven distinct clusters (Fig. 2C). Visually, all cell types split up into two main clusters sometimes with further sub-clusters, especially for CDCs, but also ECs (CDCs: Cl 3, 4, 5, 7, 8; CFs: Cl 2, 9; ECs: Cl 1, 6, 10; SMCs: Cl 0, 4, 5) (Fig. 2C).

To analyze the biological features of the clustered cells, we performed gene set enrichment analysis (GSEA) [56] of the selectively upregulated differentially expressed genes (uDEGs) in each cluster (Suppl Table S8). The main clusters for SMCs (Cl 0, 90% of SMC), ECs (Cl 1, 61% of EC) and CFs (Cl 2, 87% of CF) revealed biological properties such as angiogenesis (Cl0, Cl1), extracellular matrix organization (Cl0, Cl2), locomotion (Cl0,Cl1), or adhesion (Cl0, Cl2) and by this confirmed the identity of selected cell samples for sc-RNAseq (Suppl Fig. S4F, Suppl Fig. S5A, B, Suppl Table S8).

To better understand CDCs, we went into GSEA of all five CDC clusters. All clusters exhibited main immunomodulatory properties such as “response to cytokine” (Fig. 2D, Suppl Fig. S5C). The major difference between the two main clusters Cl4/7/3 and Cl5/8 was that the CDCs in Cl5/8 seemed to be involved in more energetic processes (“mitochondrion”, “cellular respiration”, Fig. 2D, Suppl Table S8), corresponding to the fact that CDCs exhibited a higher median percentage of mitochondrial genes than CFs, ECs and SMCs as noticed above (Fig. S2F). Energy-consuming processes for Cl5-CDCs (20% of CDCs) might correspond to “cell activation” while the energy in Cl8-CDCs (14% of CDCs) seemed to be necessary for cytoskeleton organization (Fig. 2D, Suppl Table S8). Cl3, Cl5 and Cl7 were enriched for processes associated with the immune system and “secretion” (Fig. 2D, Suppl Table S8). To elucidate which molecules might be secreted by CDCs we analyzed upregulated genes in Cl3, Cl5 and Cl7 in comparison to the other clusters in Fig. 2C. Several genes in this list encode for proteins known to be secreted by CDCs, for example *VEGFA*, *VEGFB* and *FGF2* [37]. Interestingly, we observed many genes crucial for angiogenesis, such as *TAGLN2*, *ICAM1*, *FGF2*, *VEGFA*, and *VEGFB*. Besides, several chemokines (*CXCL6*, *CXCL1*, *CXCL8*, *CXCL3*, *CXCL5*, *CXCL2*), interleukins (*IL1B*, *IL1A*, *IL24*, *IL6*, *IL32*, *IL6ST*, *IL11*, *IL1R1*) and *TGFB1/TGFB2* were found, suggesting that CDCs secrete immunomodulatory molecules.

Cl4-CDCs (24% of CDCs) showed “negative regulation of the cell-cycle” (Fig. 2D, Suppl Table S8) and thus might be quiescent cells. Cl7-CDCs (15% of CDCs) included cells that were still proliferative but also started catabolic processes (Fig. 2D, Suppl Table S8). Cl3-CDCs (26% of CDCs) were activated cells, able to migrate (“cell locomotion”), and involved in cell signaling as well as in developmental processes (Fig. 2D, Suppl Table S8).

To better understand whether significant similarities between CDCs and CFs, ECs or SMCs persist, cell similarity was calculated [65]. Three peaks appeared in the CDC sample (Fig. 2E): Whereas the highest peak stood out, the second-highest peak had approximately the same similarity score as CFs and SMCs, and the smallest peak paralleled the small peak of the SMC curve. Similarly upregulated genes in CDCs, CFs and SMCs were e.g. associated with GO terms such as “extracellular matrix” and “biological adhesion” (Suppl Fig. S6A). Associated exemplary genes *COL6A2* and *COL3A1* were expressed in CDCs, CFs and SMCs, but barely in ECs (Suppl Fig. S6B, C). qRT-PCR confirmed sc-RNAseq expression patterns in independent samples (Suppl Fig. S6D, E).

So far, sc-RNAseq results showed that CDCs exhibited the highest amount of mitochondrial genes and were distinguished from CFs, SMCs and ECs by secretory and immunomodulatory characteristics. However, CDCs also showed certain similarities to non-myocyte cell types.

### Single cell RNA sequencing of CDCs compared to differentiating human ESCs

To compare CDCs with veritable cardiac progenitor cells (CPCs) and early cardiomyocytes (CMs) in more detail we included single-cell transcriptome data of differentiating human ESCs (hESCs) generated for a trajectory mapping of lineage decisions during hESC differentiation [44]. A similar “cardiac” differentiation protocol was used for the hESCs [44] like for hiPSCs described in Fig. 1 and Suppl Fig. S1. Sc-RNAseq data of CDCs and hESC DIFF D6/D7/D8/D9/D15 were integrated and UMAP plots were generated (Fig. 2F, G). Eight clusters emerged by unsupervised clustering (Fig. 2G). As we assumed before (Fig. 1, Suppl Fig. S1) CDCs did not overlap with CPCs or early CMs clearly marked by *TBX5*, *GATA4* and *NKX2-5* (cluster 3 associated with heart development) (Fig. 2F–H, Suppl Fig. S6F). In addition, there is no overlap of CDCs with epithelial (cluster 5) or angiogeneic progenitor cells (cluster 7) (Fig. 2F, G, Suppl Fig. S6F–G). Interestingly, these progenitor cells expressed *c-KIT* (Suppl Fig. S6G), which was completely absent in CDCs. However, CDCs overlap with differentiating hESCs in clusters, that show CF- (cluster 2) or proliferating cell (cluster 0, 6) characteristics (Fig. 2F, G, Suppl Fig. S6F, H). Cluster 1 and cluster 4 mainly consist of CDCs and show enrichment for GO terms such as “cell migration”, “anchoring junction”, “translation elongation” and “ribosome” (Fig. 2F, G, Suppl Fig. S6F). Interestingly, earlier defined markers for CDCs such as *CXCL1*, *CXCL6* and *IL1B* (Fig. 2B) were highly specific for CDCs in this setting (Fig. 2I).

In conclusion, no overlap was found with cardiac progenitor cells or early cardiomyocytes by comparing sc-RNAseq data of CDCs to differentiating hESCs.

### Comparison of CDCs and right atrial human biopsies by sc-RNAseq

**Fig. 3:**
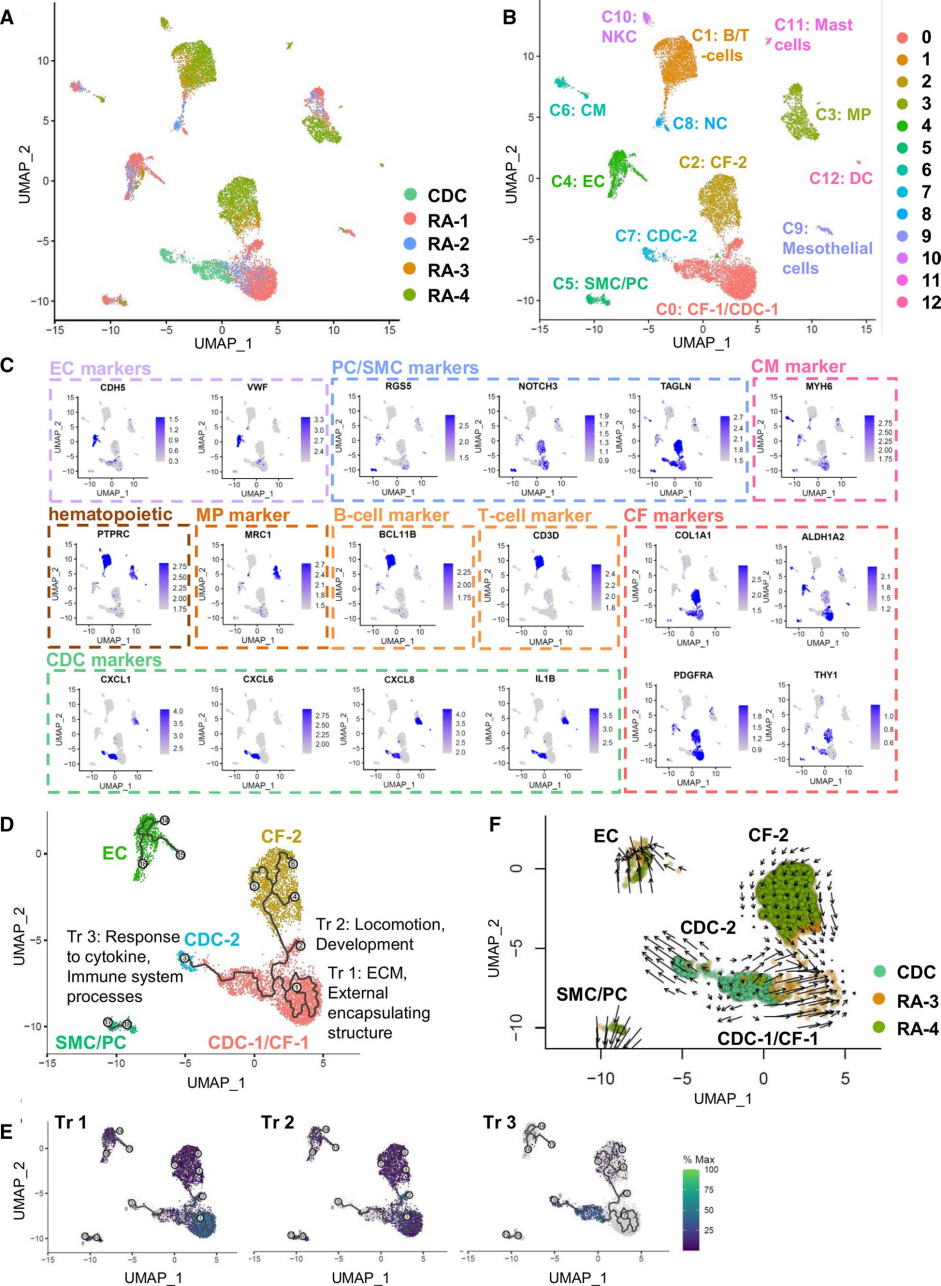
Comparison of CDCs and human atrial biopsies by sc-/sn-RNAseq.** A** UMAP plot of adult CDC sc-RNAseq data integrated with sn/sc-RNAseq data from four human right atrial biopsies. Color indicates sample identifier. **B** UMAP plot of adult CDC sc-RNAseq data integrated with sn/sc-RNAseq data from four human right atrial biopsies. Color indicates cluster identity. Unsupervised clustering revealed 13 clusters identifying all main cell types of the human heart including rare cell populations (see also Suppl Fig. S7C). Abbreviations: CM, cardiomyocytes; DC, dendritic cells; MP, macrophages; NC, neuronal cells; NKC, natural killer cells; PC, pericytes; SC, single cell data; SN, single nuclei data; **C)** UMAP plots showing gene expression levels of various markers defining cell type identity of the clusters (see also Suppl Fig. S7B, C). **D** Zoomed view of trajectories detected in CF and CDC clusters. Color indicates cluster identity. Abbreviations: Tr, Trajectory **E** Overlapping gene expression of 3 top specific genes for each trajectory (Tr1: *COL4A4, LAMA2, RORA*; Tr2: *TBX18, TBX20, NR4A1*; Tr3: *CXCL1, SERPINE1, CXCL6*) **F** RNA velocity analysis performed by Velocyto. Velocity field projected onto the UMAP plot. Zoomed view of EC, SMC/PC, CF and CDC clusters. Color indicates sample identity. Arrows show the local average velocity and point from the CDC-2 cluster to the CDC-1/CF-1 cluster

Next, we sought to elucidate the originating cell population of CDCs in the adult human heart. Therefore, we used right atrial human biopsies from four different individuals and performed either single nuclei (RA-1, RA-2) or single cell (RA-3, RA-4) RNAseq on them (Suppl Table 9). Single nuclei (sn) RNAseq data were generated before [34] and were included to analyze cardiomyocytes (CMs) which cannot be investigated with our protocol for sc-RNAseq due to methodological reasons (size limitation of 10X single cell controller ~ 40 µm). Sn- and sc-RNAseq biopsy data were integrated with adult CDC sc-RNAseq data and UMAP plots were generated. UMAP places related cell types near one another [9]. First of all, single cells and single nuclei clustered nicely together, so that we were able to identify all major cardiac cell types in the four biopsy samples: CMs, CFs, ECs, SMCs/pericytes (PCs), monocytes/macrophages (MPs) and B-/T-cells (Fig. 3A–C, Suppl Fig. S7A). In addition, several small cell populations were identified (Fig. 3B, Suppl Fig. S7B). The new CDC markers *CXCL1/6/8* and *IL1B* (Fig. 2B) were also expressed in macrophages, dendritic cells and mast cells (except *CXCL6*) (Fig. 3C). Interestingly, CDCs were located in immediate proximity to CFs (Fig. 3A, B, Suppl Fig. S7A). 77% of CDCs even share the CF-1 cluster. 23% of CDCs build their own cluster (Cl7: CDC-2). CMs were situated far away from CDCs and ECs and pericytes/SMCs were located at bigger distances to CDCs than the CF populations.

Thus, we supposed that CDCs might be a cell-state transition of CFs. To test this, we sought to use single-cell trajectory analysis. We used monocle, an algorithm that applies reversed graph embedding to describe multiple fate decisions in a fully unsupervised manner [48]. Cells will be arranged along reconstructed “trajectories” of any type of inferred biological transition [48]. These trajectories describe gene expression changes that occur while a cell proceeds through the biological process under study [48]. In the two clusters that contain CDCs (Cl7: CDC and Cl2: CF-1/CDC) we detected three trajectory branches (numbered 1, 2 and 3) (Fig. 3D). CDCs and CFs build three major trajectories where CDCs are at the one end and CFs at the other end of the pseudospace. To understand the trajectory branches we investigated the associated genes. For this, we performed additional subcluster analyses in Seurat and analyzed the Top 25 specific genes in cluster 0 (mapping to trajectory 1), 7 (mapping to trajectory 2) and 12 (mapping trajectory 3) (Suppl Table S10, Suppl Fig. S7D, E, Fig. 3E). GSEA revealed that trajectory 1 was mainly associated with “External encapsulating structure” and ECM organization. Trajectory 2 was related to “locomotion” and “development”. Trajectory 3, the “CDC-trajectory”, was linked to “response to cytokine”, immune system processes and secretion (Fig. 3D, Suppl Fig. S7E).

To further reveal whether CDCs constitute a transcriptional transition state of CFs we finally performed RNA velocity analysis, a derivative corresponding to the gene expression state, to predict the potential directionality of cell state transitions [25, 33]. RNA velocity describes the rate of gene expression change for an individual gene at a given time point based on the ratio of its spliced (mature) and unspliced (native) mRNA [5, 33]. We used Velocyto that is based on transcriptional dynamics and accounts for the direction of motion [5, 33]. RNA velocity analysis is not feasible for sn-RNAseq in comparison with scRNAseq data since nuclei still contain unspliced pre-mRNA molecules including introns. Thus, we had to exclude sn-RNAseq-samples (RA-1, RA-2) and compared the CDCs to the sc-RNAseq data of two adult right atrial biopsies (RA-3, RA-4). Usually, RNA velocity analysis is applied to model developmental processes, such as neurogenesis [33]. However, to prove the assumption that such an algorithm can also be used to analyze the origin of cultivated cells in their originating biopsy we analyzed the cultivated CFs, SMCs and ECs (analyzed in detail in Fig. 2) together with the data of the atrial biopsies (RA-1—RA-4). The clustering and the identification of the major cell types for this analysis are displayed in Suppl Fig. S8A–D. Next, we analyzed single-cell trajectories and found, similar to the CDCs, that cultivated and atrial CFs (CF-3/Cl7: mainly cultivated CFs), as well as cultivated and atrial ECs (EC-2/Cl5: mainly cultivated ECs) were arranged at opposing ends of trajectorial pseudospaces (Suppl Fig. S9A). We further analyzed the trajectory branches according to their associated top-specific DEGs. For this, we performed an additional subcluster analysis in Seurat (Suppl Fig. S9B). Subcluster 12 mapped to trajectory 1, subclsuter 0 to trajectory 2, etc. We found reasonable gene expression and GO terms for CF trajectories (Tr 1, 2, 3) as well as EC trajectories (Tr 8, 9) (Suppl Fig. S9C, D). CF trajectories 1 and 2 are highly similar to the trajectory branches 1 and 2 in Fig. 3D. Tr 3 and Tr 9 are the trajectory end points of the cultivated CFs and ECs, respectively, exhibiting terms such as “collagen containing extracellular matrix” (CF Tr 3) or “cell cycle” (EC Tr 9) (Suppl Fig. S9D). Finally, we analyzed RNA velocity by Velocyto in this setting. We found that CFs and ECs were each linked to their atrial equivalent (atrial CFs (CF-1/2), atrial ECs (EC-1)) by velocity streamlines (arrows in Suppl Fig. 9E). The directions of the arrows pointed from the cultivated cells to the atrial cells (Suppl Fig. S9E). Then, by analyzing CDCs and atrial samples, we found that CDCs were directly linked to the CF-1 population by velocity streamlines (Fig. 3F, arrows) indicating that CDCs might originate from CFs. The bulk part of CDCs (77% of CDCs in Cluster CDC-1/CF-1) seemed to act like immature CFs since the direction of the arrows points from CDCs to CFs. The smaller part of CDCs (23% of CDCs in the cluster CDC-2) seemed to develop more into the direction of ECs (arrows from CDC-2 point into the direction of EC), even if they are still far away from those.

### Comparison of molecular characteristics of infant and adult CDCs

Preclinical studies with human CDCs derived from neonatal or adult atrial appendages revealed that neonatal CDCs had stronger repair abilities than adult CDCs after transplantation in immunodeficient infarcted rat hearts. Neonatal CDCs maintained myocardial function, prevented adverse remodeling and promoted angiogenesis [53]. We sought to elucidate whether this might be attributable to certain molecular characteristics of “infant” CDCs (age: 5 days—5 years, Suppl Table 2) compared to “adult” CDCs (age: 55–76 years, Suppl Table 1).

**Fig. 4:**
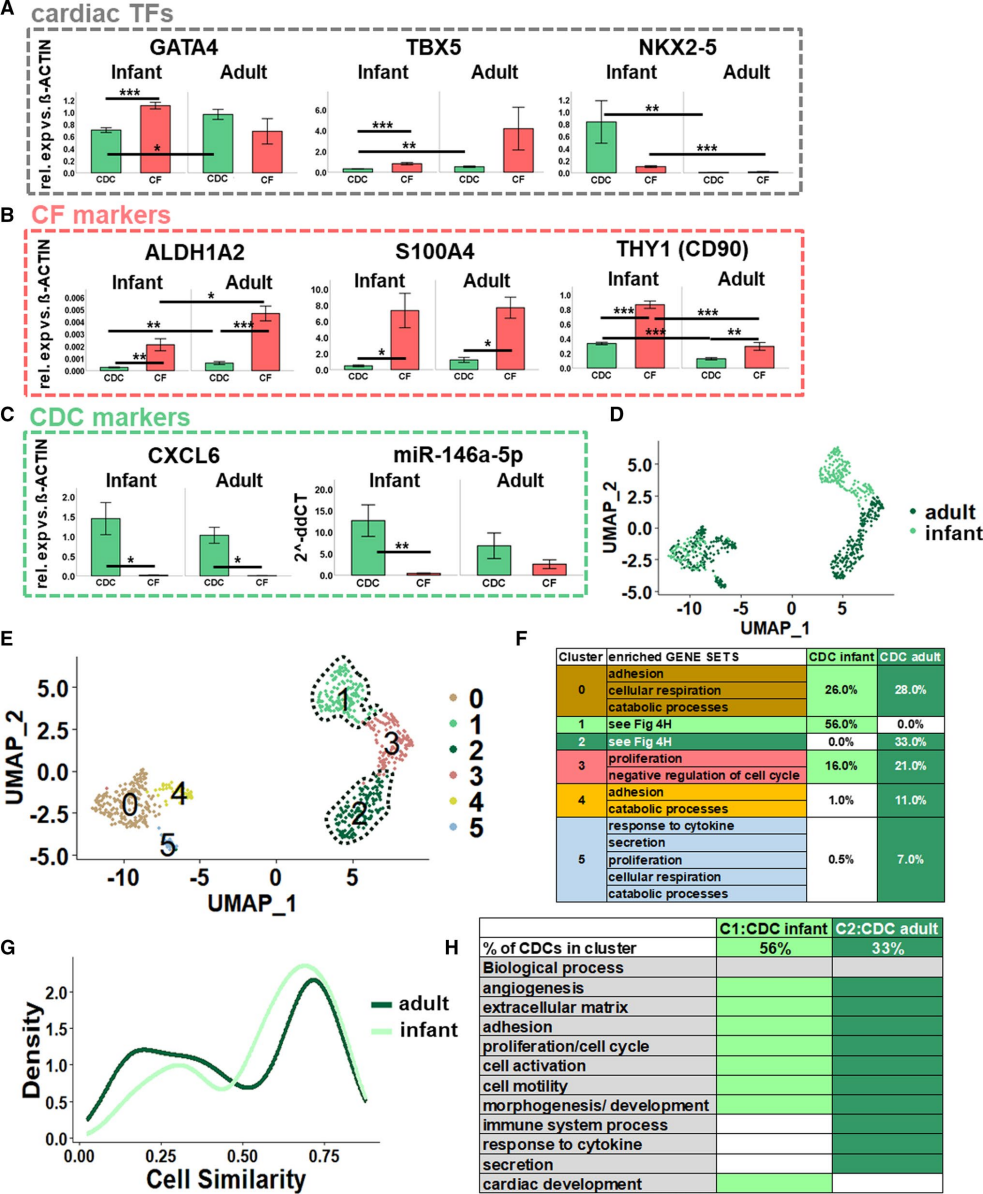
Comparison of molecular characteristics of adult and infant CDCs. **A–C** Gene expression analysis by qRT-PCR of CDCs and CFs derived from infant (age: 5 days–5 years) and adult (age: 55–76 years) patients. Relative RNA expression versus β-ACTIN (gene symbol: *ACTB*) is illustrated. Data are represented as mean ± SEM, *p < 0.05, **p < 0.01, ***p < 0.001 (all p-values in Suppl Table 11) **D-E** Single-cell RNA sequencing of infant (age: 7 days) and adult (age: 61 years) CDCs. UMAP plots are illustrated colored by sample identifier (**D**) or cluster defined by gene expression (**E**). **F** Gene set topics enriched for uDEGs of each of the five clusters from Fig. 2E (for a detailed analysis see Suppl Table 12). **G** Transcriptional similarity plots of infant and adult CDCs generated from sc-RNAseq data. **H** Gene set topics enriched for uDEGs of infant CDC-specific cluster 1 and adult CDC-specific cluster 2 (for a detailed analysis see Suppl Table 13)

Interestingly, gene expression analysis showed lower expression of the cardiac transcription factors *GATA4* and *TBX5* in infant CDCs compared to adult CDCs, and infant CFs, respectively (Fig. 4A, left and middle panel). In contrast, *NKX2-5* was significantly higher expressed in the infant group (CDCs and CFs) (Fig. 4A, right panel). CF marker *ALDH1A2* was upregulated in adult cells compared to infant cells, and CFs compared to CDCs in both age groups (Fig. 4B, left panel). *S100A4* was also upregulated in CFs versus CDCs in both age groups (Fig. 4B, middle panel). Comparing corresponding cell types, *THY1* (*CD90)* expression was interestingly higher in the infant group (Fig. 4B, right panel). *CXCL6* was specific for CDCs in both age groups while miR-146a proved to be specific in CDCs compared to CFs in the infant group only (Fig. 4C).

To analyze molecular differences of infant and adult CDCs in more detail, a neonatal CDC sample (patient age: 7 days) was compared to the adult single-cell CDC sample (patient age: 61 years, see also Fig. 2) by sc-RNAseq. Quality control of infant and adult CDCs was performed by morphological evaluation and *ALDH1A2* (specific CF-marker) expression compared to CFs generated from the same patient (Suppl Fig. S10A, B). After low-quality cells were filtered out (Suppl Fig. S10C–E, Suppl Table 6), 2069 cells were analyzed with a median of 5649 genes and 33,731 UMI counts per cell (Suppl Fig. S10F, G). As already noticed for adult CDCs, infant CDCs also exhibited a high amount of mitochondrial genes (Suppl Fig. S10E, Suppl Table S7).

Unsupervised global subclustering of the two CDC-samples (infant and adult, Fig. 4D) according to their specific gene expression subdivided the CDCs into six clusters (Fig. 4E, Suppl Table 12). Mainly two clusters (0, 3) overlapped between the infant and adult sample (Fig. 4F). The cells in these clusters were enriched for GO-terms such as adhesion, cellular respiration, cell cycle and catabolic processes (Fig. 4F). The small clusters 4 and 5 mainly consisted of adult CDCs and corresponded to biological processes such as “response to cytokine”, secretion but also adhesion, cellular respiration, cell cycle and catabolic processes equally to cluster 0 and 3 (Fig. 4F). The high similarity between infant and adult CDC samples was confirmed by calculating cell similarity [65] (Fig. 4G) and *CXCL6* was again found to be expressed in all CDCs (Suppl Fig. 10H). However, cluster 1 (56% of infant CDCs) was specific for infant CDCs while cluster 2 (33% of adult CDCs) was only observed in adult CDCs (Fig. 4D, E, Suppl Table 12). Interestingly, GSEA revealed several similar GO terms for both clusters (Suppl Table 13, Fig. 4H). Infant as well as adult CDCs from cluster 1 and 2 were involved into angiogenesis, extracellular matrix organization, adhesion, cell cycle processes, cell activation, cell motility and developmental processes. The main differences between cluster 1 (infant CDCs) and cluster 2 (adult CDCs) were immunomodulatory properties, “response to cytokine” and secretion for adult CDCs while only infant CDCs exhibited GO terms related to cardiac development (Fig. 4H). Exemplary violin and gene expression plots (*WNT5A*, *CSF3*), as well as heat maps confirmed those results (Suppl Fig. S10I–L).

In summary, sc-RNAseq analysis revealed cardiac developmental processes in neonatal CDCs while adult CDCs were more involved in immunomodulation and secretion.

### Functional effects of CDC-derived extracellular vesicles (EVs) compared to CF-derived EVs

To analyze paracrine effects of CDCs such as pro-angiogenic and anti-fibrotic effects or inhibition of cardiomyocyte apoptosis [28, 35, 64], we evaluated the impact of CDC-EVs on different cardiac cell types. Effects of CDC-EVs were directly compared to CF-EVs. The comparison of infant and adult CDC- and CF-EVs further allowed us to assess age-dependent effects.

EVs were isolated from serum-free medium conditioned by CDCs or CFs for seven days (Suppl Fig. S11A). Fewer than 5% of dead cells were detected after one week in serum-free medium for CDCs and CFs (Suppl Fig. S11B). Although no significant difference of cell numbers of CDCs and CFs per flask was observed (Suppl Fig. S11C), nanoparticle tracking revealed that CDCs secreted a significant higher particle number compared to CFs (Suppl Fig. S11D) corresponding to previous results of GSEA (GO terms associated with secretion, Suppl Table 8). Most EVs of CDCs and CFs from infants and adults ranged in size from 40 to 200 nm (mean: 140 nm), indicating that mainly exosomes and not apoptotic bodies were isolated [26, 50] (Suppl Fig. S11E, F). Presence of typical exosomal surface markers CD63 and CD81 [61] was confirmed by flow cytometry of selected CDC-EV preparations (Suppl Fig. S11G). MiR-146a was upregulated in CDC-EVs compared to CF-EVs both in infant and adult samples (Suppl Fig. S11H), whereas neither miR-132 nor miR-21 showed significant differences between cell types or age groups (Suppl Fig. S11I, J).

Functional effects of CDC- and CF-EVs on various cardiac cell types were assessed using well established in vitro assays. For all assays, cells were either seeded in their normal growth medium (positive control, PosCtr), serum-free medium (negative control, NegCtr) or serum-free medium supplemented with infant or adult CDC- or CF-EVs.

**Fig. 5:**
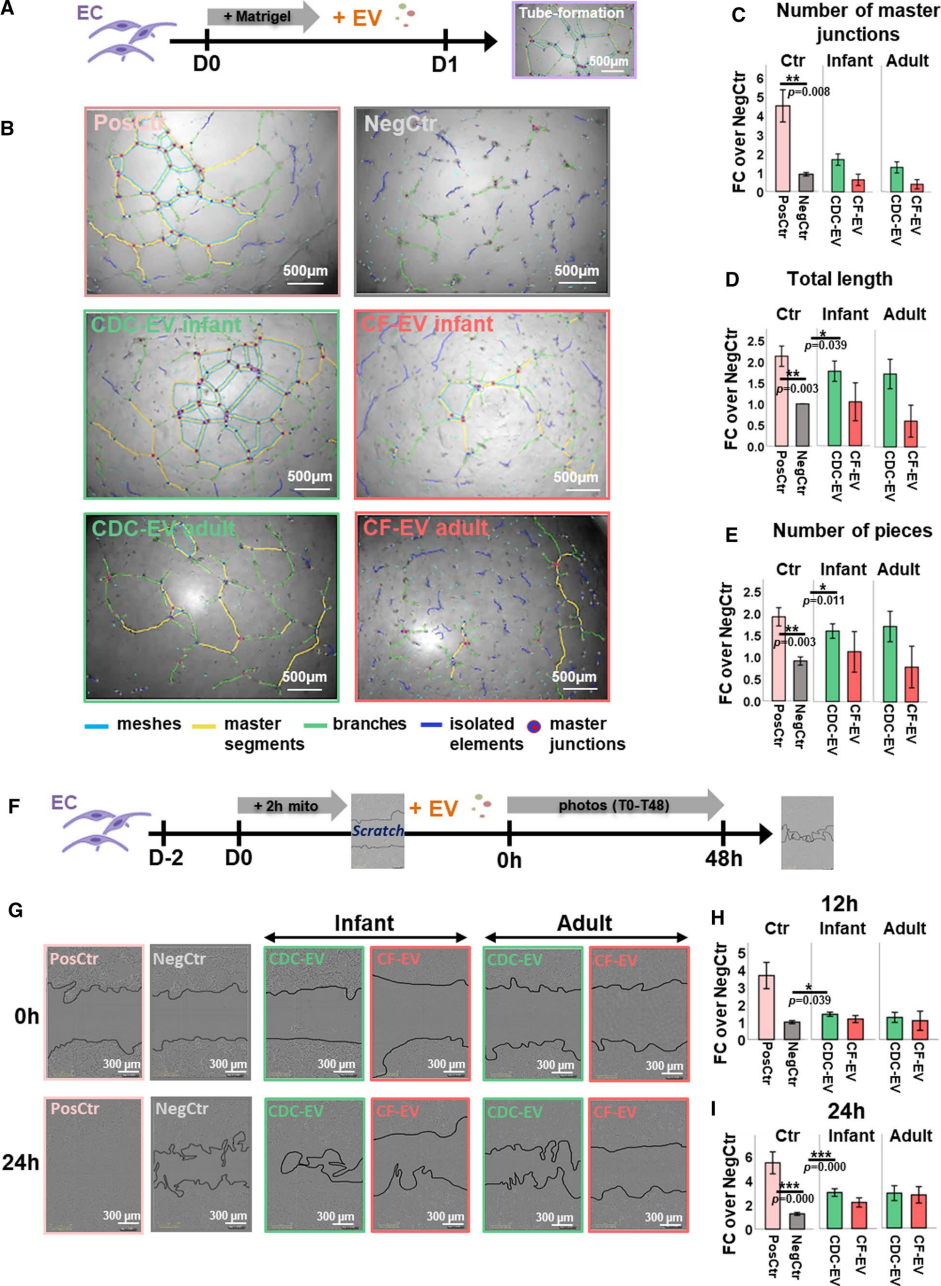
Paracrine effects mediated by CDC- and CF-derived extracellular vesicles (EVs). **A–F** Tube formation assay with human ECs on matrigel (matrigel assay). **A** Experimental outline. **B** Exemplary pictures of the positive control (PosCtr, EC medium with supplements), the negative control (NegCtr, serum-free medium) and serum-free medium supplemented with infant/adult CDC-/CF-EVs at the end of the matrigel assay. Angiogenesis Analyzer (ImageJ) highlights structures such as master segments, branches, isolated elements and master junctions. The software also calculates parameters such as “Total length” (the sum of length of segments, isolated elements and branches in the analyzed area), “Total master segments length” (the sum of the length of the detected master segments in the analyzed area), “Number of pieces” (the sum of number of segments, isolated elements and branches detected in the analyzed area). Detected and calculated parameters were normalized to the negative control (fold change to negative control, “FC over NegCtr”). **C–E** Quantitative analysis of selected parameters. **F–I** Migration assay (scratch assay) with human ECs. **F** Experimental outline. **G** Exemplary pictures of ECs incubated with EC medium with supplements (PosCtr), serum-free medium (NegCtr), or serum-free medium supplemented with CDC and CF-EVs at the time point of the scratch (0 h) and 24 h later. **H–I** Comparison of the differences of the cell-free area between time point 0 and 12 h (**H**) or 24 h (**I**) normalized to the negative control (fold change to negative control, “FC over NegCtr”). Data are represented as mean ± SE, *p < 0.05, **p < 0.01, ***p < 0.001

To study angiogenesis in vitro, tube formation assays on matrigel and migration assays with primary human ECs were utilized (Fig. 5A–I).

The tube formation assay is based on the ability of ECs to differentiate into tube-like structures on matrigel [47], an important prerequisite for vessel formation [15]. Primary ECs were seeded on matrigel and an ImageJ angiogenesis analyzer tool [1, 10] assessed the extent of tube formation on the next day (Fig. 5A). Performing the assay with infant and adult CDC- and CF-EVs showed that EC tube formation was significantly promoted by infant CDC-EVs, but not CF-EVs (Fig. 5B–E). Adult CDC-EVs tended to perform in a similar manner than infant CDC-EVs but did not reach significance.

The ability of ECs to migrate constitutes another important angiogenic factor (scratch assay). After EC proliferation was prevented by mitomycin C, a scratch was conducted in the EC layer. The closure of the scratch was observed over 48 h (Fig. 5G). Comparing the effects of infant and adult CDC- and CF-EVs, only infant CDC-EVs significantly promoted EC migration (Fig. 5H–J, Suppl Videos V1–6). However, adult CDC-EVs acted similar than infant CDC-EVs but did not reach significance.

Notably, in both EC assays (tube formation and scratch assay), no significant difference between CDC-EVs and CF-EVs was observed.

Several studies reported the ability of CDC-EVs to decrease cardiomyocyte apoptosis [35, 64]. Gene expression of the death surface receptor *Fas* [66] and the pro-apoptotic *Bax* assessed the anti-apoptotic effect of CDC- and CF-EVs on neonatal rat cardiomyocytes (NRCMs). Quality of NRCMs was demonstrated by the expression of cardiomyocyte markers (Suppl Fig. S12A, B). Only beating NRCMs were used for apoptosis assays (Suppl Video V7). Cobalt chloride treatment induced NRCM apoptosis and then either NRCM growth medium (PosCtr), serum-free medium (NegCtr) or serum-free medium supplemented with infant/adult CDC/CF-EVs was added for four days (Suppl Fig. S12C). Pro-apoptotic *Bax* was significantly reduced in NRCMs by all EVs (CDC and CF, adult and child) compared to the negative control (Suppl Fig. S12D) while the death surface receptor *Fas* was only significantly reduced by adult CF-EVs (Suppl Fig. S12E).

Since anti-fibrotic properties of CDC-EVs have been previously described [23, 35, 64], we assessed this effect in a so-called wound healing assay (scratch/migration assay with CFs). Migration of CFs is a relevant process during scar building after injury in the heart. CF scratch assays were performed analogically to the above-described EC scratch assay (Suppl Fig. S12E). No significant effect was observed, neither for infant nor adult CF- or CDC-EV-treated samples (Suppl Fig. S12F, G, Suppl Videos V8–13). However, adult as well as infant CDC-EVs stimulated CF migration to a certain extent (Suppl Fig. S12F, G) indicating a potential for wound healing/scarring, even if the effect was not significant.

In summary, CDC-EVs promoted endothelial tube formation and migration. CF-EVs of the corresponding age groups did not replicate these effects. CDC-EVs stimulated wound healing/scarring to a certain extent. CDC-EVs as well as CF-EVs reduced gene expression of the pro-apoptotic marker *Bax* in NRCMs.

### Assessment of sphere formation as a prerequisite for regenerative characteristics

As we hypothesized that CDCs develop their properties mainly by the exposure to growth factors and 3D-sphere formation, we assessed whether a similar cell type could be generated when treating CFs alike (Suppl Fig. S13A). Therefore, CFs were replated to poly-d-lysine-coated plates with the same growth factor mix than CDCs. Interestingly, this resulted in 86% of experiments in sphere formation which had a similar appearance and size than CDC cardiospheres (Suppl Fig. S13B). Cells grown out of CF-derived spheres were termed CF sphere-derived cells (CFSPhs) (Suppl Fig. S13A, B). CFSPhs were generated from neonatal patients only and compared to CDCs and CFs of the same age group (≤ 21 days). Gene expression of cardiac transcription factors, CF- and CDC-markers showed that CFSphs became more similar to CDCs than to their originating CF population (Suppl Fig. S13C–F). They lost their fibroblast identity and adopted a more CDC-like phenotype (Suppl Fig. S13E, F). Merely, *NKX2.5* was expressed in an even lower level in CFSPhs than in CFs (Suppl Fig. S13C, right panel). Flow cytometry showed ubiquitous abundance of CD90 and CD105 in CDCs, CFSPhs and CFs (Suppl Fig. S13G, H). Immunocytochemical staining revealed no considerable difference between CFSPhs and CDCs or CFs regarding the abundance of DDR2 and CD90 (Suppl Fig. S13I, J).

To assess whether CFSPh-EVs had comparable effects to CDC-EVs, we isolated EVs according to the same protocol as for CDC- and CF-EVs (Suppl Fig. S11A). Percentage of dead cells and cell number per culture flask were comparable between CFSPhs, CDCs and CFs (Suppl Fig. S13K, L). However, CFSPh-EV yield approached the yield of CDC-EVs (Suppl Fig. S13M). CFSPh-EV size ranged between 40 and 200 nm, as seen for CDC- and CF-EVs (Suppl Fig. S13N, Suppl Fig. S11E). The expression level of miR-146a of CFSPh-EVs was between CDC-EVs and CF-EVs (Suppl Fig. S13O).

Finally, we compared CDC-, CF- and CFSPh-EVs by assessing their function as described above (tube formation assay, scratch assay with ECs and CFs). Results revealed that CFSPh-EVs were not significantly more beneficial than the NegCtr concerning their potential to augment angiogenesis assessed by tube formation assays (Suppl Fig. S14A–G, Suppl Video V14). However, they tended to stimulate EC migration to a similar level than CDC-EVs, without reaching significance (Suppl Fig. S14E–G). Further, CFSph-EVs did not promote migration of CFs, similar than CDC-EVs (Suppl Fig. S14H, I, Suppl Video V15).

Thus, incubation of CFs with growth factors and their cultivation in three-dimensional culture conditions (spheres), altered their gene expression, making them more similar to CDCs, but did not augment their EV-mediated angiogenesis potential to the same level of CDC-EVs.

## Discussion

In this study, we sought to reveal the elusive molecular identity of CDCs compared to other cardiac non-myocyte cell types, namely CFs, SMCs and ECs. In addition, the cellular origin of CDCs should be pursued by investigating human right atrial biopsies at the single-cell level.

Expression analysis of selected marker genes showed that CDCs did not exclusively express published CDC-markers compared to non-myocyte cell types. CD105, for example, was equally upregulated in all analyzed cell types (AFs, CFs, SMCs and ECs) or miR-146a was also highly expressed in CFs. In fact, CDCs depicted similar molecular characteristics like non-myocyte cells.

**Fig. 6:**
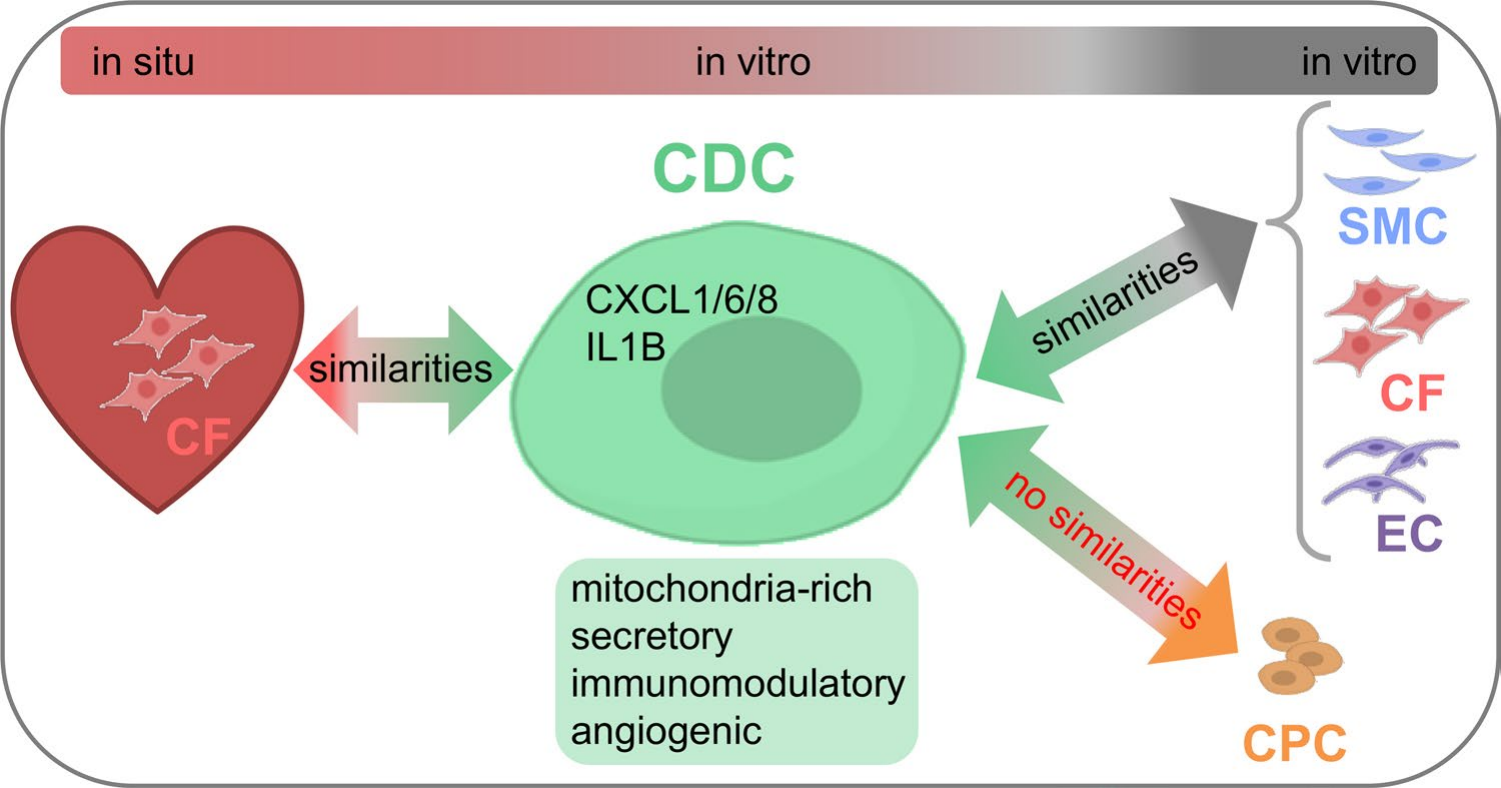
Molecular identity of CDCs. CDCs were identified as a distinct non-myocyte, non-hematopietic cell type with metabolic (mitochondria-rich), proliferative, secretive and immunomodulatory characteristics. CDCs originate from the human heart and show high similarity to atrial cardiac fibroblasts (CFs) in the human heart. Smooth muscle cells (SMCs), CFs and endothelial cells (ECs) shared biological processes with CDCs while cardiac progenitor cells (CPCs) did not. In functional assays, CDC-EVs acted in a pro-angiogenic way. Parts of the figure were created with Biorender.com

Sc-RNAseq analysis for cultivated human primary cells (CFs, SMCs, ECs and CDCs) revealed a detailed picture of their molecular characteristics. CDCs represented a mitochondria-rich cell type (high amount of mitochondrial genes) and were distinguished from CFs, SMCs and ECs mainly by their secretory and immunomodulatory characteristics (Fig. 6). Cells with an enhanced mitochondrial content have higher energy needs, like for example fat cells or muscle cells including cardiomyocytes. Interestingly, activated fibroblasts/myofibroblasts also increase mitochondrial respiration [6]. Cytokine-dependent activation of fibroblasts to myofibroblasts is accompanied by phenotypic changes including increased secretory and contractile properties [46]. These changes are dependent on increased energy utilization involving an increase of mitochondrial respiration as well as an increase of mitochondrial content [46].

By analyzing the Top 10 specific markers of CF, SMC, EC and CDCs *CXCL1/6/8* and *IL1B* could be defined as new specific markers for CDCs. These genes are all involved in inflammatory processes, either as chemokine ligands (*CXCL1/6/8*) or cytokines (*IL1B*). However, as shown in Fig. 3C these markers were also expressed in macrophages, dendritic cells and mast cells of human atrial biopsies (e.g. *CXCL1, CXCL8, IL1B*). Merely, *CXCL6* seemed to be specific for CDCs. Interestingly, *CXCL6* was recently highlighted as an important paracrine factor in the secretome of human myocardial c-KIT positive cells [63]. *CXCL6* was reported to play relevant roles in migration as well as angiogenesis in those cells [63]. This might be, at least partially, responsible for the angiogenic potential of infant and adult CDCs demonstrated in Fig. 5.

Thus, it can be summarized that CDCs exhibit a certain similarity to non-myocyte cell types, but are rather a unique cell type involved in a variety of immunomodulatory, proliferative, secretory and metabolic processes. Additionally, highlighted by the sc-RNAseq data, the exposure to growth factors such as EGF, FGF and Cardiotrophin made CDCs stand out by augmented cytokine expression and enhanced vesicle secretion (e.g. high EV release per flask compared to CFs, Suppl. Fig. S11D). Particularly, bFGF is known to induce proliferation of fibroblasts and SMCs [20, 36] and to interplay with the cytokine IL1B in many infectious and inflammatory diseases [31, 59]. EV secretion processes represent cell–cell communication and are also important for cytokine exchange [21]. CDCs have been previously reported to secrete angiopoetin-2, bFGF, HGF, IGF-1, SDF-1 and VEGF [37] and to be positively influenced by growth factors such as EGF, increasing their migration activities [2], and bFGF, promoting their engraftment in vivo [58]. Taken together, certain culture conditions could augment a potential regenerative capacity of non-myocyte cell types by altering their secretome. Indeed, exposure of CFs to the same medium/growth factor cocktail generated a cell type (CFSPhs) with partially similar gene expression patterns than CDCs. In summary, the unique phenotype of CDCs and their potential regenerative abilities might be the result of their exposure to high concentrations of a variety of medium supplements, which make them acquire the characteristics of an inflammatory non-myocyte cell type.

By further comparing sc-RNAseq data of CDCs to sc-RNAseq data of differentiating hESCs it was clearly shown that CDCs do not adopt the characteristics of veritable cardiac progenitor cells or early cardiomyocytes. CDCs have originally been postulated to exhibit characteristics of cardiac progenitor cells and to be able to partially differentiate into cardiomyocytes [43]. Our study, however, clearly showed the similarity of CDCs to cardiac non-myocyte cell types rather than to cardiac progenitor cells. Cardiac transcription factors typical for cardiac progenitor cells (e.g. *TBX5*, *NKX2-5*) were much higher expressed in hiPSC-derived cardiac progenitor cells (DIFF D6/D8) than in CDCs. Morphologically, CDCs had a fibroblast-like appearance (see immunocytochemistry data) and beating of CDCs or cardiospheres was not observed, unlike described earlier [43].

To unveil the originating cell population of CDCs in the human heart we analyzed four human right atrial biopsies at the single cell and single nuclei level. UMAP plots positioned CDCs next to one of the CF clusters. The bulk part of CDCs even overlapped with this CF cluster. CMs, ECs and SMCs were positioned at greater distances. Trajectory and RNA velocity analyses supported the similarity between CDCs and atrial CFs and predicted that they might be two different cell state transitions of the same cell type, thus indicating that atrial CFs might be the originating cell population of CDCs. However, to finally confirm this statement, further experiments such as lineage-tracing analysis with fibroblast reporter mice would be necessary throughout the CDC cultivation protocol. Unfortunately, interpretation of such experiments might be limited by the lack of specific molecular markers for CFs [45].

Comparing neonatal and adult CDCs by sc-RNAseq analysis revealed cardiac developmental processes only in neonatal CDCs. That might be one reason why neonatal CDCs showed more beneficial effects after transplantation in infarcted rat hearts compared to adult CDCs by maintaining myocardial function, preventing adverse remodeling and promoting angiogenesis [53].

Functional in vitro assays with CDC- and CF-derived EVs analyzed previously described beneficial properties of CDCs, such as pro-angiogenic effects [14, 28, 64], anti-fibrotic effects [35] and anti-apoptotic effects [64]. In our study, CDC-EVs promoted endothelial tube formation and endothelial migration corresponding to the angiogenic potential detected by sc-RNAseq. CF-EVs did not replicate the effects of CDC-EVs.

CDC-EVs as well as CF-EVs reduced the expression of the apoptosis marker *Bax* in NRCMs. When we analyzed apoptosis reduction capabilities of EVs we concentrated on gene expression analysis. This might not be the state-of-the-art method (such as TUNEL assays), however, other publications also analyzed gene expression for apoptosis detection [32].

Recently uncovered immunomodulatory effects of CDCs, describing their role in polarizing macrophages away from the pro-inflammatory M1-phenotype towards a cardioprotective phenotype after MI [16, 17], were not investigated in this study. De Couto et al. [16, 17] suggested that the positive effects of CDCs (e.g. anti-apoptotic effects) were mainly mediated by macrophages. That fact could be an explanation why we did not find large effects in our functional assays. However, other manuscripts also used similar assays like we did and detected positive functional effects of CDCs [64].

## Conclusion

In this study, sc-RNAseq disclosed CDCs as a unique cell type, but with clear similarities to cardiac non-myocyte and non-hematopoietic cells. CDCs were defined as a mitochondria-rich, non-myocyte and non-cardiac progenitor cell type influenced by cell culture conditions (growth factors, 3D cultivation). By this, CDCs adopted a highly proliferative, secretory, and immunomodulatory phenotype mirroring the characteristics of inflammatory cell types such as myofibroblasts. Atrial CFs might be the originating cell population of CDCs in the human heart since they showed highly similar transcriptional profiles. However, using above mentioned culture conditions CDCs earned some kind of bioactivity (e.g. gained some angiogenic potential) which allows them to act disease modifying in certain disorders.

### Limitations of the present study

Finally, we have to mention some limitations of our study.

In the present study, we generated CDCs from human right atrial tissue according to the protocol used by Messina et al. [43]. The same protocol was utilized for the generation of CDCs from children with single ventricle physiology in the TICAP and PERSEUS trial, where significant functional improvement of the right ventricular ejection fraction was detected [29, 30, 51]. However, recent clinical trials in adult patients, such as HOPE (Duchenne muscular dystrophy patients), DYNAMIC (dilated cardiomyopathy patients), ALLSTAR (ischemic left ventricular dysfunction patients) or the trial with Covid-19 patients [11, 40, 54, 60] all used an allogenic CDC cell product (CAP-1002) that was generated from ventricular tissue (see also [55]). These trials showed variable types of disease-modifying bioactivity of CAP-1002. However, we could not find a publication about a direct comparison of “atrial” versus “ventricular” CDCs and which cell type showed more beneficial effects. Thus, the results of our study cannot be compared directly to the CAP-1002 CDCs. Nevertheless, our results might elucidate the cellular origin of CDCs in the heart and their molecular identity compared to non-myocyte, non-hematopoietic cell types.

## Supplementary Information

Below is the link to the electronic supplementary material.

Supplementary file1 (PDF 5394 KB)

Supplementary file2 (MP4 809 KB)

Supplementary file3 (MP4 818 KB)

Supplementary file4 (MP4 804 KB)

Supplementary file5 (MP4 569 KB)

Supplementary file6 (MP4 563 KB)

Supplementary file7 (MP4 319 KB)

Supplementary file8 (MP4 482 KB)

Supplementary file9 (MP4 390 KB)

Supplementary file10 (MP4 473 KB)

Supplementary file11 (MP4 473 KB)

Supplementary file12 (MP4 707 KB)

Supplementary file13 (MP4 671 KB)

Supplementary file14 (MP4 667 KB)

Supplementary file15 (MP4 468 KB)

Supplementary file16 (MP4 463 KB)

## Data Availability

The authors declare that all supporting data are available within the article and its supplementary information files. The accession number for the sc-RNAseq data of CFs, ECs, SMCs, CDC_adult_ and CDC_infant_ reported here is: GEO:GSE149699 (https://www.ncbi.nlm.nih.gov/geo/). The sc-RNAseq (RA-3, RA-4) data of human right atrial biopsies can be found here: GEO:GSE149699 (https://www.ncbi.nlm.nih.gov/geo/). sn-RNAseq (RA-1, RA-2) data of human right atrial biopsies were already published [34] and are reported here: GEO GSE126128 (https://www.ncbi.nlm.nih.gov/geo/).
