# Supplementary material for: Uncovering the molecular identity of cardiosphere-derived cells (CDCs) by single-cell RNA sequencing
Source: Basic Res Cardiol. 2022 Mar 8;117(1):11. doi: 10.1007/s00395-022-00913-y (PMC8902493; doi:10.1007/s00395-022-00913-y)
Supplement: Supplementary file 1 — Supplementary file1 (PDF 5394 KB) [file 395_2022_913_MOESM1_ESM.pdf]

SUPPLEMENTAL MATERIAL

Uncovering the Molecular Identity of Cardiosphere-Derived Cells (CDCs) by Single Cell RNA Sequencing

running head: Molecular Identity of CDCs

Palgit-S. Kogan[1,2,§], Felix Wirth[1,2,§], Archana Tomar[4], Jonatan Darr[4], Raffaele Teperino[4], Harald Lahm[1,2], Martina Dreßen[1,2], Nazan Puluca[1,2], Zhong Zhang[1,2], Irina Neb[1,2], Nicole Beck[1,2], Tatjana Luzius[1,2], Luis de la Osa de la Rosa[1,2], Kathrin Gärtner[5], Corinna Hüls[5], Reinhard Zeidler[5,6], Deepak Ramanujam[3,7], Stefan Engelhardt[3,7], Catharina Wenk[8], Lesca M. Holdt[8], Mimi Mononen[9], Makoto Sahara[9,10], Julie Cleuziou[11,12,13], Jürgen Hörer[11,12], Rüdiger Lange[1,2,3], Markus Krane[*,1,2,3], Stefanie A. Doppler[*,1,2]

1. **Supplemental Figures and Supplemental Figure Legends**
2. **Supplemental Video Legends**
3. **Supplemental Tables**
4. **Supplemental Materials and Methods Section**
5. **Supplemental References**

1. Supplemental Figures and Figure Legends

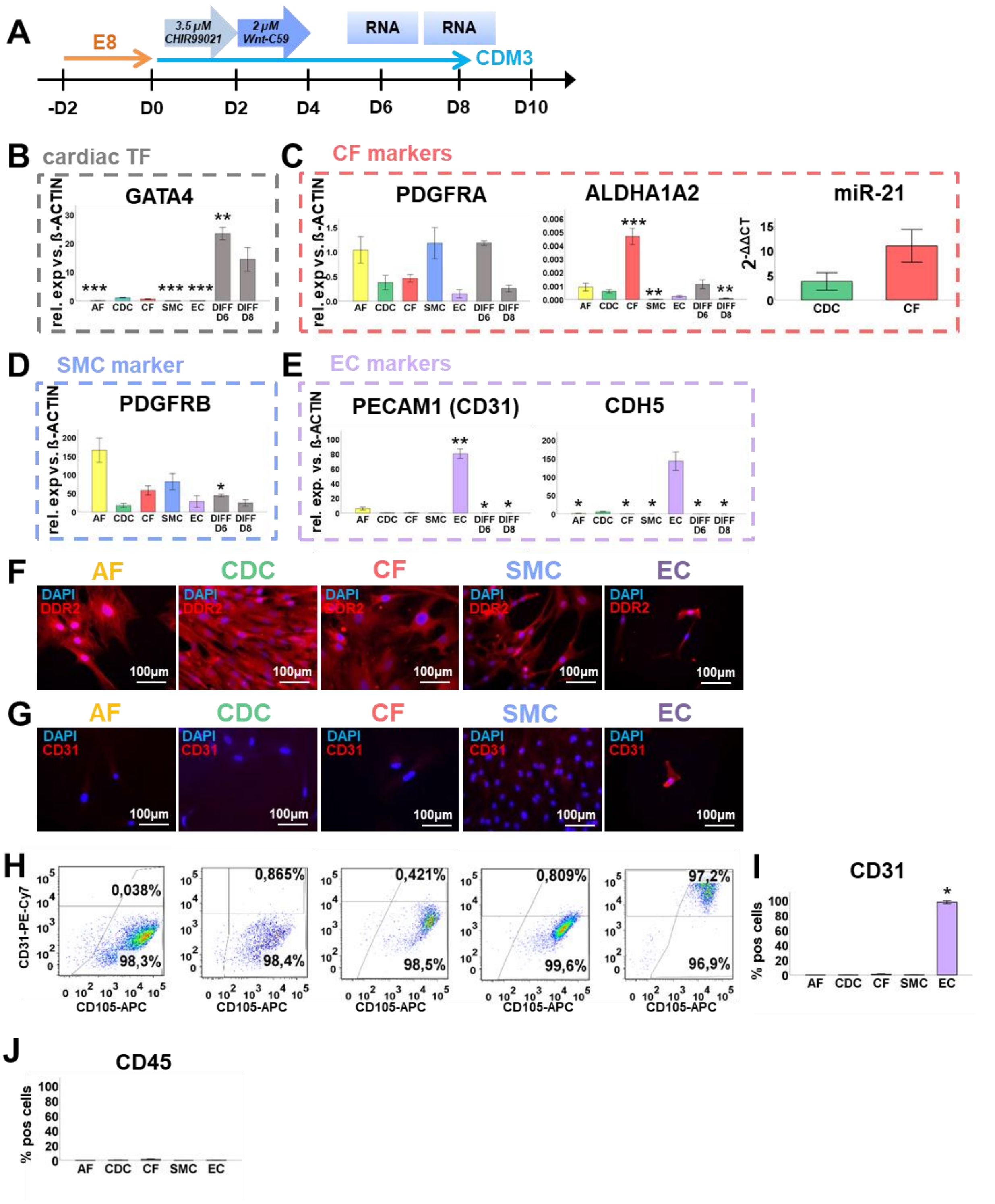

<u>Supplemental Figure S1: Molecular characterization of CDCs compared to primary cells</u>. A) Protocol of a cardiac-directed differentiation of human induced pluripotent cells (hiPSCs)

manipulating the Wnt pathway. Cell lysates for RNA isolation were taken at day 6 and 8 (DIFF D6/8). Abbreviations: E8, CDM3: medium (see methods). **B-E)** Gene expression analysis of CDCs compared to primary cells and hiPSCs-derived cardiac progenitor cells (DIFF D6) or immature cardiomyocytes (DIFF D8). Relative RNA expression versus β-ACTIN (gene symbol: *ACTB*) is illustrated for **B)** the cardiac transcription factor (TF) *GATA4*, **C)** cardiac fibroblast (CF) markers *PDGFRA*, *ALDH1A2* and miR-21, **D)** smooth muscle cell (SMC) marker *PDGFRB*, and **E)** endothelial cell (EC) markers *PECAM1 (CD31)* and *CDH5*. Abbreviations: AF adipose tissue-derived fibroblasts. **F)** Immunocytochemical (ICC) staining against the fibroblast marker DDR2 shows ubiquitous expression in all cell types. **G)** ICC staining against CD31 only revealed expression in ECs. **H-I)** Flow cytometry analysis with antibodies against CD31 (conjugated with PE-Cy7) confirmed ICC results: Exemplary dot plots (**H**) and percentage of CD31-positive cells (**I**). **J)** Absent expression of the hematopoietic marker CD45 in all cell types was verified by flow cytometry.
Data are represented as means +/- SEM, * $p < 0.05$, ** $p < 0.01$, *** $p < 0.001$ (only significances against CDCs are depicted). For a complete overview of *p*-values, see Suppl Table 3 (qRT-PCR) and Suppl Table 4 (Flow cytometry).

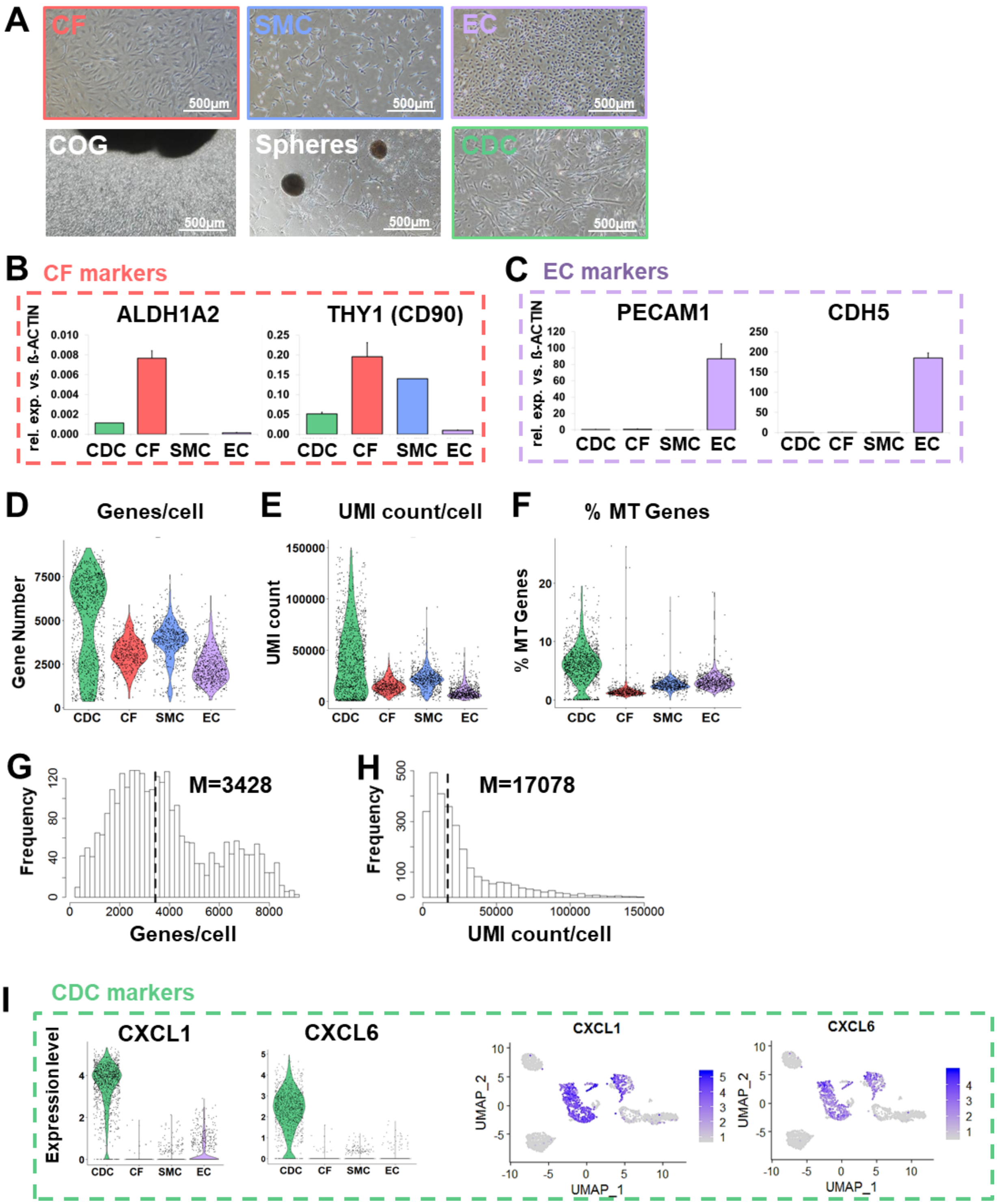

Supplemental Figure S2: Quality control of cell samples used for single-cell RNA sequencing (sc-RNAseq) **A)** Morphological assessment of the utilized cell samples (adult CDCs, CFs, SMCs, ECs) by phase contrast microscopy directly before the sc-RNAseq experiment. Abbreviations: CF cardiac fibroblasts, SMC smooth muscle cells, EC endothelial cells, COG cardiac outgrowth, CDC cardiosphere-derived cells. **B-C)** Gene expression profiles (relative RNA expression versus β-

ACTIN) of CF **(B)**, and EC **(C)** markers of the cell samples used for the sc-RNAseq experiment (adult CDCs, CFs, SMCs, ECs). Bar diagrams show means +/- SEM. **D-F)** Violin plots of detected gene number per cell (Genes/cell) (**D**), unique molecular identifier count per cell (UMI count/cell) (**E**) and percentage of mitochondrial genes per cell (% MT Genes) (**F**), illustrated for each cell sample analyzed after filtering procedure (median values in Suppl Table 7) **G-H)** Histograms illustrating the frequency of genes/cell (**G**) or the frequency of UMI count/cell (**H**) of the filtered merged CCA-corrected Seurat object containing CDCs, CFs, ECs and SMCs. Abbreviations: M median. **I)** Violin plots and UMAP plots generated from sc-RNAseq data for uDEGs in the CDC sample (CXCL1, CXCL6).

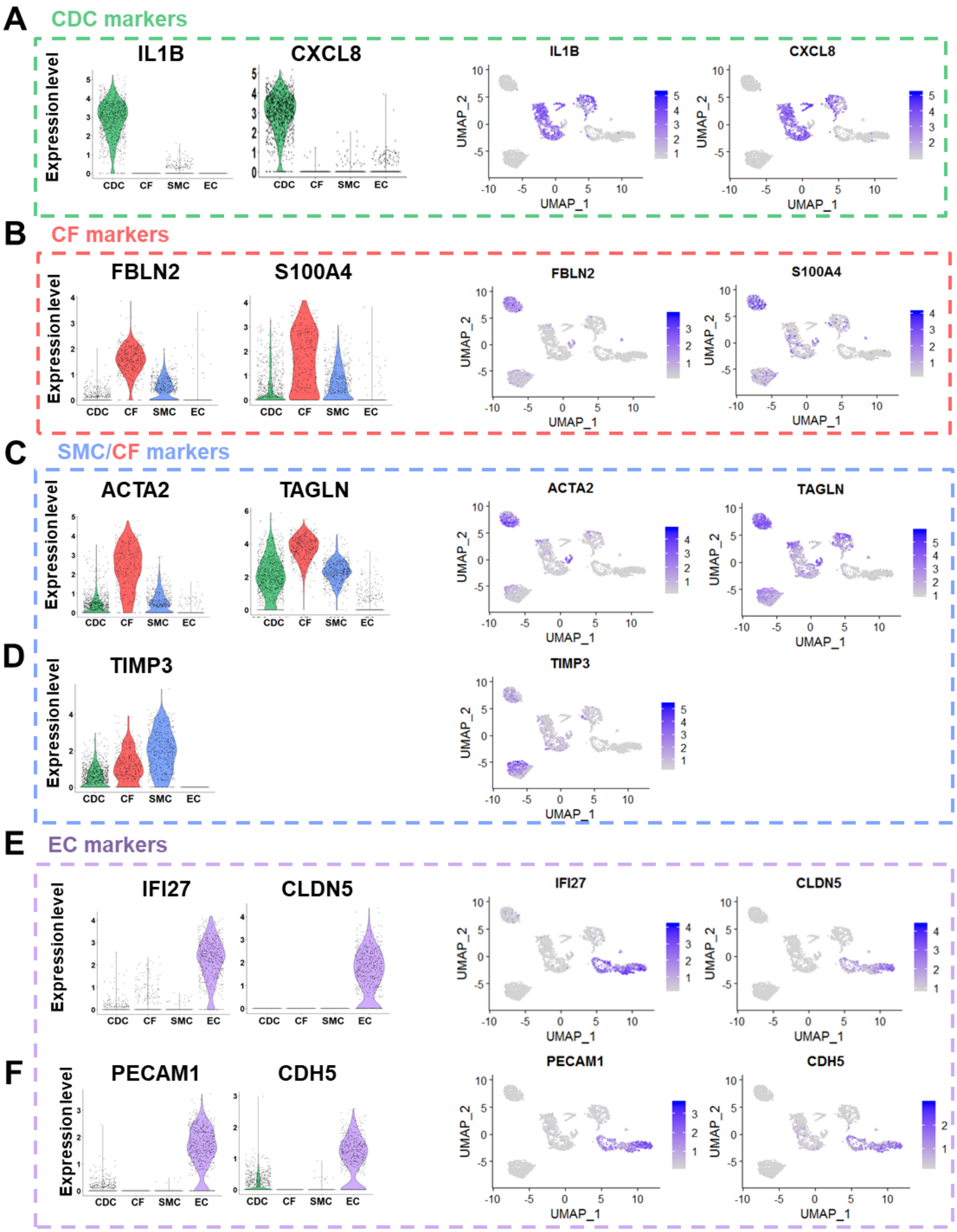

Supplemental Figure S3: Upregulated differentially expressed genes (uDEGs) in CDCs, CFs, SMCs and ECs. Violin plots and UMAP plots generated from sc-RNAseq data for uDEGs in the **A)** CDC sample (IL1B, CXCL8), **B-C)** CF sample (FBLN2, S100A4, ACTA2, TAGLN). **D)** SMC sample (TIMP3) and **E-F)** EC sample (IFI27, CLDN5, PECAM1, CDH5).

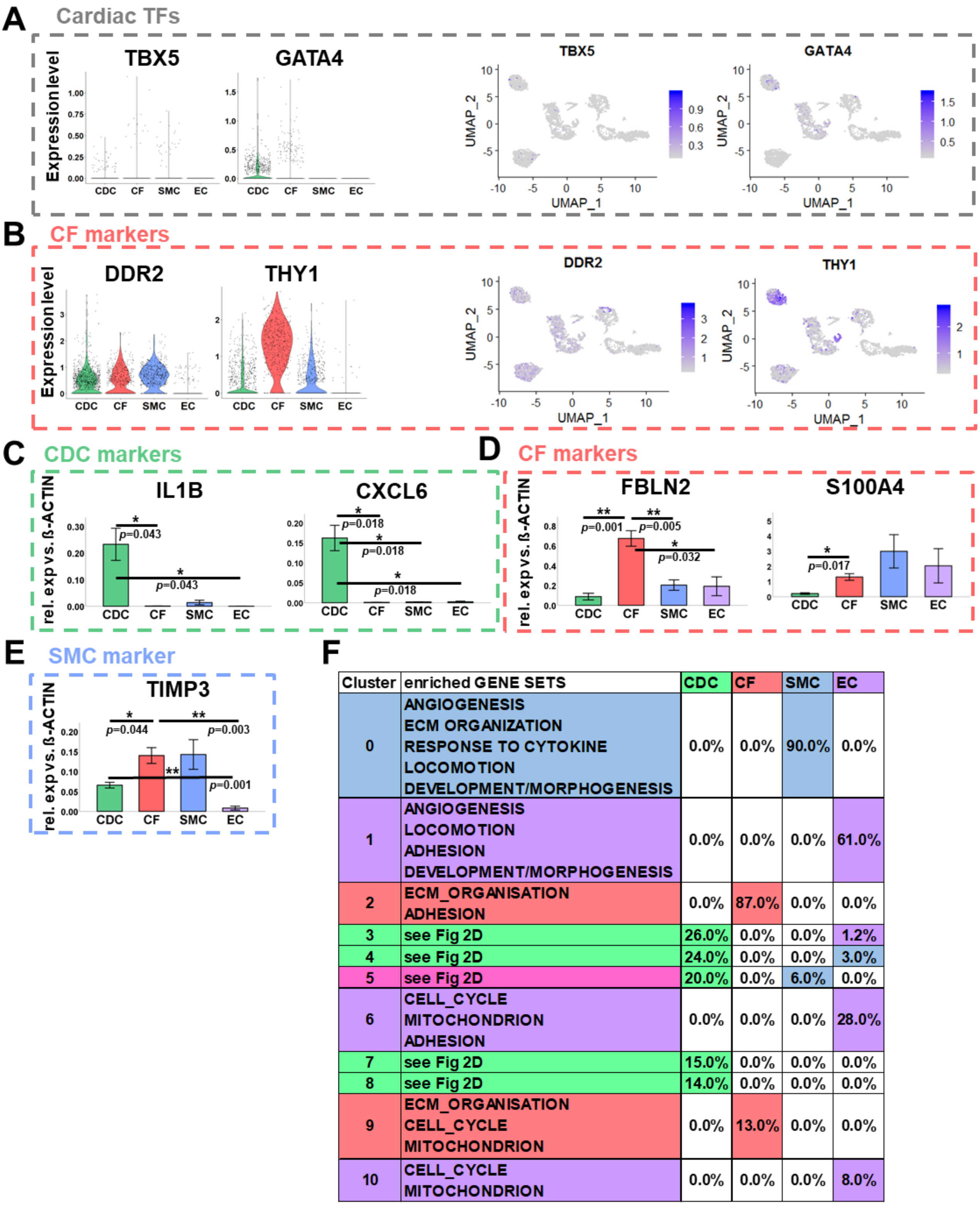

F

Cluster	enriched GENE SETS	CDC	CF	SMC	EC
0	ANGIOGENESIS<br>ECM ORGANIZATION<br>RESPONSE TO CYTOKINE<br>LOCOMOTION<br>DEVELOPMENT/MORPHOGENESIS	0.0%	0.0%	90.0%	0.0%
1	ANGIOGENESIS<br>LOCOMOTION<br>ADHESION<br>DEVELOPMENT/MORPHOGENESIS	0.0%	0.0%	0.0%	61.0%
2	ECM_ORGANISATION<br>ADHESION	0.0%	87.0%	0.0%	0.0%
3	see Fig 2D	26.0%	0.0%	0.0%	1.2%
4	see Fig 2D	24.0%	0.0%	0.0%	3.0%
5	see Fig 2D	20.0%	0.0%	6.0%	0.0%
6	CELL_CYCLE<br>MITOCHONDRION<br>ADHESION	0.0%	0.0%	0.0%	28.0%
7	see Fig 2D	15.0%	0.0%	0.0%	0.0%
8	see Fig 2D	14.0%	0.0%	0.0%	0.0%
9	ECM_ORGANISATION<br>CELL_CYCLE<br>MITOCHONDRION	0.0%	13.0%	0.0%	0.0%
10	CELL_CYCLE<br>MITOCHONDRION	0.0%	0.0%	0.0%	8.0%

<u>Supplemental Figure S4: Confirmation of selected gene expression detected by sc-RNAseq by qRT PCR</u>. A-B) Violin and UMAP plots generated from sc-RNAseq data of marker genes used for

molecular characterization before (Fig 1 and Suppl Fig S1). **A)** Cardiac transcription factors (TFs) (TBX5, GATA4) and **B)** cardiac fibroblast (CF) markers (DDR2, THY1). **C-E)** Confirmation of selected gene expression detected by sc-RNAseq (uDEGs) by qRT-PCR in independent samples. **C)** CDC markers (IL1B, CXCL6), **D)** CF markers (FBLN2, S100A4) and **E)** SMC marker (TIMP3).

F) Enriched Gene Set topics for the main clusters in Fig 2C. Find the complete Table as Suppl. Table 8.

Bar diagrams show means +/- SEM. *: $p < 0.05$, **: $p < 0.01$.

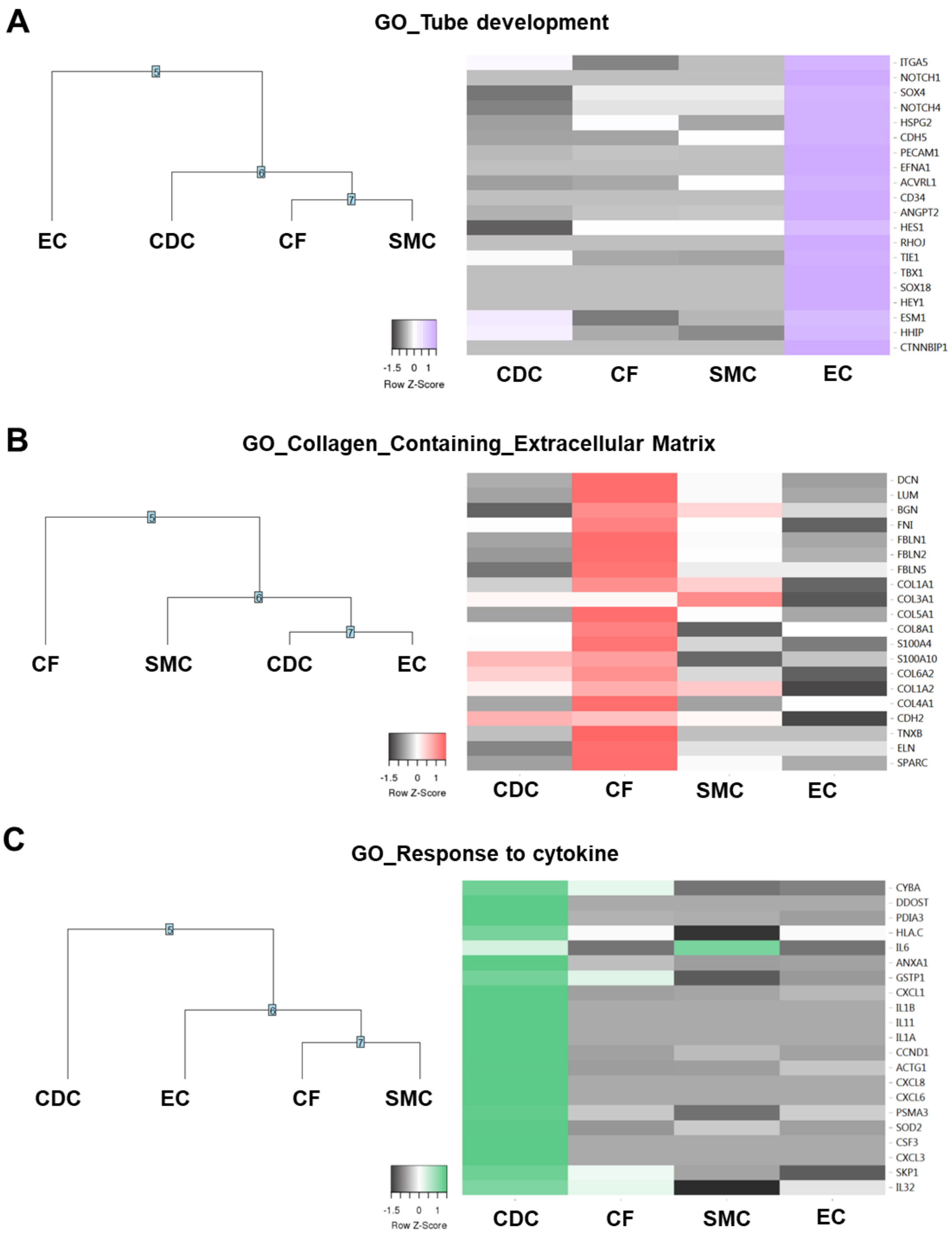

<u>Supplemental Figure S5: Cluster trees and heat maps from sc-RNAseq data for CDCs, CFs, SMCs and ECs.</u> A) UDEGs associated with the GO term “Tube development” in cluster 1 were analyzed in a cluster tree (left panel) and exemplary uDEGs are shown in a heat map (right panel). Analogical analysis was performed for uDEGs associated with the GO term “Collagen Containing Extracellular Matrix” in cluster 2 **(B)** and uDEGs associated with the GO term “Response to cytokines” in cluster 3 **(C).**

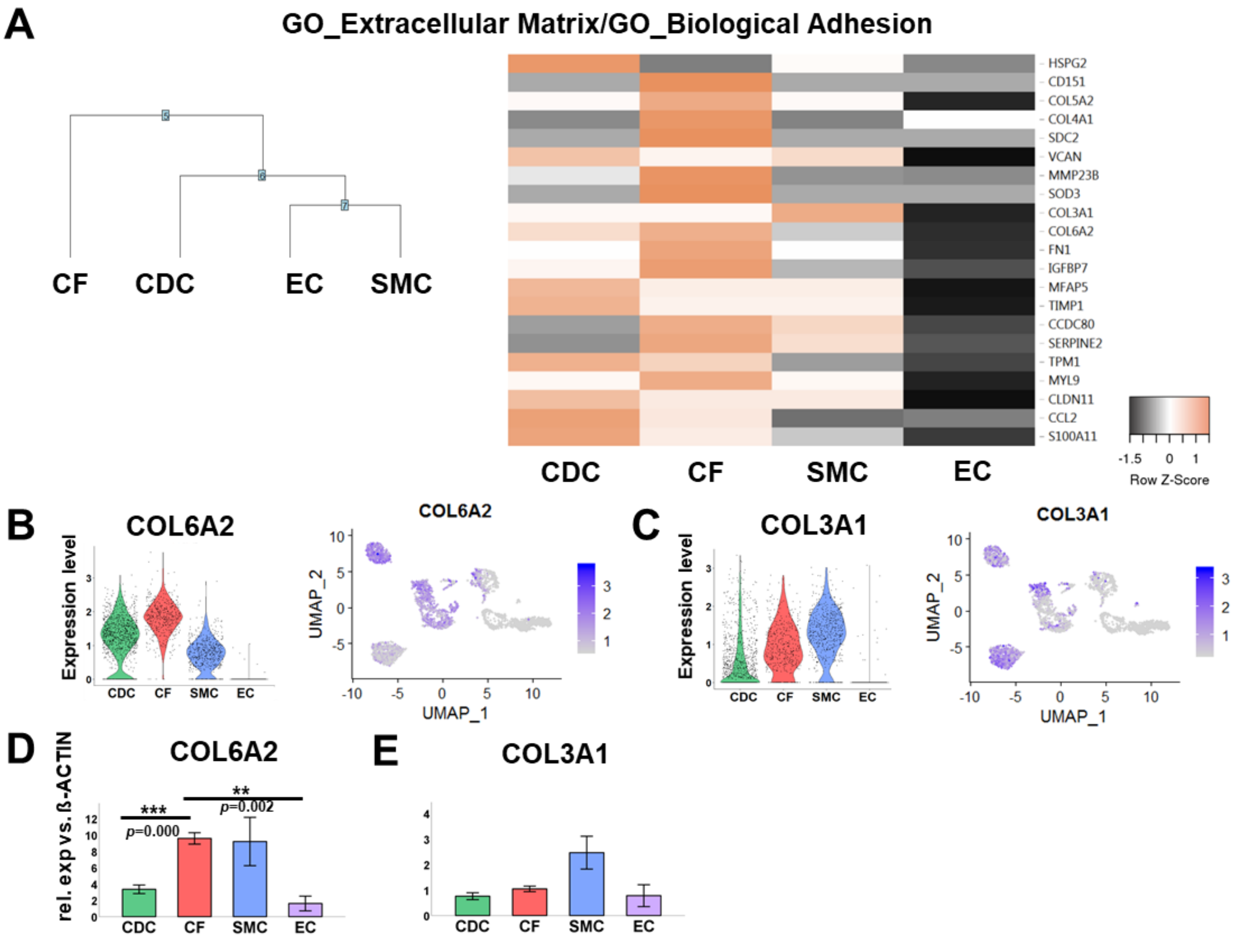

F

Cluster	enriched GO-terms		CDC	DIFF D6	DIFF D7	DIFF D8	DIFF D9	DIFF D15
0	CELL_CYCLE	n cells	233	40	20	22	8	10
	CELL_DIVISION	% cells	24.81%	37.38%	18.52%	18.80%	6.96%	9.90%
1	CELL_MIGRATION	n cells	292	3	4	4	2	1
	ANCHORING_JUNCTION	% cells	31.10%	2.80%	3.70%	3.42%	1.74%	0.99%
2	ECM_ORGANISATION	n cells	165	26	34	18	18	11
	EXTERNAL_ENCAPSULATING_STRUCTURE	% cells	17.57%	24.30%	31.48%	15.38%	15.65%	10.89%
3	HEART_DEVELOPMENT	n cells	0	13	33	51	70	62
	CARDIAC_MUSCLE_CONTRACTION	% cells	0.00%	12.15%	30.56%	43.59%	60.87%	61.39%
4	TRANSLATION_ELONGATION	n cells	227	0	0	0	0	0
	RIBOSOME	% cells	24.17%	0.00%	0.00%	0.00%	0.00%	0.00%
5	BIOLOGICAL_ADHESION	n cells	0	16	5	7	8	14
	EPITHELIUM_DEVELOPMENT	% cells	0.00%	14.95%	4.63%	5.98%	6.96%	13.86%
6	DNA_REPLICATION	n cells	22	8	6	5	0	2
	CELL_CYCLE	% cells	2.34%	7.48%	5.56%	4.27%	0.00%	1.98%
7	LOCOMOTION	n cells	0	1	6	10	9	1
	CIRCULATORY_SYSTEM_DEVELOPMENT	% cells	0.00%	0.93%	5.56%	8.55%	7.83%	0.99%
all cells			939	107	108	117	115	101
< 1% of cells - no colour								

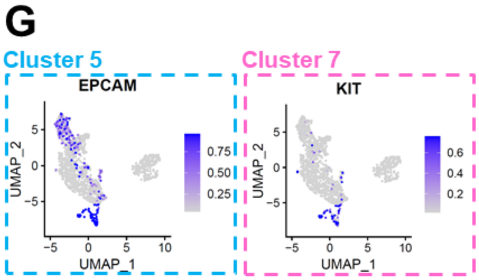

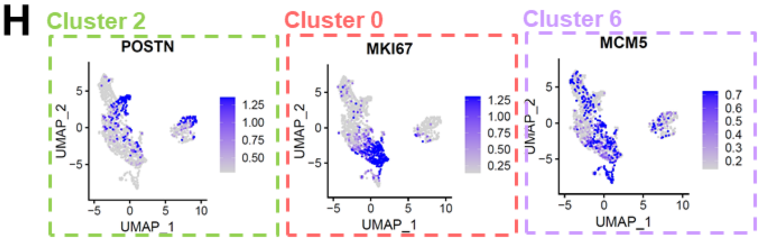

Supplemental Figure S6: Similarities between CDCs, CFs and SMCs and sc-RNAseq data of adult CDCs integrated with sc-RNAseq data of differentiating hESCs (DIFF D6-DIFF D15). A) Similarly

upregulated genes in CDCs, CFs and SMCs (sc-RNAseq data) were associated with the terms "extracellular matrix" and "biological adhesion", as shown in a cluster tree (left panel) and as illustrated in a heat map of a selected gene subset (right panel). **B-C)** Violin and UMAP plots generated from sc-RNAseq data showing expression levels of *COL6A2* **(B)** and *COL3A1* **(C)** in CDCs, CFs, SMCs and ECs. **D-E)** Relative RNA expression versus β-ACTIN assessed by qRT-PCR of *COL6A2* **(D)** and *COL3A1* **(E)** in CDCs, CFs, SMCs and ECs (independent samples). Bar diagrams show means +/- SEM. **: $p < 0.01$, ***: $p < 0.001$. **F)** Table shows enriched GO-Terms (detected by GSEA) for clusters depicted in Fig 2G. The number of cells (n cells) from each sample (and the percentage, % cells) detected in each cluster are also shown. **G)** UMAP plots generated from sc-RNAseq data showing gene expression of *EPCAM* (epithelial progenitor marker) and *KIT* (angiogenic progenitor marker). **H)** UMAP plots generated from sc-RNAseq data showing gene expression of *POSTN* (activated CF marker), *MKI67* (proliferation marker) and *MCM5* (proliferation marker).

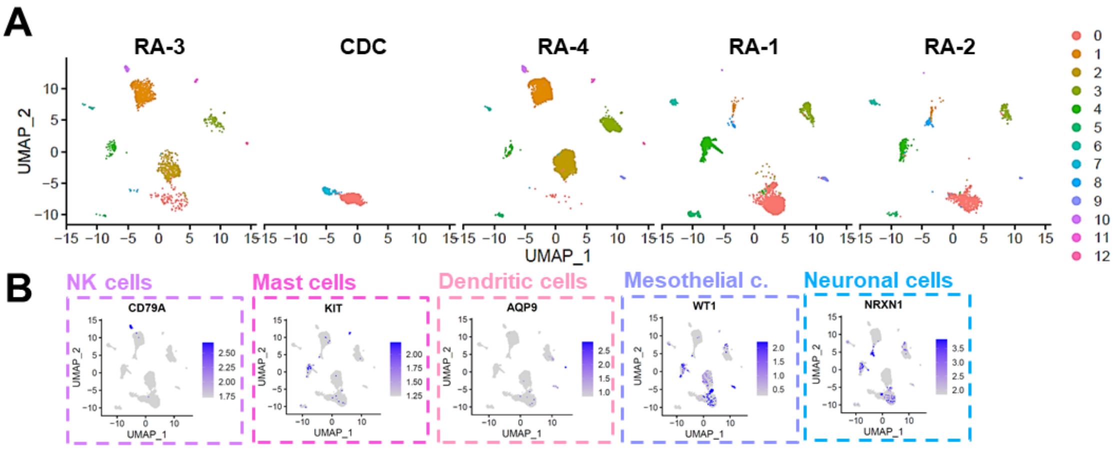

C

Cluster	cell type		CDC	RA-1	RA-2	RA-3	RA-4
0	CF (SN), CDC	n cells	721	2074	542	114	27
		% cells	76.78%	51.11%	47.25%	7.96%	0.61%
1	B/T-cells	n cells	0	51	44	619	1658
		% cells	0.00%	1.26%	3.84%	43.23%	37.44%
2	CF (SC)	n cells	0	17	2	391	1612
		% cells	0.00%	0.42%	0.17%	27.30%	36.40%
3	MP	n cells	0	372	127	94	696
		% cells	0.00%	9.17%	11.07%	6.56%	15.72%
4	EC	n cells	0	887	216	85	58
		% cells	0.00%	21.86%	18.83%	5.94%	1.31%
5	SMC/PC	n cells	0	277	22	10	58
		% cells	0.00%	6.83%	1.92%	0.70%	1.31%
6	CM	n cells	0	185	54	18	89
		% cells	0.00%	4.56%	4.71%	1.26%	2.01%
7	CDC	n cells	216	0	0	7	4
		% cells	23.00%	0.00%	0.00%	0.49%	0.09%
8	Neuronal Cells	n cells	2	65	132	1	0
		% cells	0.21%	1.60%	11.51%	0.07%	0.00%
9	Mesothelial cells	n cells	0	128	7	0	41
		% cells	0.00%	3.15%	0.61%	0.00%	0.93%
10	NK cells	n cells	0	2	1	40	107
		% cells	0.00%	0.05%	0.09%	2.79%	2.42%
11	Mast cells	n cells	0	0	0	27	62
		% cells	0.00%	0.00%	0.00%	1.89%	1.40%
12	Dendritic cells	n cells	0	0	0	26	16
		% cells	0.00%	0.00%	0.00%	1.82%	0.36%
all cells			939	4058	1147	1432	4428
<0.5% of cells - no colour							

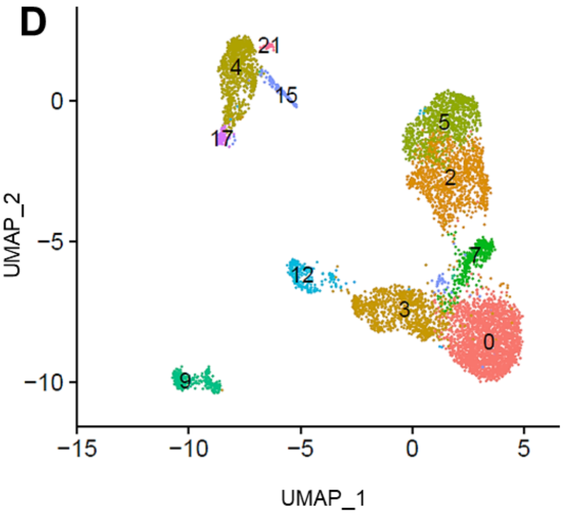

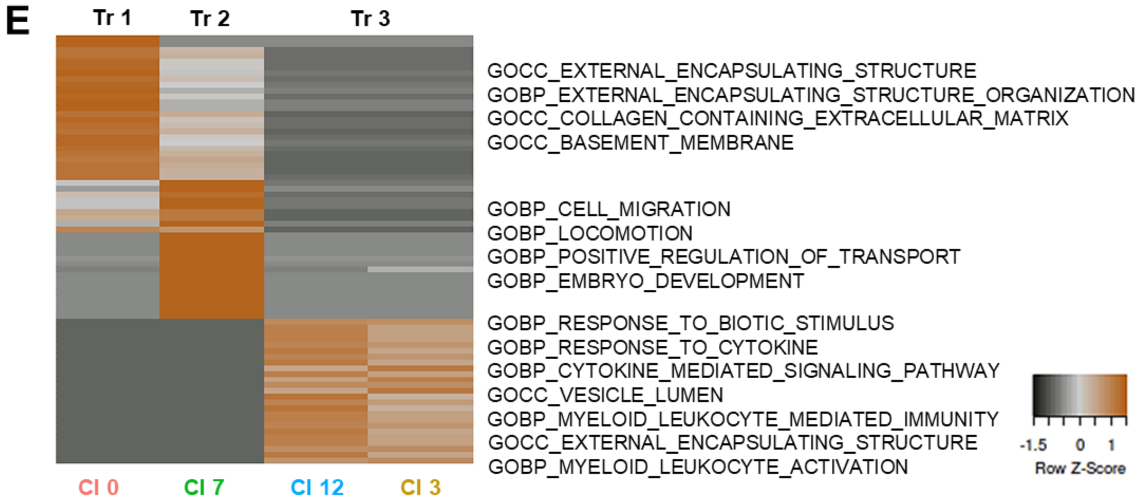

Supplemental Figure S7: Sc-RNAseq data of CDCs integrated with sc- and sn-RNAseq data of human atrial biopsies. **A)** Splitted view of the UMAP plot in Fig 3B. Colors indicate the 13

identified clusters in Seurat. RA, right atrium; CDC, cardiosphere derived cells. **B)** UMAP plots with gene expression for markers of natural killer (NK) cells (*CD79A*), mast cells (*KIT*), dendritic cells (*AQP9*), mesothelial cells (*WT1*) and neuronal cells (*NRXN1*). **C)** Table that indicates the cell identity of each cluster including the number (n cells) and percentage (% cells) of each sample for each cluster. SN, single-nucleus RNA sequencing; SC, single-cell RNA sequencing. **D)** Zoomed view of Fig 3B with a more detailed subclustering fitting to the trajectory branches in Fig 3D. **E)** Heat Map of the Top25 specific genes of cluster 0 (Cl 0), cluster 7 (Cl 7), and cluster 12 (Cl 12) mapping to the three trajectories (tr 1, tr 2, tr 3). Cluster 3 was included in the heat map to show that cluster 12 specific genes are also upregulated in cluster 3. Typical upregulated GO terms (detected by GSEA) for each cluster/trajectory were included.

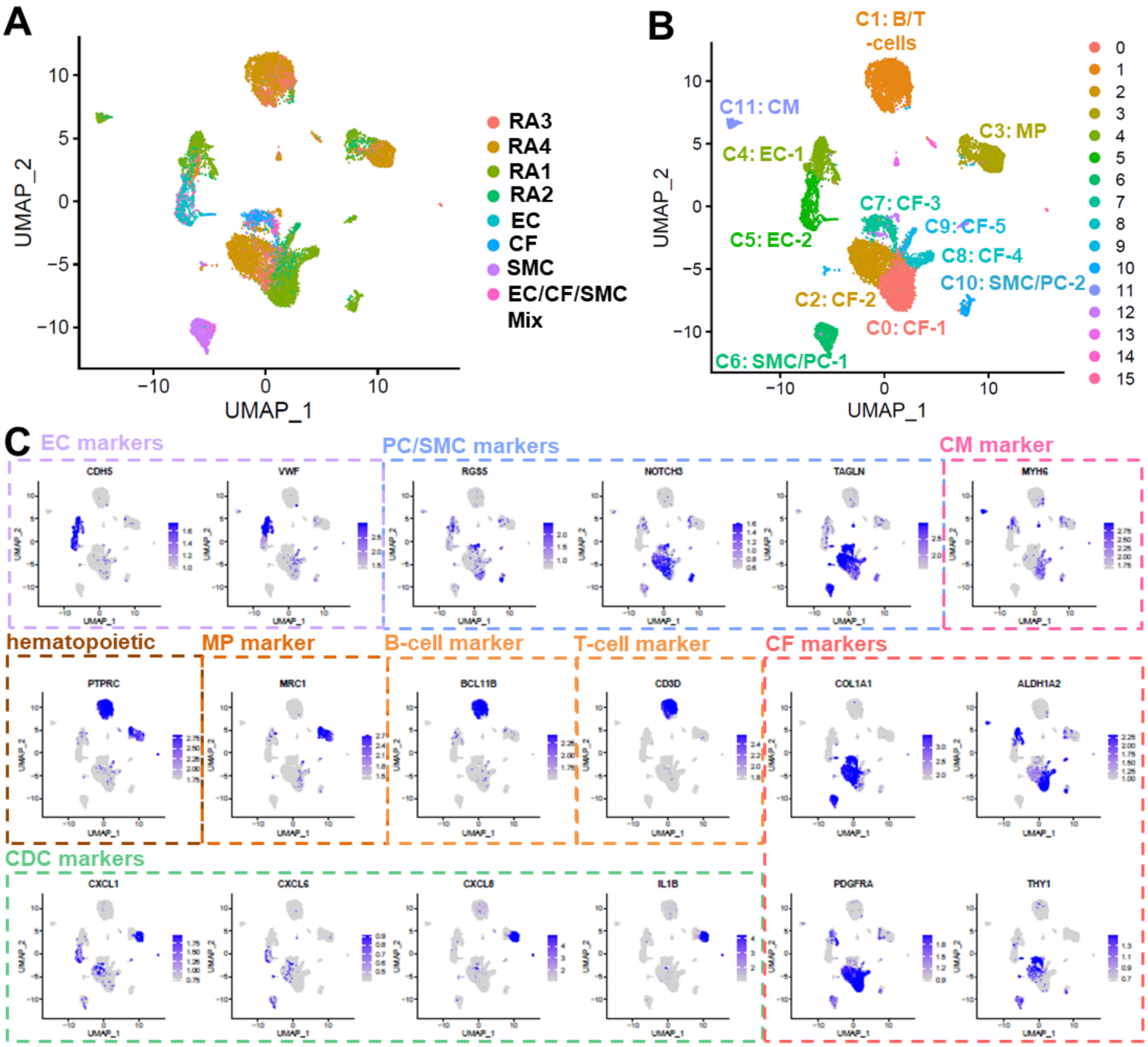

D

	Cl	0	2	7	8	9	4	5	6	10	11	3	1	12	13	14	15	all cells
	cell type	CF-1	CF-2	CF-3	CF-4	CF-5	EC-1	EC-2	SMC/PC-1	SMC/PC-2	CM	MP	B/T-cells					
RA1	n cells	1732	17	9	330	70	882	16	NA	268	185	369	48	130	NA	2	NA	4058
	% cells	42.68	0.42	0.22	8.13	1.72	21.73	0.39	-	6.60	4.56	9.09	1.18	3.20	-	0.05	-	100
RA2	n cells	427	16	13	52	183	172	46	2	20	53	127	24	8	NA	4	NA	1147
	% cells	37.23	1.39	1.13	4.53	15.95	15.00	4.01	0.17	1.74	4.62	11.07	2.09	0.70	-	0.35	-	100
RA3	n cells	300	177	5	15	11	61	30	1	1	1	87	661	12	16	28	26	1432
	% cells	20.95	12.36	0.35	1.05	0.77	4.26	2.09	0.07	0.07	0.07	6.08	46.16	0.84	1.12	1.96	1.82	100
RA4	n cells	60	1527	53	NA	15	44	17	NA	NA	NA	687	1770	89	90	60	16	4428
	% cells	1.36	34.49	1.20	-	0.34	0.99	0.38	-	-	-	15.51	39.97	2.01	2.03	1.36	0.36	100
CF	n cells	4	13	386	NA	NA	NA	26	NA	NA	NA	NA	NA	NA	NA	NA	NA	429
	% cells	0.93	3.03	89.98	-	-	-	6.06	-	-	-	-	-	-	-	-	-	100
EC	n cells	NA	NA	9	NA	6	NA	630	NA	NA	NA	NA	1	NA	NA	NA	NA	646
	% cells	-	-	1.39	-	0.93	-	97.52	-	-	-	-	0.15	-	-	-	-	100
SMC	n cells	NA	NA	NA	NA	33	NA	NA	534	NA	NA	NA	NA	NA	2	NA	NA	569
	% cells	-	-	-	-	5.80	-	-	93.85	-	-	-	-	-	0.35	-	-	100
Mix	n cells	12	7	170	NA	8	NA	125	143	NA	NA	NA	NA	NA	NA	NA	NA	465
	% cells	2.58	1.51	36.56	-	1.72	-	26.88	30.75	-	-	-	-	-	-	-	-	100
<1% of cells - no colour																		

<u>Supplemental Figure S8: Sc-RNAseq data of cultivated CFs, ECs and SMCs integrated with sc- and sn-RNAseq data of human atrial biopsies.</u> A) UMAP plot of cultivated CFs (CF), ECs (EC) and SMCs (SMC) sc-RNAseq data integrated with sn/sc-RNAseq data from four human right atrial

biopsies. Color indicates sample identifier. RA, right atrium; CF, cardiac fibroblasts, EC, endothelial cells, SMC, smooth muscle cells. **B)** UMAP plot of cultivated CF, EC and SMC sc-RNAseq data integrated with sn/sc-RNAseq data from four human right atrial biopsies. Color indicates cluster identity. Unsupervised clustering revealed 16 clusters identifying all main cell types of the human heart (see also C). Abbreviations: CM, cardiomyocytes; MP, macrophages; PC, pericytes. **C)** UMAP plots showing gene expression levels of various markers defining cell type identity of the clusters. **D)** Table that indicates the cell identity of each main cluster including the number (n cells) and percentage (% cells) of each sample for each cluster.

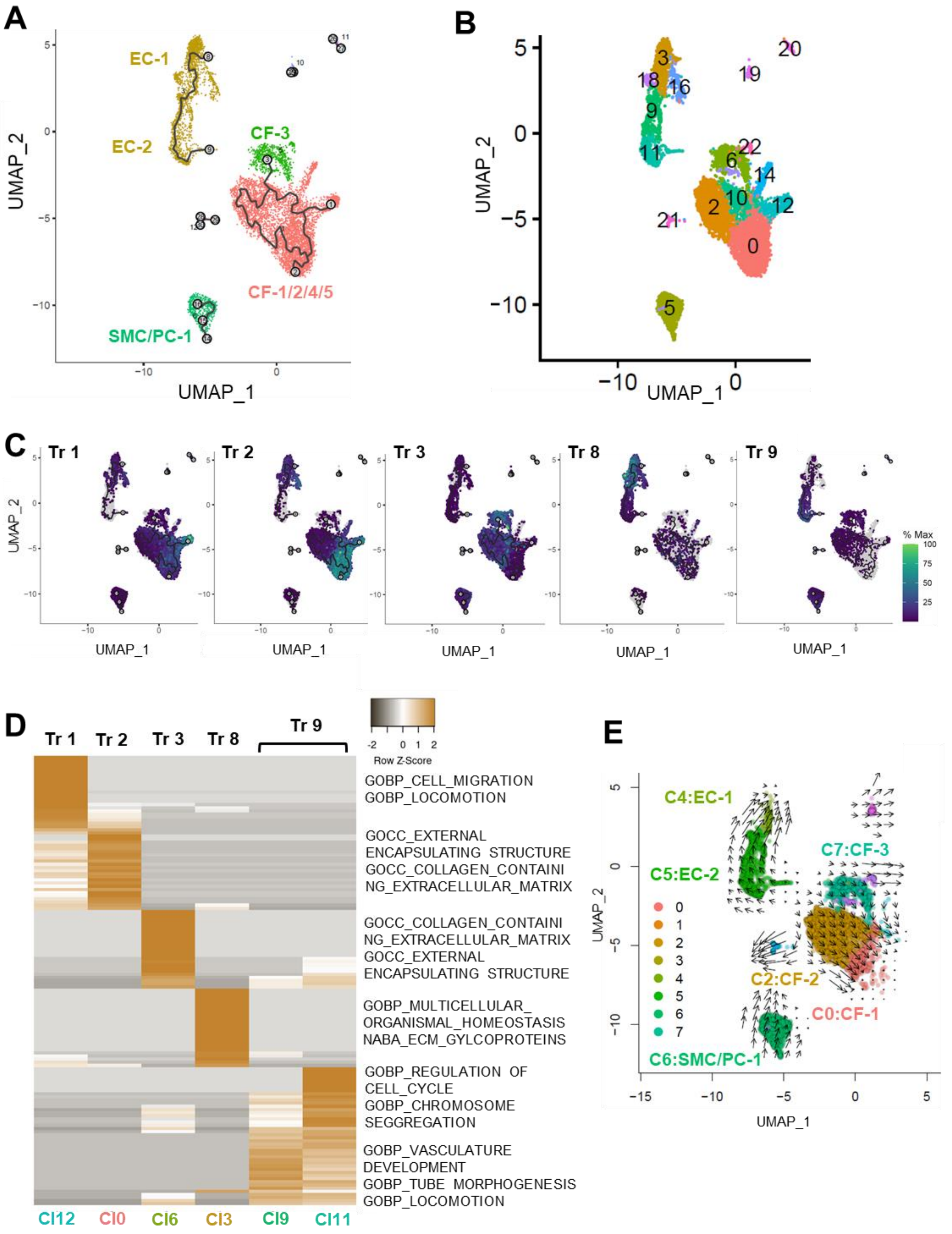

Supplemental Figure S9: Comparison of cultivated CFs, ECs, SMCs and human atrial biopsies by sc-/sn-RNAseq. A) Zoomed view of trajectories detected in CF and EC clusters (Tr 1, 2, 3 – CF

tranjectories; Tr 8, 9 – EC trajectories). **B)** Zoomed view of Suppl Fig 8B with a more detailed subclustering fitting to the trajectory branches in Suppl Fig S9A. **C)** Overlapping gene expression of 4 to 5 top specific genes for each trajectory (Tr1: *WT1, TBX18, NR4A1, TBX20*; Tr2: *COL4A4, LAMA2, RORA, TBX20*; Tr3: *TAGLN, ACTA2, COL1A1, FN1*; Tr8: *VWF, FLT1, PECAM1, POSTN*; Tr9: *CLDN5, SERPINE1, IFI27, MT2A, MKI67*). **D)** Heat Map of the Top25 specific genes of cluster 0 (Cl 0), cluster 3 (Cl 3), cluster 6 (Cl 6), cluster 9 (Cl 9), cluster 11 (Cl 11) and cluster 12 (Cl 12) mapping to the five trajectories (Tr 1, Tr 2, Tr 3, Tr 8, Tr 9). Cluster 9 was included in the heat map to show that cluster 11 specific genes are also upregulated in cluster 9. Typical upregulated GO terms (detected by GSEA) for each cluster/trajectory were included. **E)** RNA velocity analysis performed by Velocyto. Velocity field projected onto the UMAP plot. Zoomed view of EC, SMC/PC, and CF clusters according to zoomed view in A and B. Color indicates cluster identity (see also Suppl Fig S8B). Arrows show the local average velocity and point from the CDC-2 cluster to the CDC-1/CF-1 cluster.

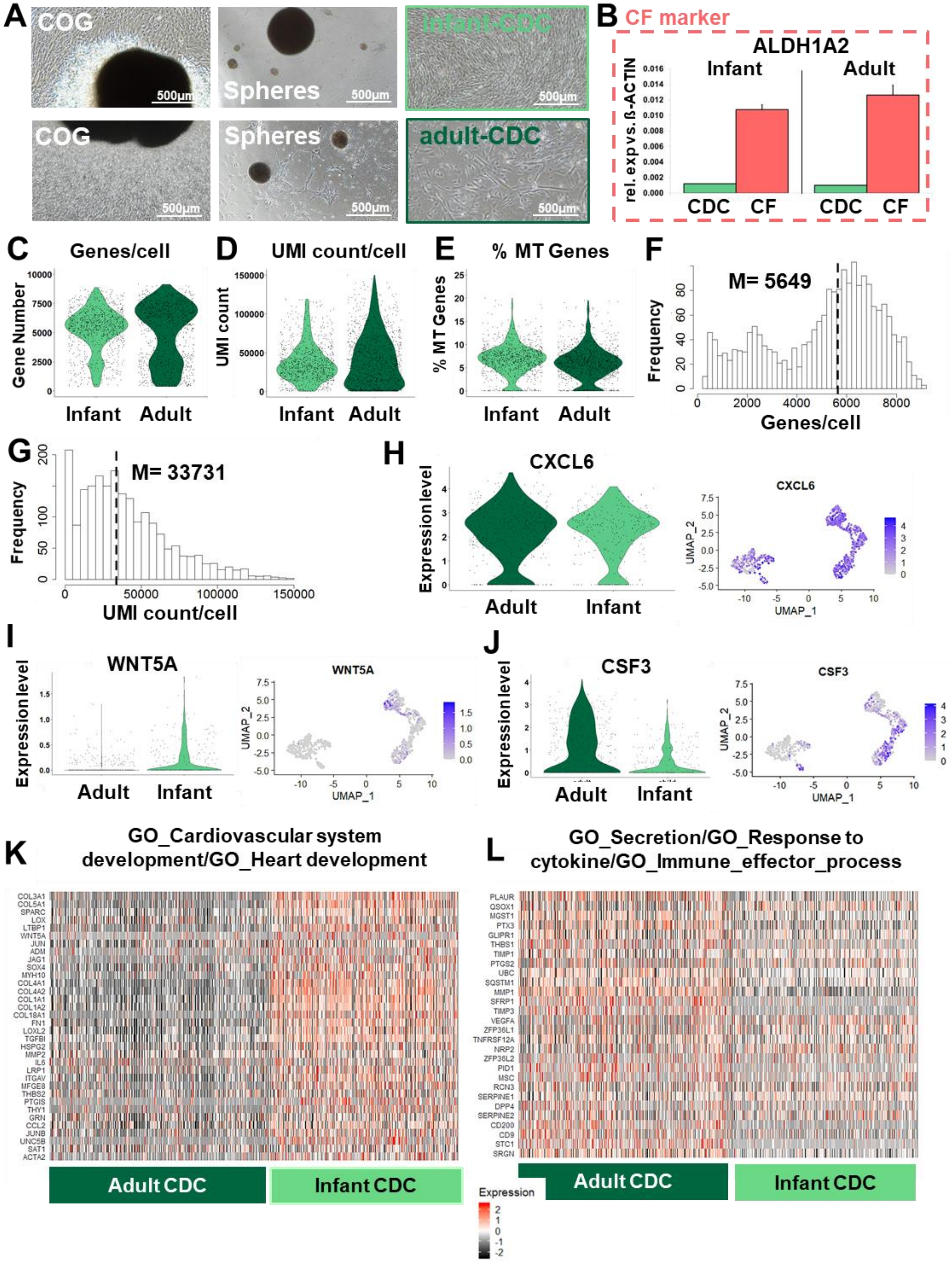

Supplemental Figure S10: Quality control of infant and adult CDC samples used for sc-RNAseq and comparison between sc-RNAseq data of adult and infant CDCs. **A)** Morphological

assessment of the utilized cell samples (infant and adult CDCs) by phase contrast microscopy directly before the sc-RNAseq experiment (right panels). COG, cardiac outgrowth **B)** Gene expression profile (relative RNA expression versus β-ACTIN) of specific CF marker *ALDH1A2* of the infant and the adult CDC sample used for sc-RNAseq compared to CFs generated from the same patient's cardiac biopsy. Bar diagrams show means +/- SEM. **C-E)** Violin plots of detected gene number per cell (Genes/cell) (**C**), unique molecular identifier count per cell (UMI count/cell) (**D**) and percentage of mitochondrial genes per cell (% MT Genes) (**E**), illustrated for the infant and adult CDC sample after the filtering procedure (median values in Suppl Table 7). **F-G)** Histogram illustrating the frequency of genes/cell (**F**) or the frequency of UMI count/cell (**G**) of the filtered merged Seurat object containing infant and adult CDCs. Abbreviations: M median. **H-J)** Violin and UMAP plots generated from sc-RNAseq data for the CDC-specific marker *CXCL6* (**H**), the cardiac development associated marker *WNT5A* upregulated in infant CDCs (cluster 1) (**I**) and the cytokine *CSF3* upregulated in adult CDCs, mainly in cluster 2 (**J**). **K-L)** Heat maps of selected uDEGs for the infant CDC sample **(K)** and for the adult CDC sample **(L)**. Enriched Gene set topics for the selected genes are displayed above the according heat maps.

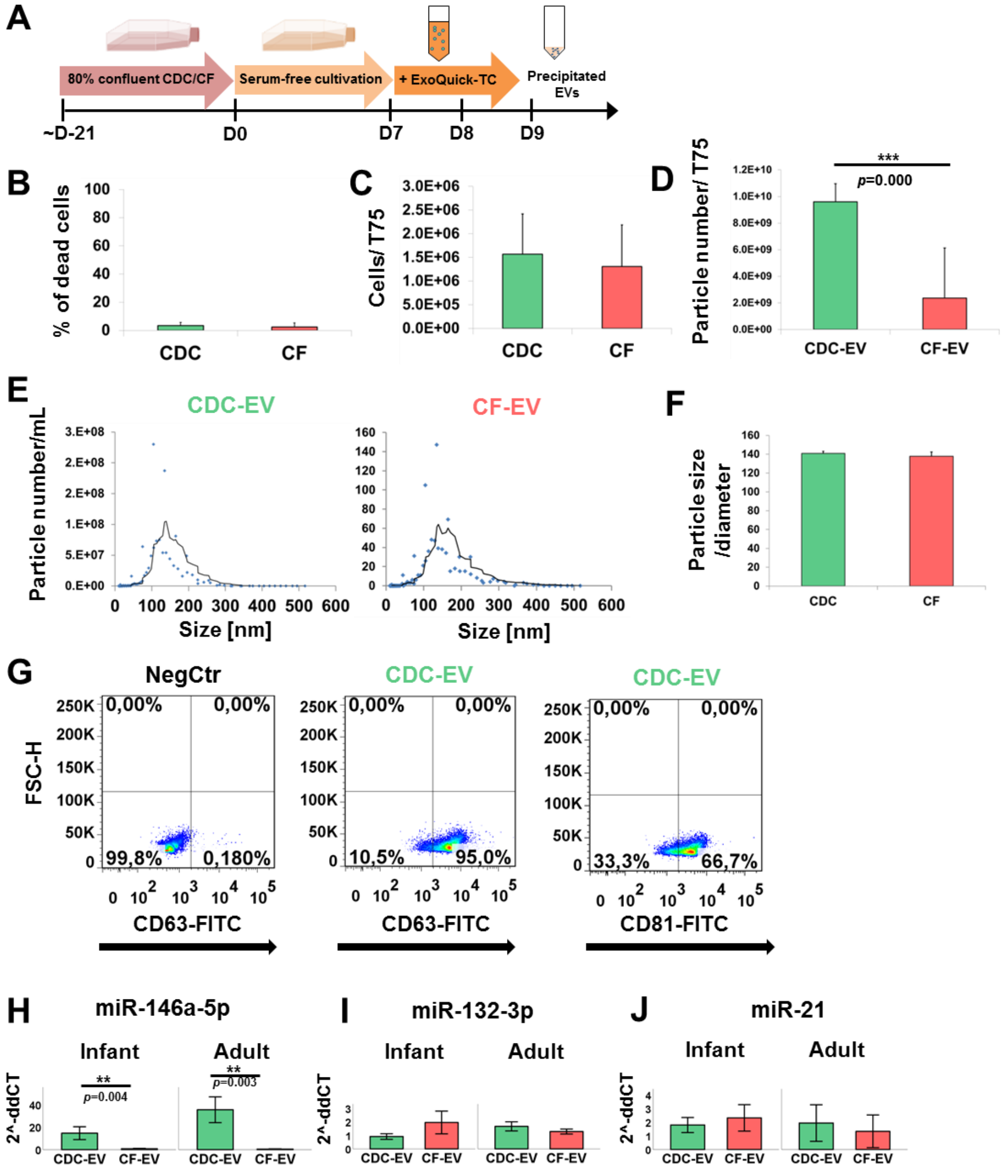

<u>Supplemental Figure S11: Characterization of extracellular vesicles (EVs) of CDCs and CFs</u>. A) Isolation procedure of EVs (with ExoQuick-TC solution) from conditioned medium of CDCs and CFs cultured in serum-free medium for seven days. **B)** Percentage of dead cells per T75 cell culture flask after conditioning CDCs/CFs with serum-free media for seven days (evaluation of dead cells by trypan blue staining). **C)** Cell number per T75 cell culture flask after conditioning CDCs/CFs with serum-free media for seven days. **D)** EV yield measured as particle number per T75 cell culture flask is significantly higher in CDCs compared to CFs. **E)** Exemplary and representative plots of the size distribution of particles in a CDC and a CF-EV preparation. **F)** Size distribution of particles (diameter in nm) of CDC- and CF-EVs. **G)** Flow cytometry analysis of exosomal markers CD63 and CD81 in CDC-EV preparations (exemplary plots). **H-J)** MicroRNA expression in infant/adult CDC-/CF-EVs, data are shown as means +/- SEM. ** *p* < 0.01. Parts of the figure were created with Biorender.com.

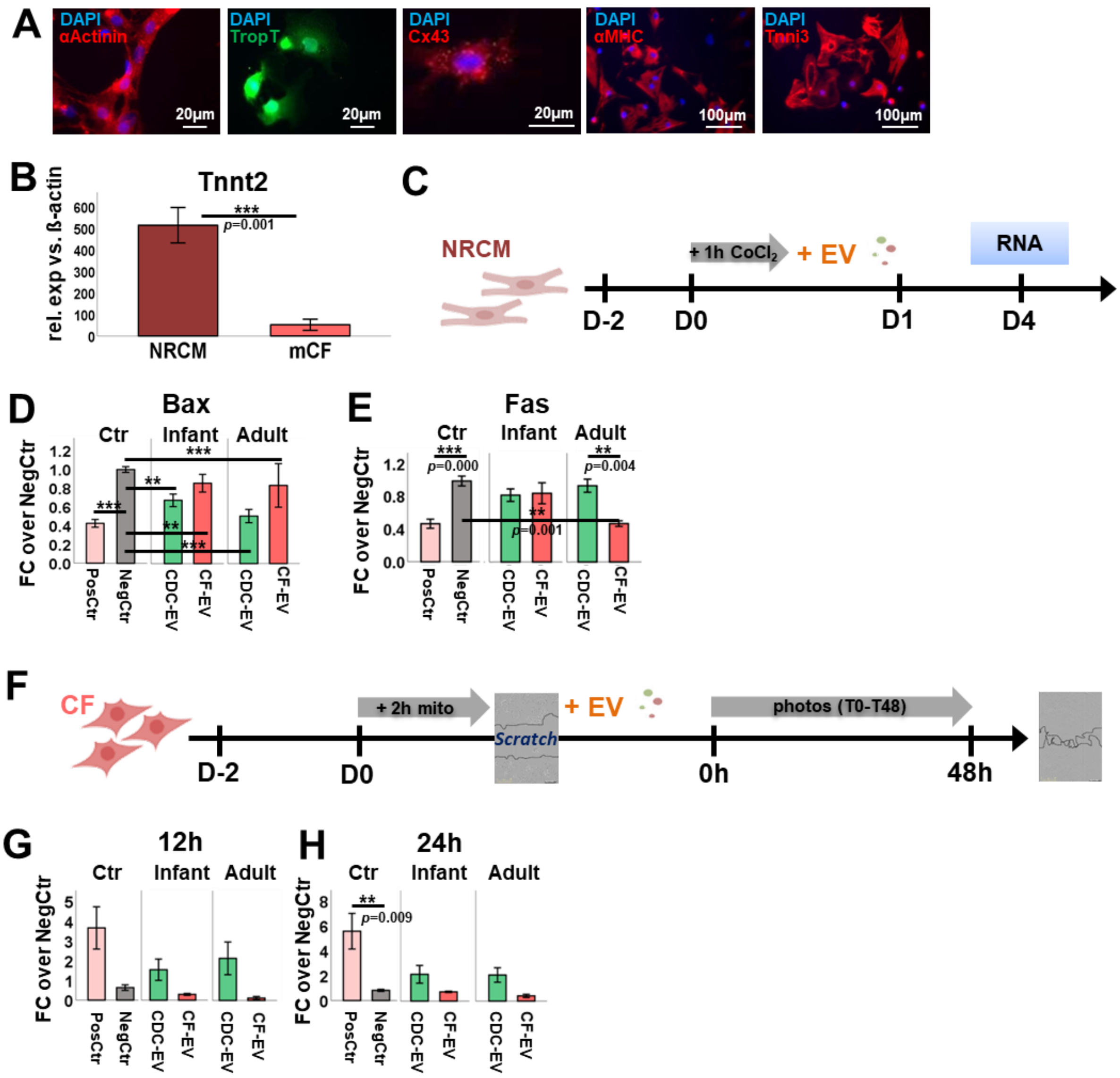

<u>Supplemental Figure S12: Functional *in vitro* assays with EVs and NRCM characterization</u>. A) Immunocytochemical staining against the cardiomyocyte markers α-Actinin, TropT, Cx43, αMHC and Tnni3 in neonatal rat cardiomyocytes (NRCMs). **B)** Gene expression analysis of *Tnnt2 (TropT)* in NRCMs compared to murine cardiac fibroblasts (mCFs). **C-E)** Apoptosis test with NRCMs: Experimental outline (**C**). Gene expression analysis (apoptosis marker Bax (**D**) and Fas (**E**)) by qRT-PCR in treated NRCMs. Relative RNA expression to β-ACTIN was normalized to the negative control (FC over NegCtr). **F-H)** Migration assays (scratch assays) with human CFs: Experimental outline (**F**). Comparison of the differences of the cell-free area between the time point 0h and 12h **(G)** or 24h **(H)** normalized to the negative control (FC over NegCtr). Data are represented as mean +/- SEM, * $p < 0.05$, ** $p < 0.01$, *** $p < 0.001$. Parts of the figure were created with Biorender.com.

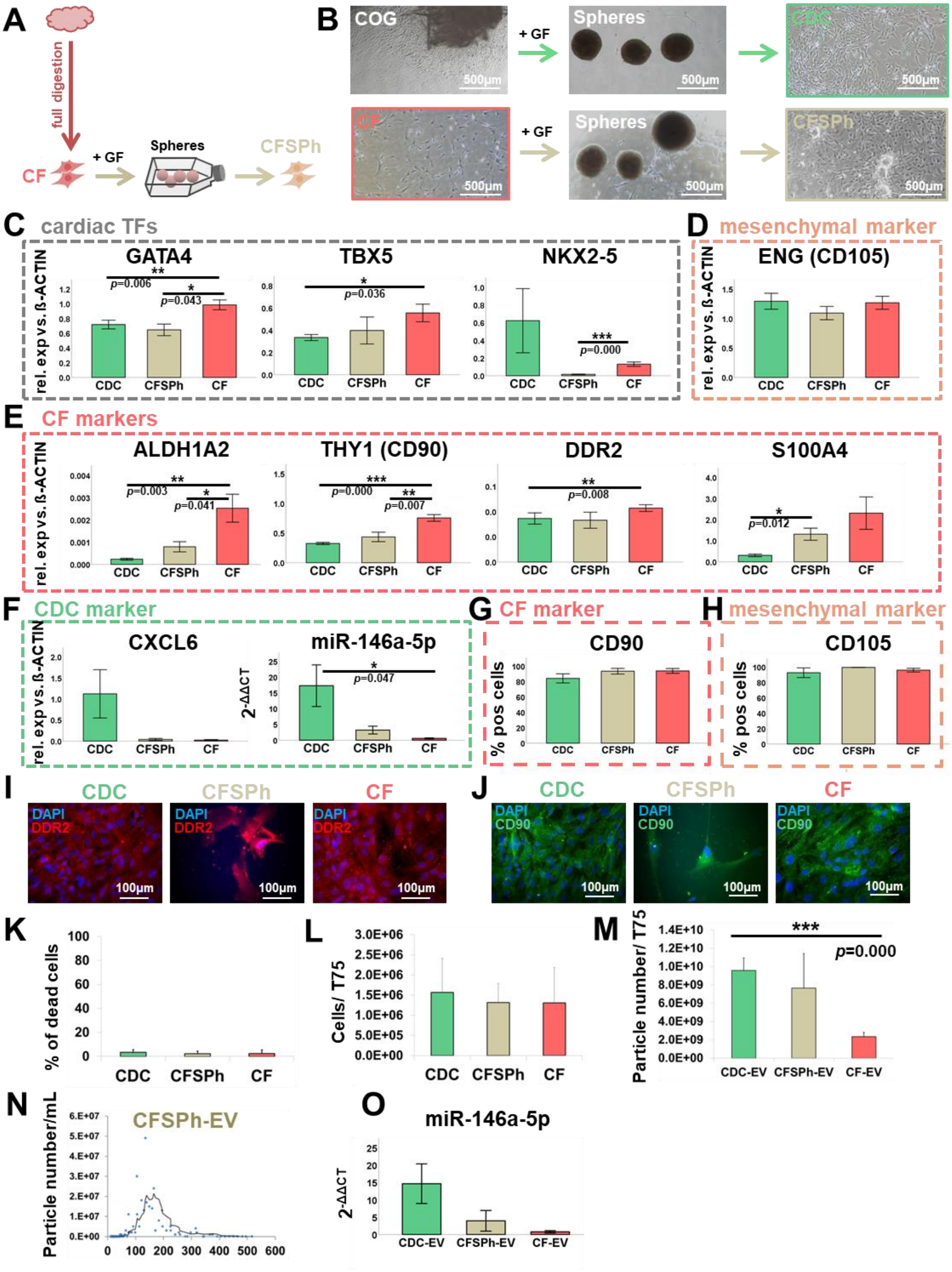

Supplemental Figure S13: Sphere formation as a prerequisite for regenerative characteristics - characterization of neonatal CFSPhs compared to CDCs and CFs of the same age. All samples

included were derived from neonatal patients (age ≤ 21 days). **A)** Generation of CFSPhs from cardiac fibroblasts of neonatal patients. GF growth factors. **B)** Representative pictures of the three cultivation stages of CDCs (top) and CFSPhs (bottom). COG cardiac outgrowth. **C-F)** Gene expression and miR expression analysis (by qRT-PCR) of cardiac TFs (**C**), the mesenchymal marker *ENG* (*CD105)* (**D**), CF markers (**E**), and CDC markers (**F**). **G-H)** Flow cytometry analysis of CD90 **(G)** and CD105 **(H)** revealed ubiquitous abundance in neonatal CDCs, CFSPhs and CFs. **I-J)** Immunocytochemical staining against DDR2 and CD90 in neonatal CDCs, CFSPhs and CFs also showed ubiquitous abundance. **G-H)** Percentage of dead cells **(G)** and cell number per T75 culture flask **(H)** of CDCs, CFSPhs and CFs after conditioning with serum-free media for 7 days. **M)** EV yield (particle number per T75 culture flask) of CFSPhs approached CDC-EV yield. **N)** Exemplary size distribution of particles in a CFSPh-EV preparation. **O)** MiR-146a expression in CDC-/CFSPh-/CF-EVs. Data are represented as mean +/- SEM, * $p < 0.05$, ** $p < 0.01$, *** $p < 0.001$. Parts of the figure were created with Biorender.com.

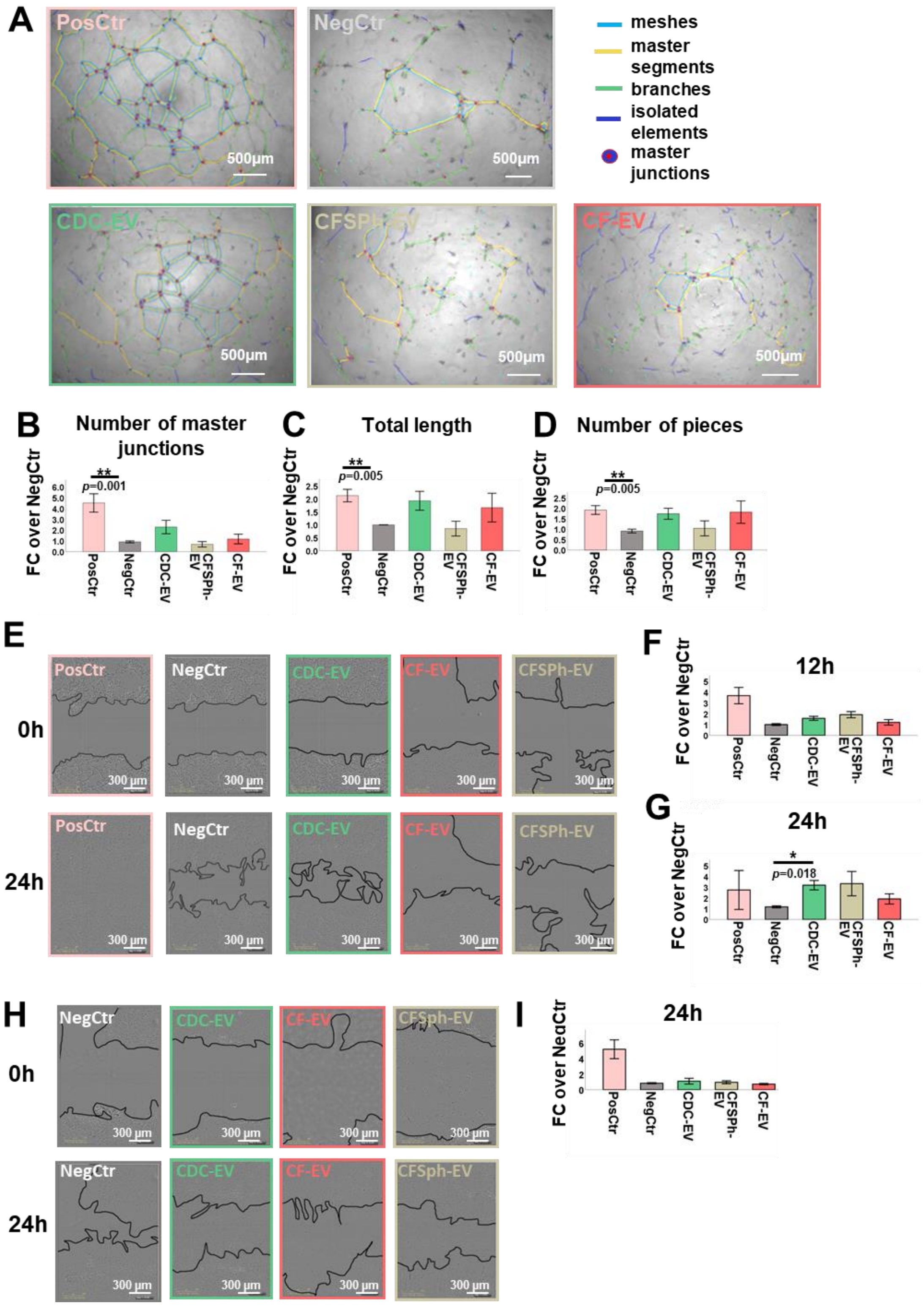

Supplemental Figure S14: Sphere formation as a prerequisite for regenerative characteristics - characterization of neonatal CFSPhs compared to CDCs and CFs of the same age. All samples included were derived from neonatal patients (age ≤ 21 days).

A-D) Tube formation assay with humane ECs on matrigel (matrigel assay). **A)** Exemplary photographs of positive control (PosCtr, EC medium with supplements), negative control (NegCtr, serum-free medium), serum-free medium supplemented with CDC-, CFSPh- and CF-EVs. Angiogenesis Analyzer (ImageJ) highlights structures such as master segments, branches, isolated elements and master junctions. "Total length" (the sum of length of segments, isolated elements and branches in the analyzed area), "Total master segments length" (the sum of the length of the detected master segments in the analyzed area) and the "number of pieces" (the sum of number of segments, isolated elements and branches detected in the analyzed area) were calculated and normalized to the negative control (FC over NegCtr). **B-D)** Quantitative analysis of selected parameters. K-M) Migration assay (Scratch assay) with human ECs. **E)** Exemplary pictures of ECs incubated with EC medium with supplements (PosCtr), serum-free medium (NegCtr), CDC-, CFSPh- and CF-EVs preparations in serum-free medium after the scratch (0h) and after 24h. **F-G)** Comparison of the difference of cell-free area between time point 0h and 12h **(F)** or 24h **(G)**, normalized to the negative control (FC over NegCtr).

H-I) Migration assay (scratch assay) with human CFs. **H)** Exemplary pictures of human CFs incubated with MEF medium (PosCtr), serum-free medium (NegCtr), CDC-, CFSPh- and CF-EVs in serum-free media directly after the scratch (0h) and after 24h. **I)** Comparison of the difference of cell-free area between the time point 0h and 24h normalized to the negative control (FC over NegCtr).

Data are represented as mean +/- SE, * $p < 0.05$, ** $p < 0.01$, *** $p < 0.001$. Parts of the figure were created with Biorender.com.

2. Supplemental Video Legends

Supplemental Videos V1-6: Scratch assays with human ECs. Picture series of EC scratch assays captured by the IncuCyte ZOOM® 96-Well Migration Assay System (Essen Biosciences, Hertfordshire, UK) in one hour intervals for exemplary wells of PosCtr, NegCtr and infant/adult CDC-/CFSPh-/CF-EV preparations.

V1: EC Scratch PosCtr
V2: EC Scratch NegCtr
V3: EC Scratch Infant CDC
V4: EC Scratch Infant CF
V5: EC Scratch Adult CDC
V6: EC Scratch Adult CF

Supplemental Video V7: Video of beating NRCM before the apoptosis assay. NRCMs used for the apoptosis test were controlled for their quality by careful screening for beating areas two days after seeding. If the majority of cells in one well were beating, this well was used for the apoptosis test and treated with cobalt chloride. This exemplary video shows beating NRCMs just before cobalt chloride treatment. Video filmed by Nikon Eclipse Ts2 (Nikon, Minato, Japan) with a 4x objective (phase contrast).

V7: NRCMs beating before cobalt chloride treatment

Supplemental Videos V8-15: Scratch assay with human CFs. Photography series of CF-scratch assays captured by the IncuCyte ZOOM® 96-Well Migration Assay System (Essen Biosciences, Hertfordshire, UK) in one hour intervals for exemplary wells of PosCtr, NegCtr and infant/adult CDC-/CFSPh-/CF-EV preparations.

V8: CF Scratch PosCtr
V9: CF Scratch NegCtr
V10: CF Scratch Infant CDC
V11:CF Scratch Infant CF
V12: CF Scratch Adult CDC
V13: CF Scratch Adult CF
V14: EC Scratch Infant CFSPh
V15: CF Scratch Infant CFSPh

3. Supplemental Tables

Supplemental Table 1: Adult Patients

Nr.	Sex	Age (years)	Surgery	Diagnosis
**A1**	male	61	CABG + MVR	atherosclerotic heart disease
**A2**	male	58	CABG	atherosclerotic heart disease
**A3**	male	55	CABG	atherosclerotic heart disease
**A4**	male	58	CABG	atherosclerotic heart disease
**A5**	male	62	AVR + Aorta + MAZE	combined aortic vitium
**A6**	female	76	AVR	aortic stenosis
**A7**	male	68	CABG	atherosclerotic heart disease
**A8**	female	59	AVR + MVR + MAZE	combined aortic vitium
**A9**	male	72	AVR + MAZE	aortic stenosis
**A10**	female	73	AVR	aortic stenosis
**A11**	male	67	CABG	atherosclerotic heart disease
**A12**	male	66	CABG	atherosclerotic heart disease
**A13**	male	63	CABG	atherosclerotic heart disease
**A14**	male	56	CABG	atherosclerotic heart disease
**A15**	male	70	CABG + Aorta	atherosclerotic heart disease
**A16**	female	64	CABG	atherosclerotic heart disease
**A17**	male	66	CABG	atherosclerotic heart disease
**A18**	male	54	MVR+TVR+ MAZE	mitral valve insufficiency
**A19**	male	67	CABG	atherosclerotic heart disease
**A20**	male	76	CABG	atherosclerotic heart disease
**A21**	male	61	CABG	atherosclerotic heart disease
**A22**	female	75	MVR + TVR + Tumor	biatrial tumor and mitral valve insufficiency
**A23**	female	72	AVR	aortic stenosis
**A24**	female	41	AVR	aortic stenosis
**A25**	male	58	AVR	aortic stenosis

Abbreviations: CABG: coronary artery bypass surgery, MVR: mitral valve replacement, AVR: aortic valve replacement, MAZE: MAZE procedure for atrial fibrillation, TVR: tricuspid valve replacement.

Supplemental Table 2: Pediatric patients

ID	Sex	Age (years)	Age (months)	Age (days)	STS code	Main diagnosis
**1**	male			21	69	HLHS
**2**	male			9	69	HLHS
**3**	female			7	96	hypoplastic aortic arch
**4**	male			6	85	TGA, VSD
**5**	male			7	90	DORV, TGA-type

**6**	male			10	69	HLHS
**7**	male			11	69	HLHS
**8**	male			6	69	HLHS
**9**	male			6	83	TGA, IVS
**10**	female			10	69	HLHS
**11**	female			10	69	HLHS
**12**	male			8	69	HLHS
**13**	male			9	69	HLHS
**14**	female			13	69	HLHS
**15**	male			19	69	HLHS
**16**	female			9	69	HLHS
**17**	male			12	2	ASD, secudum
**18**	female			12	115	Atrial isomerism right
**19**	female			6	69	HLHS
**20**	male			19	69	HLHS
**21**	female			6	69	HLHS
**22**	male			17	83	TGA, IVS
**23**	male			14	69	HLHS
**24**	male			5	69	HLHS
**25**	male			10	69	HLHS
**26**	male			12	69	HLHS
**27**	male			7	69	HLHS
**28**	female			7	69	HLHS
**29**	male			6	2	ASD, secundum
**30**	male			8	69	HLHS
**31**	female			11	69	HLHS
**32**	female			7	69	HLHS
**33**	female		1		121	Hypoplastic LV
**34**	male		3		69	HLHS
**35**	male		22		69	HLHS
**36**	female	1			69	HLHS
**37**	male	1			10	AVC (AVSD), complete CAVSD
**38**	male	2			77	Single ventricle, tricuspid atresia
**39**	male	2			69	HLHS
**40**	male	2			69	HLHS
**41**	female	2			69	HLHS
**42**	female	3			99	Patent ductus arteriosus
**43**	male	3			69	HLHS
**44**	female	3			78	Single ventricle, unbalanced AVC
**45**	male	3			77	Single ventricle, tricuspid atresia
**46**	female	3			10	AVC (AVSD), complete CAVSD

**47**	female	4			16	Truncus arteriosus
**48**	male	4			74	Single ventricle, DILV
**49**	female	6			69	HLHS

Abbreviations: STS code: diagnostic code of the Society of Thoracic Surgeons. HLHS: hypoplastic left heart syndrome, TGA: transposition of the great arteries, VSD: ventricular septal defect, DORV: double-outlet left ventricle, IVS: intact ventricular septum, ASD secundum: atrial septal defect type 2, LV: left ventricle, AVC: atrioventricular canal, (C) AVSD: (complete) atrioventricular spetal defect, DILV: double-inlet left ventricle.

Supplemental Table 3: Significant differences in marker expression (qRT-PCR, Figure 1 and Suppl. Figure 1)

Marker (alphabetical)	Cell type 1	Cell type 2	Significance test	p-value
ALDH1A2	AFs	CFs	Dunnett-T3	0.001
ALDH1A2	CDCs	CFs	Dunnett-T3	0.000
ALDH1A2	CDCs	SMCs	Dunnett-T3	0.001
ALDH1A2	CDCs	DIFF D8	Dunnett-T3	0.006
ALDH1A2	CFs	SMCs	Dunnett-T3	0.000
ALDH1A2	CFs	ECs	Dunnett-T3	0.000
ALDH1A2	CFs	DIFF D6	Dunnett-T3	0.001
ALDH1A2	CFs	DIFF D8	Dunnett-T3	0.000
CDH5	AFs	CDCs	Dunnett-T3	0.049
CDH5	CDCs	CFs	Dunnett-T3	0.024
CDH5	CDCs	SMCs	Dunnett-T3	0.016
CDH5	CDCs	DIFF D6	Dunnett-T3	0.019
CDH5	CDCs	DIFF D8	Dunnett-T3	0.016
DDR2	AFs	ECs	Dunnett-T3	0.043
DDR2	AFs	DIFF D8	Dunnett-T3	0.040
DDR2	CDCs	DIFF D6	Dunnett-T3	0.001
DDR2	CDCs	DIFF D8	Dunnett-T3	0.001
DDR2	CFs	DIFF D6	Dunnett-T3	0.002
DDR2	CFs	DIFF D8	Dunnett-T3	0.001
DDR2	SMCs	DIFF D6	Dunnett-T3	0.030
DDR2	SMCs	DIFF D8	Dunnett-T3	0.016
DDR2	ECs	AFs	Dunnett-T3	0.043
ENG (CD105)	AFs	DIFF D6	Dunnett-T3	0.016
ENG (CD105)	AFs	DIFF D8	Dunnett-T3	0.016
ENG (CD105)	CDCs	DIFF D6	Dunnett-T3	0.000
ENG (CD105)	CDCs	DIFF D8	Dunnett-T3	0.000
ENG (CD105)	CFs	DIFF D6	Dunnett-T3	0.000
ENG (CD105)	CFs	DIFF D8	Dunnett-T3	0.000
GATA4	AFs	CDCs	Dunnett-T3	0.000
GATA4	AFs	DIFF D6	Dunnett-T3	0.001
GATA4	CDCs	SMCs	Dunnett-T3	0.000
GATA4	CDCs	ECs	Dunnett-T3	0.000

Marker (alphabetical)	Cell type 1	Cell type 2	Significance test	p-value
GATA4	CDCs	DIFF D6	Dunnett-T3	0.002
GATA4	CFs	DIFF D6	Dunnett-T3	0.002
GATA4	SMCs	DIFF D6	Dunnett-T3	0.001
GATA4	ECs	DIFF D6	Dunnett-T3	0.001
NKX2-5	CDCs	DIFF D6	Dunnett-T3	0.019
NKX2-5	CFs	DIFF D6	Dunnett-T3	0.019
NKX2-5	SMCs	DIFF D6	Dunnett-T3	0.019
NKX2-5	ECs	DIFF D6	Dunnett-T3	0.019
PDGFRA	CDCs	DIFF D6	Dunnett-T3	0.011
PDGFRA	CFs	DIFF D6	Dunnett-T3	0.005
PDGFRA	ECs	DIFF D6	Dunnett-T3	0.002
PDGFRA	DIFF D6	DIFF D8	Dunnett-T3	0.000
PDGFRA	DIFF D8	DIFF D6	Dunnett-T3	0.000
PDGFRB	CDCs	DIFF D6	Dunnett-T3	0.037
PECAM1 (CD31)	AFs	ECs	Dunnett-T3	0.006
PECAM1 (CD31)	CDCs	ECs	Dunnett-T3	0.008
PECAM1 (CD31)	CDCs	DIFF D6	Dunnett-T3	0.030
PECAM1 (CD31)	CDCs	DIFF D8	Dunnett-T3	0.020
PECAM1 (CD31)	CFs	ECs	Dunnett-T3	0.008
PECAM1 (CD31)	SMCs	ECs	Dunnett-T3	0.008
PECAM1 (CD31)	ECs	DIFF D6	Dunnett-T3	0.008
PECAM1 (CD31)	ECs	DIFF D8	Dunnett-T3	0.008
TAGLN	CFs	DIFF D6	Dunnett-T3	0.004
TAGLN	CFs	DIFF D8	Dunnett-T3	0.002
TAGLN	SMCs	DIFF D6	Dunnett-T3	0.000
TAGLN	SMCs	DIFF D8	Dunnett-T3	0.000
TBX5	AFs	CDCs	Dunnett-T3	0.049
TBX5	AFs	DIFF D6	Dunnett-T3	0.006
TBX5	AFs	DIFF D8	Dunnett-T3	0.004
TBX5	CDCs	ECs	Dunnett-T3	0.000
TBX5	CDCs	DIFF D6	Dunnett-T3	0.014
TBX5	CDCs	DIFF D8	Dunnett-T3	0.006
TBX5	CFs	DIFF D8	Dunnett-T3	0.028
TBX5	SMCs	DIFF D6	Dunnett-T3	0.014
TBX5	SMCs	DIFF D8	Dunnett-T3	0.006
TBX5	ECs	DIFF D6	Dunnett-T3	0.005
TBX5	ECs	DIFF D8	Dunnett-T3	0.004
THY1 (CD90)	CDCs	DIFF D6	Dunnett-T3	0.000
THY1 (CD90)	CDCs	CFs	Games-Howell	0.037
THY1 (CD90)	CFs	ECs	Dunnett-T3	0.002
THY1 (CD90)	CFs	DIFF D6	Dunnett-T3	0.000
THY1 (CD90)	CFs	DIFF D8	Dunnett-T3	0.037

Marker (alphabetical)	Cell type 1	Cell type 2	Significance test	p-value
THY1 (CD90)	SMCs	ECs	Dunnett-T3	0.023
THY1 (CD90)	SMCs	DIFF D6	Dunnett-T3	0.000
THY1 (CD90)	ECs	CFs	Dunnett-T3	0.002
THY1 (CD90)	ECs	SMCs	Dunnett-T3	0.023

Abbreviations: AFs: adipose tissue-derived fibroblasts, CDCs: cardiosphere-derived cells, CFs: cardiac fibroblasts, SMCs: smooth muscle cells, ECs: endothelial cells, DIFF D6: human induced pluripotent cells on day 6 of cardiac differentiation protocol, DIFF D8: human induced pluripotent cells on day 8 of cardiac differentiation protocol.

Supplemental Table 4: Significant differences in marker expression (flow cytometry, Figure 1 and Suppl. Figure S1)

Marker	Cell type 1	Cell type 2	Significance test	*p*-value
CD31	ECs	CDCs	Dunnett-T3	0.030
CD31	ECs	AFs	Dunnett-T3	0.030
CD31	ECs	CFs	Dunnett-T3	0.015
CD31	ECs	SMCs	Dunnett-T3	0.029
CD90	ECs	AFs	Dunnett-T3	0.004
CD90	ECs	CFs	Dunnett-T3	0.002
CD90	ECs	SMCs	Dunnett-T3	0.035

Abbreviations: AFs: adipose tissue-derived fibroblasts, CDCs: cardiosphere-derived cells, CFs: cardiac fibroblasts, SMCs: smooth muscle cells, ECs: endothelial cells.

Supplemental Table 5: Cell samples for sc-RNAseq

Sample No.	Cell type	Age group	Passage	sex	Patients' age	surgery	diagnosis	ID
1	ECs	Adult	P1	male	63 years	CABG	atherosclerotic heart disease	A13
2	CFs	Adult	P2	male	63 years	CABG	atherosclerotic heart disease	A13
3	SMCs	Adult	P2	male	66 years	CABG	atherosclerotic heart disease	A12
4	CDCs	Adult	P0	male	61 years	CABG	atherosclerotic heart disease	A21
5	CDCs	Infant	P0	female	7 days	Norwood	HLHS	32

Abbreviations: sc-RNAseq: single-cell RNA sequencing, ECs: endothelial cells, CFs: cardiac fibroblasts, SMCs: smooth muscle cells, CDCs: cardiosphere-derived cells, P0/2: cell culture passage 0/2, CABG: coronary artery bypass graft, Norwood: Norwood I procedure for single-ventricle patients.

Supplemental Table 6: Filtering parameters in Seurat

Sample No. / Name	Genes/cell	UMI count/cell	% MT Genes	Cells before filtering	Cells after filtering
1/ ECs	> 200	< 55000	< 20	672	668
2/ CFs	No filtering	No filtering	No filtering	457	457
3/ SMCs	> 200	No filtering	< 20	585	581
4/ CDCs adult	> 200	< 150000	< 20	1196	1109
5/ CDCs infant	> 200	< 120000	< 20	1060	960

Abbreviations: ECs: endothelial cells, CFs: cardiac fibroblasts, SMCs: smooth muscle cells, CDCs: cardiosphere-derived cells, Genes/cell: number of genes detected per cell; UMI count/cell: number of unique molecular identifiers (UMIs) per cell; % MT Genes: percentage of mitochondrial genes per cell

Supplemental Table 7: Quality control parameters after filtering

Sample No. / Name	Genes/cell (median)	UMI count/cell (median)	Reads per cell (mean)	% MT Genes (median)
1/ECs	2178.5	7762.5	58,268	3.0
2/CFs	3043	14373	122,176	1.3
3/SMCs	3924	21437	117,533	2.5
4/CDCs (adult)	5803	36171	99,694	6.0
5/CDCs (infant)	5549	32482	100,206	6.9

Abbreviations: ECs: endothelial cells, CFs: cardiac fibroblasts, SMCs: smooth muscle cells, CDCs: cardiosphere-derived cells, Genes/cell: number of genes detected per cell; UMI count/cell: number of unique molecular identifiers (UMIs) per cell; % MT Genes: percentage of mitochondrial genes per cell.

Supplemental Table 8: Enriched GO terms in Clusters 0 – 10 from Fig 2C ordered in topics

	Cell type and cluster	SMC-0	SMC-5	EC-1	EC-6	EC-10	CF-2	CF-9	CDC-3	CDC-4	CDC-5	CDC-7	CDC-8
	amount of cells of each cell type belonging to respective cluster	90%	6%	61%	28%	8%	87%	13%	26%	24%	20%	15%	14%
Angiogenesis	GO_CIRCULATORY_SYSTEM_DEVELOPMENT	17.56%		19.78%			12.89%						
	GO_TUBE_MORPHOGENESIS	13.03%		18.16%									
	GO_TUBE_DEVELOPMENT	14.45%		19.24%									
	GO_BLOOD_VESSEL_MORPHOGENESIS	11.90%		16.53%									
	GO_CARDIOVASCULAR_SYSTEM_DEVELOPMENT	13.88%		17.89%									
Extracellular matrix/ structure organization	GO_EXTRACELLULAR_MATRIX_STRUCTURAL_CONSTITUENT	5.67%					7.40%	4.32%					
	GO_COLLAGEN_CONTAINING_EXTRACELLULAR_MATRIX	9.92%					13.60%	7.05%					
	GO_EXTRACELLULAR_MATRIX	10.76%					13.60%	7.27%					
	GO_EXTRACELLULAR_STRUCTURE_ORGANIZATION	9.07%					9.79%						
Response to stress	GO_REGULATION_OF_RESPONSE_TO_STRESS								18.95%	15.55%		17.13%	
	GO_REGULATION_OF_CELLULAR_RESPONSE_TO_STRESS												9.51%
Immune system process	GO_POSITIVE_REGULATION_OF_IMMUNE_SYSTEM_PROCESS								14.80%				
	GO_REGULATION_OF_IMMUNE_SYSTEM_PROCESS								18.95%				
	GO_CELL_ACTIVATION_INVOLVED_IN_IMMUNE_RESPONSE		12.92%						13.36%		12.92%		
	GO_LEUKOCYTE_MEDIATED_IMMUNITY		14.21%						14.26%		14.21%		
	GO_MYELOID_LEUKOCYTE_MEDIATED_IMMUNITY		12.40%						12.27%		12.40%	10.40%	
	GO_IMMUNE_EFFECTOR_PROCESS		17.05%						17.69%		17.05%	14.53%	
	GO_MYELOID_LEUKOCYTE_ACTIVATION		12.92%						13.90%		12.92%	10.70%	
	GO_ANTIGEN_PROCESSING_AND_PRESENTATION_OF_EXOGENOUS_PEPTIDE_ANTIGEN_VIA_MHC_CLASS_I		5.94%								5.94%	4.74%	
	GO_ANTIGEN_PROCESSING_AND_PRESENTATION_OF_PEPTIDE_ANTIGEN_VIA_MHC_CLASS_I		6.20%								6.20%		
	GO_ANTIGEN_PROCESSING_AND_PRESENTATION_OF_PEPTIDE_ANTIGEN		7.75%								7.75%		
Response to cytokine	GO_RESPONSE_TO_CYTOKINE	13.31%	16.80%						20.58%	13.91%	16.80%	17.58%	12.55%
	GO_CYTOKINE_MEDIATED_SIGNALING_PATHWAY		13.70%						14.98%		13.70%	12.08%	
	GO_RESPONSE_TO_GROWTH_FACTOR	12.75%											
Secretion	GO_SECRETORY_VESICLE		17.83%						15.70%		17.83%	12.84%	
	GO_SECRETORY_GRANULE		15.50%						15.16%		15.50%	12.08%	
	GO_SECRETION		19.90%						22.38%		19.90%		
Negative regulation of cell cycle	GO_NEGATIVE_REGULATION_OF_CELL_CYCLE_PROCESS									7.89%			
	GO_NEGATIVE_REGULATION_OF_CELL_CYCLE_PHASE_TRANSITION									6.79%			
	GO_NEGATIVE_REGULATION_OF_MITOTIC_CELL_CYCLE									7.45%			
	GO_NEGATIVE_REGULATION_OF_CELL_CYCLE									10.62%			
Cell Cycle	GO_REGULATION_OF_CELL_CYCLE_PHASE_TRANSITION									9.42%			
	GO_REGULATION_OF_CELL_CYCLE_G2_M_PHASE_TRANSITION									6.57%			
	GO_CELL_CYCLE_G2_M_PHASE_TRANSITION									7.56%		7.19%	
	GO_REGULATION_OF_MITOTIC_CELL_CYCLE							8.86%		12.38%		11.01%	
	GO_REGULATION_OF_CELL_CYCLE_PROCESS							8.64%		13.80%		11.47%	
	GO_MITOTIC_CELL_CYCLE				14.35%			14.32%		27.27%		15.60%	
	GO_CELL_CYCLE				20.68%			21.82%		27.27%		21.71%	18.25%
	GO_CELL_CYCLE_PHASE_TRANSITION				8.65%			9.09%		12.92%		10.70%	
	GO_REGULATION_OF_CELL_CYCLE				13.08%			15.00%		17.74%		15.44%	
	GO_CELL_CYCLE_PROCESS				17.30%	12.61%		17.05%		22.45%		16.97%	
	GO_CELL_DIVISION				10.76%	9.13%		10.45%		12.49%			
	GO_MITOTIC_NUCLEAR_DIVISION					6.09%		5.68%		7.34%			
	GO_REGULATION_OF_CELL_POPULATION_PROLIFERATION	17.28%											
Mitochondrion/ Cellular respiration	GO_MITOCHONDRIAL_TRANSPORT							5.00%					
	GO_MITOCHONDRIAL_MEMBRANE_PART		10.08%				6.21%				10.08%		
	GO_MITOCHONDRIAL_ENVELOPE		18.86%		9.49%	10.87%		10.68%			18.86%		9.89%
	GO_MITOCHONDRION		26.10%		14.98%	14.35%		16.59%			26.10%		17.49%
	GO_MITOCHONDRION_ORGANIZATION		14.21%		7.81%	8.26%		7.73%			14.21%		
	GO_MITOCHONDRIAL_PART		21.45%			11.74%		13.41%			21.45%		12.17%
	GO_MITOCHONDRIAL_PROTEIN_COMPLEX		10.59%			6.96%		5.23%			10.59%		
	GO_INNER_MITOCHONDRIAL_MEMBRANE_PROTEIN_COMPLEX		9.56%								9.56%		
	GO_ATP_SYNTHESIS_COUPLED_ELECTRON_TRANSPORT		8.79%								8.79%		
	GO_CELLULAR_RESPIRATION		9.56%								9.56%		
	GO_OXIDATION_REDUCTION_PROCESS		20.16%								20.16%		
	GO_ELECTRON_TRANSPORT_CHAIN		11.63%								11.63%		
	GO_ENERGY_DERIVATION_BY_OXIDATION_OF_ORGANIC_COMPOUNDS		10.59%								10.59%		
	GO_OXIDOREDUCTASE_COMPLEX		6.46%								6.46%		
	GO_OXIDOREDUCTASE_ACTIVITY		19.12%								19.12%		
	GO_ELECTRON_TRANSFER_ACTIVITY		7.24%								7.24%		
	GO_RESPIRATORY_CHAIN_COMPLEX		7.75%								7.75%		
	GO_RESPIRATORY_ELECTRON_TRANSPORT_CHAIN		9.04%								9.04%		
	GO_RESPIRASOME		8.53%								8.53%		
Cell motility	GO_LOCOMOTION	19.83%		22.76%					20.76%				
	GO_CELL_MOTILITY	18.13%							19.13%				
Cell activation	GO_CELL_ACTIVATION		17.57%						20.04%		17.57%		
Adhesion	GO_CELL_JUNCTION			25.47%		12.17%	14.08%		16.25%				
	GO_BIOLOGICAL_ADHESION			20.87%			15.27%		16.61%				
	GO_CELL_ADHESION_MOLECULE_BINDING								11.37%	9.31%		10.86%	12.55%
	GO_CADHERIN_BINDING									7.89%		9.17%	11.03%
Development/ Differentiation	GO_ANATOMICAL_STRUCTURE_FORMATION_INVOLVED_IN_MORPHOGENESIS	14.45%		20.05%									
	GO_SKELETAL_SYSTEM_DEVELOPMENT	9.63%											
	GO_ANIMAL_ORGAN_MORPHOGENESIS	13.31%											
	GO_NEGATIVE_REGULATION_OF_MULTICELLULAR_ORGANISMAL_PROCESS	15.01%											
	GO_POSITIVE_REGULATION_OF_MULTICELLULAR_ORGANISMAL_PROCESS	16.71%							18.41%				
	GO_POSITIVE_REGULATION_OF_DEVELOPMENTAL_PROCESS	14.73%							15.70%				
	GO_REGULATION_OF_CELL_DIFFERENTIATION	17.85%							19.31%				
	GO_REGULATION_OF_ANATOMICAL_STRUCTURE_MORPHOGENESIS								17.87%				
Catabolic process	GO_REGULATION_OF_CELLULAR_CATABOLIC_PROCESS											12.23%	
	GO_ENDOPEPTIDASE_COMPLEX											4.89%	
	GO_REGULATION_OF_MRNA_CATABOLIC_PROCESS											7.49%	
	GO_REGULATION_OF_CATABOLIC_PROCESS											13.30%	
Actin cytoskeleton	GO_ACTIN_CYTOSKELETON												7.98%
	GO_ACTIN_FILAMENT_BASED_PROCESS												10.27%

Colored GO_Term in column 2 indicates that this term is specifc for the respective cell type; Color code: red: CF; purple: ECs, blue: SMCs, green: CDCs; Percentages indicate the number of uDEGs of a cluster in overlap with the respective gene set (mentioned in column 2).

Supplemental Table 9: Right atrial tissue biopsies for single nuclei and single cell RNA sequencing

Sample ID	Cardiac tissue	SN or SC	Age group	sex	Patients' age	surgery	diagnosis	note	ID
RA-1	Right atrial appendage	SN	Adult	female	41 years	AVR	aortic stenosis	-	A24
RA-2	Right atrial appendage	SN	Adult	male	58 years	AVR	aortic stenosis	-	A25
RA-3	Right atrial appendage	SC	Adult	female	75 years	MVR, TVR, tumor resection,	biatrial tumor and mitral valve insufficiency	Tissue was not taken from tumor associated region	A22
RA-4	Right atrial appendage	SC	Adult	female	72 years	AVR	aortic stenosis	-	A23

Abbreviations: RA: right atrium, SN: single nuclei RNA sequencing, SC: single cell RNA sequencing, AVR: aortic valve replacement, MVR: mitral valve replacement, TVR: tricuspid valve replacement.

Supplemental Table 10: Top 25 specific Genes of Cluster 0, 7 and 12 in Suppl Fig S7D

Cluster 0	Cluster 7	Cluster 12
KAZN	KAZN	ENO1
PLA2G5	UAP1	SRM
NEGR1	RANBP2	S100A16
COL4A4	PGAP1	PSMD2
MAPK10	ATP1B3	UCHL1
UNC5C	PRMT9	CXCL1
LAMA2	FAT1	HMGA1
EBF2	ELL2	TFPI2
ZFPM2	DPYSL3	GNG11
FREM1	TBX18	SERPINE1
COL15A1	AKAP12	TPM2
ADAMTSL2	TBX20	PPP1R14B
CELF2	ESYT2	GAPDH
ARHGAP21	FGFR1	C12orf75
BICC1	NR4A3	MRPL52
MIR100HG	ABL1	ANXA2
PCDH9	NR4A1	MT2A
GPC6	MEG3	NQO1
FGF14	CRISPLD2	NME1
RORA	USP36	NME2
ADAMTSL3	WDR45B	AP2S1

ABCA9	RNF152	LGALS1
ABCA6	AXL	CXCL6
ABCA10	ZNF331	PHLDA2
FTX	TSHZ2	MMP1

Coloured genes were used for gene expression UMAP plots in Fig 3E

Suppl. Table 11: Significant differences between age groups and cell types (Fig 4A-C)

Marker	Group	Subgroup 1	Subgroup 2	Significance test	*p*-value
GATA4	Infant	CDCs	CFs	ANOVA	0.000
GATA4	CDCs	Infant	Adult	ANOVA	0.012
TBX5	Infant	CDCs	CFs	Welch test	0.000
TBX5	CDCs	Infant	Adult	ANOVA	0.001
NKX2-5	CDCs	Infant	Adult	Welch test	0.012
NKX2-5	CFs	Infant	Adult	Welch test	0.000
ALDH1A2	Infant	CDCs	CFs	Welch test	0.001
ALDH1A2	Adult	CDCs	CFs	Welch test	0.000
ALDH1A2	CDCs	Infant	Adult	Welch test	0.009
ALDH1A2	CFs	Infant	Adult	ANOVA	0.012
S100A4	Infant	CDCs	CFs	Welch test	0.032
S100A4	Adult	CDCs	CFs	Welch test	0.014
THY1 (*CD90*)	Infant	CDCs	CFs	Welch test	0.000
THY1 (*CD90*)	Adult	CDCs	CFs	Welch test	0.008
THY1 (*CD90*)	CDCs	Infant	Adult	Welch test	0.000
THY1 (*CD90*)	CFs	Infant	Adult	Welch test	0.000
CXCL6	Infant	CDCs	CFs	Welch test	0.017
CXCL6	Adult	CDCs	CFs	Welch test	0.015
miR-146a-5p	Infant	CDCs	CFs	Welch test	0.001

Abbreviations: CDCs: cardiosphere-derived cells, CFs: cardiac fibroblasts, Infant: pediatric patients group (see Suppl. Table 2), Adult: adult patients group (see Suppl. Table 1).

Supplemental Table 12: Enriched GO terms in clusters of Fig 4E ordered in topics

	Cluster	Cl0	Cl1	Cl2	Cl3	Cl4	Cl5
	**amount of infant CDCs belonging to respective cluster**	26.0%	56.0%	0.0%	16.0%	1.0%	0.5%
	**amount of adult CDCs belonging to respective cluster**	28.0%	0.0%	33.0%	21.0%	11.0%	7.0%
**Angiogenesis**	GO_TUBE_DEVELOPMENT		20.87%	17.49%			
	GO_CIRCULATORY_SYSTEM_DEVELOPMENT		25.24%	18.63%			
	GO_BLOOD_VESSEL_MORPHOGENESIS		17.96%				
	GO_TUBE_MORPHOGENESIS		19.90%				
**Extracellular Matrix/ Structure**	GO_EXTRACELLULAR_MATRIX_STRUCTURAL_CONSTITUENT		15.53%	9.13%			
	GO_EXTRACELLULAR_STRUCTURE_ORGANIZATION		23.30%	18.25%			
	GO_COLLAGEN_CONTAINING_EXTRACELLULAR_MATRIX		22.82%	17.87%			
	GO_EXTRACELLULAR_MATRIX		23.79%	19.39%			
	GO_EXTRACELLULAR_MATRIX_COMPONENT		5.34%				
	GO_EXTRACELLULAR_MATRIX_STRUCTURAL_CONSTITUENT_CONFERRING_TENSILE_STRENGTH		5.83%				
	GO_COLLAGEN_FIBRIL_ORGANIZATION		6.31%				
	GO_COLLAGEN_TRIMER		7.28%				
**Immune system process**	GO_MYELOID_LEUKOCYTE_MEDIATED_IMMUNITY			14.83%			
	GO_MYELOID_LEUKOCYTE_ACTIVATION			17.87%			
	GO_LEUKOCYTE_MEDIATED_IMMUNITY			17.49%			
	GO_IMMUNE_EFFECTOR_PROCESS			20.15%			
	GO_CELL_ACTIVATION_INVOLVED_IN_IMMUNE_RESPONSE			15.21%			
**Response to cytokine**	GO_RESPONSE_TO_CYTOKINE			20.53%			12.07%
**Secretion**	GO_SECRETION			25.48%			
	GO_SECRETORY_VESICLE			19.01%			12.07%
	GO_SECRETORY_GRANULE			18.63%			11.21%
	GO_REGULATION_OF_VESICLE_MEDIATED_TRANSPORT						8.62%
	GO_VESICLE_ORGANIZATION						6.03%
	GO_VESICLE_MEMBRANE						10.34%
**Negative Regulation of Cell Cycle**	GO_NEGATIVE_REGULATION_OF_METAPHASE_ANAPHASE_TRANSITION_OF_CELL_ CYCLE				5.76%		
	GO_NEGATIVE_REGULATION_OF_NUCLEAR_DIVISION				6.10%		
	GO_NEGATIVE_REGULATION_OF_CHROMOSOME_SEGREGATION				6.10%		
	GO_NEGATIVE_REGULATION_OF_CELL_CYCLE				14.24%		
**Cell Cycle/Proliferation**	GO_REGULATION_OF_CELL_POPULATION_PROLIFERATION		19.90%	25.48%			
	GO_POSITIVE_REGULATION_OF_CELL_POPULATION_PROLIFERATION		14.56%				
	GO_CELL_CYCLE				41.36%		18.97%
	GO_CELL_DIVISION				25.42%		8.62%
	GO_REGULATION_OF_CELL_CYCLE				26.78%		12.93%
	GO_CELL_CYCLE_PROCESS				35.25%		
	GO_MITOTIC_CELL_CYCLE				32.54%		
	GO_REGULATION_OF_CELL_CYCLE_PROCESS				21.02%		
	GO_REGULATION_OF_MITOTIC_CELL_CYCLE				18.64%		
	GO_MITOTIC_NUCLEAR_DIVISION				18.64%		
	GO_CHROMOSOME_SEGREGATION				18.31%		
	GO_CELL_CYCLE_PHASE_TRANSITION				17.63%		
	GO_NUCLEAR_CHROMOSOME_SEGREGATION				16.27%		
	GO_SISTER_CHROMATID_SEGREGATION				15.59%		
	GO_MITOTIC_SISTER_CHROMATID_SEGREGATION				13.90%		
	GO_REGULATION_OF_NUCLEAR_DIVISION				12.54%		
	GO_REGULATION_OF_CELL_CYCLE_PHASE_TRANSITION				12.20%		
	GO_KINETOCHORE				12.20%		
	GO_MICROTUBULE_CYTOSKELETON_ORGANIZATION_INVOLVED_IN_MITOSIS				9.83%		
	GO_REGULATION_OF_CHROMOSOME_SEGREGATION				9.49%		
	GO_MITOTIC_SPINDLE_ORGANIZATION				8.81%		
	GO_SPINDLE_POLE				8.14%		
	GO_REGULATION_OF_SISTER_CHROMATID_SEGREGATION				8.14%		
	GO_CHROMOSOME_SEPARATION				7.80%		
	GO_REGULATION_OF_CHROMOSOME_SEPARATION				7.12%		
	GO_MITOTIC_SPINDLE				7.12%		
	GO_METAPHASE_ANAPHASE_TRANSITION_OF_CELL_CYCLE				6.78%		
**Cell motility**	GO_LOCOMOTION		26.21%	28.14%			
	GO_CELL_MOTILITY		25.24%	27.00%			
	GO_POSITIVE_REGULATION_OF_LOCOMOTION		12.14%				
**Cell Activation**	GO_CELL_ACTIVATION		17.48%	26.62%			
**Adhesion**	GO_BIOLOGICAL_ADHESION		21.36%	26.62%			
	GO_CELL_ADHESION_MOLECULE_BINDING		12.62%	12.55%			
	GO_ANCHORING_JUNCTION	11.39%	11.65%	13.69%		25.87%	
	GO_CELL_JUNCTION					26.57%	
**Morphogenesis/ Development**	GO_ANATOMICAL_STRUCTURE_FORMATION_INVOLVED_IN_MORPHOGENESIS		21.36%	19.77%			
	GO_REGULATION_OF_ANATOMICAL_STRUCTURE_MORPHOGENESIS		16.50%	17.87%			
	GO_POSITIVE_REGULATION_OF_DEVELOPMENTAL_PROCESS			20.53%			
	GO_SKELETAL_SYSTEM_DEVELOPMENT		13.11%				
	GO_ANIMAL_ORGAN_MORPHOGENESIS		16.50%				
	GO_HEART_DEVELOPMENT		11.65%				
	GO_CARDIOVASCULAR_SYSTEM_DEVELOPMENT		20.87%				
**Mitochondiron/ Cellular Respiration**	GO_MITOCHONDRION	18.99%					14.66%
	GO_OXIDATION_REDUCTION_PROCESS						10.34%
	GO_MITOCHONDRIAL_PART						13.79%
	GO_MITOCHONDRIAL_MATRIX						7.76%
	GO_ENERGY_DERIVATION_BY_OXIDATION_OF_ORGANIC_COMPOUNDS						6.03%
	GO_CELLULAR_RESPIRATION						5.17%
**Catabolic Process**	GO_REGULATION_OF_CATABOLIC_PROCESS						10.34%
	GO_MACROMOLECULE_CATABOLIC_PROCESS	22.78%				38.46%	
	GO_CELLULAR_MACROMOLECULE_CATABOLIC_PROCESS	21.52%				37.76%	
	GO_ORGANIC_CYCLIC_COMPOUND_CATABOLIC_PROCESS	21.52%				32.87%	
	GO_RNA_CATABOLIC_PROCESS	18.35%				32.87%	
	GO_NUCLEAR_TRANSCRIBED_MRNA_CATABOLIC_PROCESS	15.82%				31.47%	

Colored GO_Term in column 2 indicates that this term is specifc for the respective cluster; Color code: brown: Cluster 0; light green: Cluster 1; dark green: Cluster 2, red: Cluster 3, yellow, Cluster 4, light blue: Cluster 5; Percentages indicate the number of uDEGs of a cluster in overlap with the respective gene set (mentioned in column 2).

Supplemental Table 13: Enriched GO terms in Clusters 1 and Cluster 2 from Fig 4E ordered in topics

	Cell type and cluster	CDC_infant _Cl1	CDC_adult _Cl2
	**amount of cells of CDCs belonging to respective cluster**	**56.00%**	**33.00%**
**Angiogenesis**	GO_TUBE_DEVELOPMENT	**20.87%**	**17.49%**
	GO_CIRCULATORY_SYSTEM_DEVELOPMENT	**25.24%**	**18.63%**
	GO_BLOOD_VESSEL_MORPHOGENESIS	**17.96%**	
	GO_TUBE_MORPHOGENESIS	**19.90%**	
**Extracellular Matrix/ Structure**	GO_EXTRACELLULAR_MATRIX_STRUCTURAL_CONSTITUENT	**15.53%**	**9.13%**
	GO_EXTRACELLULAR_STRUCTURE_ORGANIZATION	**23.30%**	**18.25%**
	GO_COLLAGEN_CONTAINING_EXTRACELLULAR_MATRIX	**22.82%**	**17.87%**
	GO_EXTRACELLULAR_MATRIX	**23.79%**	**19.39%**
	GO_EXTRACELLULAR_MATRIX_COMPONENT	**5.34%**	
	GO_EXTRACELLULAR_MATRIX_STRUCTURAL_CONSTITUENT_CONFERRING_TENSILE _STRENGTH	**5.83%**	
	GO_COLLAGEN_FIBRIL_ORGANIZATION	**6.31%**	
	GO_COLLAGEN_TRIMER	**7.28%**	
**Immune system process**	GO_MYELOID_LEUKOCYTE_MEDIATED_IMMUNITY		**14.83%**
	GO_MYELOID_LEUKOCYTE_ACTIVATION		**17.87%**
	GO_LEUKOCYTE_MEDIATED_IMMUNITY		**17.49%**
	GO_IMMUNE_EFFECTOR_PROCESS		**20.15%**
	GO_CELL_ACTIVATION_INVOLVED_IN_IMMUNE_RESPONSE		**15.21%**
**Response to cytokine**	GO_RESPONSE_TO_CYTOKINE		**20.53%**
**Secretion**	GO_SECRETION		**25.48%**
	GO_SECRETORY_VESICLE		**19.01%**
	GO_SECRETORY_GRANULE		**18.63%**
**Cell Cycle/Proliferation**	GO_REGULATION_OF_CELL_POPULATION_PROLIFERATION	**19.90%**	**25.48%**
	GO_POSITIVE_REGULATION_OF_CELL_POPULATION_PROLIFERATION	**14.56%**	
**Cell motility**	GO_LOCOMOTION	**26.21%**	**28.14%**
	GO_CELL_MOTILITY	**25.24%**	**27.00%**
	GO_POSITIVE_REGULATION_OF_LOCOMOTION	**12.14%**	
**Cell Activation**	GO_CELL_ACTIVATION	**17.48%**	**26.62%**
**Adhesion**	GO_BIOLOGICAL_ADHESION	**21.36%**	**26.62%**
	GO_CELL_ADHESION_MOLECULE_BINDING	**12.62%**	**12.55%**
	GO_ANCHORING_JUNCTION	**11.65%**	**13.69%**
**Morphogenesis/Development**	GO_ANATOMICAL_STRUCTURE_FORMATION_INVOLVED_IN_MORPHOGENESIS	**21.36%**	**19.77%**
	GO_REGULATION_OF_ANATOMICAL_STRUCTURE_MORPHOGENESIS	**16.50%**	**17.87%**
	GO_POSITIVE_REGULATION_OF_DEVELOPMENTAL_PROCESS		**20.53%**
	GO_SKELETAL_SYSTEM_DEVELOPMENT	**13.11%**	
	GO_ANIMAL_ORGAN_MORPHOGENESIS	**16.50%**	
	GO_HEART_DEVELOPMENT	**11.65%**	
	GO_CARDIOVASCULAR_SYSTEM_DEVELOPMENT	**20.87%**	

Colored GO_Term in column 2 indicates that this term is specifc for the respective cluster; Color code: light green: CDC_infant (Cluster 1); dark green: CDC_adult (Cluster 2); Percentages indicate the number of uDEGs of a cluster in overlap with the respective gene set (mentioned in column 2).

Supplemental Table 14: qRT-PCR primer sets (mRNA)

Official gene symbol	official name	Gene bank identification (ID)	5′→ 3′ (forward)	5′→ 3′ (reverse)
GATA4	*GATA binding protein 4*	NM_001308093, NM_001374274.1, NM_001374273.1, NM_002052.5, NM_001308094.2	GGA AGC CCA AGA ACC TGA AT	GCT GGA GTT GCT GGA AGC
ACTB	*Homo sapiens Actin Beta*	NM_001101.3	CCA AAC GCG AGA AGA TGA	CCA GAG GCG TAC AGG GAT AG
ACTA2	*Homo sapiens actin, alpha 2, smooth muscle, aorta*	NM_001141945.2	GTG ATC ACC ATC GGA AAT GAA	TCA TGA TGC TGT TGT AGG TGG T
ALDH1A2	*Homo sapiens aldehyde dehydrogenase 1 family member A2*	NM_170697.3, NM_170696.3	ATC AAC AAG GCC CTC ACA GT	TCT GGG CAT TTA AGG CAT TG
CDH5	*Homo sapiens cadherin 5*	NM_001795.4	AAG CCT CTG ATT GGC ACA GT	CTG GCC CTT GTC ACT GGT
COL1A1	*Homo sapiens collagen type I alpha 1 chain*	NM_000088.3	CAA GAG TGG TGA TCG TGG TG	GCC TGT CTC ACC CTT GTC A
COL3A1	*Homo sapiens collagen type III alpha 1 chain*	NM_000090.3	ACA TCG AGG ATT CCC TGG TA	GCT GGA GTT GCT GGA AGC
COL6A2	*Homo sapiens collagen type VI alpha 2 chain*	NM_001849.3, NM_058174.2, NM_058175.2	AGA ACG GGA CCG ATG GAC	CCT GGA CTC CCT GCT TCC
CXCL3	*Homo sapiens C-X-C motif chemokine ligand 3*	NM_002090.2	AAA TCA TCG AAA AGA TAC TGA ACA AG	GGT AAG GGC AGG GAC CAC
CXCL6	*Homo sapiens C-X-C motif chemokine ligand 6*	NM_002993.3	GTC CTT CGG GCT CCT TGT	CAG CAC AGC AGA GAC AGG AC
DDR2	*Homo sapiens discoidin domain receptor tyrosine kinase 2*	NM_001014796.3, NM_001014796.3, NM_001354983.2	TAT GGC ACC CAC AAC CTA TG	TGG CCA GGA GGA TAA AGA TG
ENG (CD105)	*Homo sapiens endoglin (ENG)*	NM_001114753.2, NM_000118.3, NM_001278138.1	AAT GCC ATC CTT GAA GTC CA	GTG CCA TTT TGC TTG GAT G
FBLN2	*Homo sapiens fibulin 2*	NM_001998.3, NM_001165035.2, NM_001004019.2	CCC CAG AAG TAG CCC TAG A	CAG GCA CTC GTC ATT GTC A
IL1B	*Homo sapiens interleukin 1 beta*	NM_000576.2	TAC CTG TCC TGC GTG TTG AA	TCT TTG GGT AAT TTT TGG GAT CT
NKX2.5	*Homo sapiens NK2 homeobox 5*	NM_004387.3	TTC TAT CCA CGT GCC TAC AGC	CTG TCT TCT CCA GCT CCA CC
PECAM1 (CD31)	*Homo sapiens platelet and endothelial cell adhesion molecule 1 (PECAM1)*	NM_000442.4	ATG CCG TGG AAA GCA GAT AC	CTG TTC TTC TCG GAA CAT GGA
PDGFRA	*Homo sapiens platelet-derived growth factor receptor alpha*	NM_006206.6	CCA CCT GAG TGA GAT TGT GG	TCT TCA GGAAGT CCA GGT GAA
PDGFRB	*Homo sapiens platelet-derived growth factor receptor beta*	NM_002609.3	GTG CTG GGA AGA GAA GTT TGA	TCA TCC ACC TGC TGG TAC TTC

S100A4	*Homo sapiens S100 calcium binding protein A4*	NM_002961.2, NM_019554.2	GCT CAA CAA GTC AGA ACT AAA GGA G	GCA GCT TCA TCT GTC CTT TTC
TBX5	*Homo sapiens T-box 5*	NM_000192.3	TGA TCA TAA CCA AGG CTG GA	GAT TAA GGC CCG TCA CCT TC
TIMP3	*Homo sapiens TIMP metallopeptidase inhibitor 3*	NM_000362.4	GCT GGA GGT CAA CAA GTA CCA	CAC AGC CCC GTG TAC ATC T
TGFB1	*Homo sapiens transforming growth factor beta 1*	NM_000660.6	ACT ACT ACG CCA AGG AGG TCA C	TGC TTG AAC TTG TCA TAG ATT TCG
TAGLN	*Homo sapiens transgelin*	NM_001001522.1, NM_003186.3	CAG ACT GTT GAC CTC TTT GAA GG	GCC CAT CAT TCT TGG TCA CT
THY1 (CD90)	*Thy-1 Cell Surface Antigen*	NM_006288.5, NM_001372050.1, NM_001311162.2, NM_001311160.2	CAG AAC GTC ACA GTG CTC AGA	GAG GAG GGA GAG GGA GAG C
Actb	*Rattus norvegicus actin beta*	NM_031144	CCA ACC GTG AAA AGA TGA CC	ACC AGA GGC ATA CAG GGA CA
Fas	*Rattus norvegicus Fas cell surface death receptor*	NM_139194.2	TGC GCC TTC TGT GAT GAA	CTT TGC ACC TGC ACT TGG T
Bax	*Rattus norvegicus BCL2 associated X, apoptosis regulator (Bax)*	NM_017059	CGA GCT GAT CAG AAC CAT CA	GGG GTC CCG AAG TAG GAA
Tnnt2	*Mus musculus troponin T2, cardiac type (Tnnt2)*	010508057/58	TTC GAC CTG CAG GAA AAG TT	CTT CCC ACG AGT TTT GGA GA

Abbreviations: qRT-PCR: semi-quantitative real-time polymerase chain reaction

Supplemental Table 15: Primer sets for microRNA qRT-PCR

**microRNA**	**Company**	**Catalogue number**
Hsa-miR-132-3p	Exiqon, now Qiagen, Hilden, Germany	YP00206035
Hsa-miR-146a-5p	Exiqon, now Qiagen, Hilden, Germany	YP00204688
Hsa-miR-21	Exiqon, now Qiagen, Hilden, Germany	YP00204230
Hsa-miR-423-3p	Exiqon, now Qiagen, Hilden, Germany	YP00204488

Supplemental Table 16: 1st antibodies

Protein target	Dilution	Conju-gated	Application	Company (catalogue number)
**CD90**	1:11	PE-Cy5	Flow cytometry	eBioscience, Inc., San Diego, CA, (#15-0909)
**CD105**	1:11	APC	Flow cytometry	eBioscience, Inc., San Diego, CA, (#17-1057)
**CD31**	1:11	PE-Cy7	Flow cytometry	eBioscience, Inc., San Diego, CA, (#25-0319-42)
**CD45**	1:11	FITC	Flow cytometry	eBioscience, Inc., San Diego, CA, (#11-94-59)
**DDR2**	1:20	-	ICC	LSBio, Seattle, WA, (#LS-C99151/64099)
**CD90**	1:50	-	ICC	antibodies-online GmbH, Aachen, Germany (#ABIN1724884)
**CD31**	1:100	-	ICC	Abcam, Cambridge, UK, (#ab28364)
**α-SMA**	1:100	-	ICC	Abcam, Cambridge, UK, (#ab5694)
**TropT**	1:50	-	ICC	Abcam, Cambridge, UK, (#ab125266)
**α-Actinin**	1:200	-	ICC	Abcam, Cambridge, UK, (#ab9465)
**α-MHC**	1:100	-	ICC	Novus Biologicals, R&D Systems, Minneapolis, MN, (#MAB4470)
**Tnni3**	1:100	-	ICC	Abcam, Cambridge, UK, (#ab200080)
**Cx43**	1:100	-	ICC	Sigma-Aldrich, Merck Millipore, Merck KGaA, Darmstadt, Germany, (#MAB3068)

Abbreviations: ICC: immunocytochemistry

Supplemental Table 17: 2nd antibodies

Antibodies	Dilution/ Application	Fluorescent dye	Company (catalogue number)
**Goat anti-mouse IgG H&L**	1:500 (ICC)	Alexa Fluor® 555	Abcam, Cambridge, UK, (#ab150114)
**Goat anti-mouse IgG H&L**	1:500 (ICC), 1:2.000 (FACS)	Alexa Fluor® 488	Abcam, Cambridge, UK, (#ab150113)
**Goat anti-rabbit IgG H&L**	1:500 (ICC)	Alexa Fluor® 555	Abcam, Cambridge, UK, (#ab150078)
**Goat anti-rabbit IgG H&L**	1:500 (ICC), 1:2.000 (FACS)	Alexa Fluor® 488	Abcam, Cambridge, UK, (#ab150077)

Abbreviations: ICC: immunocytochemistry; FACS: fluorescent activated cell analysis

Supplemental Table 18: Cell stock concentrations and targeted cell recovery

Sample No.	Cell stock concentration (cells/µL)	Targeted cell recovery
1 (EC)	100	1000
2 (CF)	100	1000
3 (SMC)	100	1000
4 (CDC adult)	700	2000
5 (CDC infant)	800	2000

Supplemental Table 19: Parameters used for PCA and UMAP

Seurat objects	Total number of PCs to computed and stored by RunPCA function (npcs)	Dimensions to use as input features by RunUMAP function (dims)
Merged CCA corrected-object of CDCs_adult, CFs, SMCs, ECs	25	20
Merged object of CDCs_adult and CDCs_Infant	50	25

Abbreviations: PCA: principal component analysis, UMAP: uniform manifold approximation and projection, PCs: principal components, CCA: canonical correlation analysis, CDCs: cardiosphere-derived cells, CFs: cardiac fibroblasts, ECs: endothelial cells, SMCs: smooth muscle cells.

4. Supplemental Materials and Methods Section

Patient-derived samples

All human primary cells (CDCs, AFs, CFs, SMCs, ECs) were cultivated from biopsies derived from adipose, atrial or vessel tissue from patients undergoing heart surgery (age: 5 days to 76 years) (Suppl. Table 1, Suppl. Table 2). All patients had signed an informed consent. In the case of infants, informed consents were signed by their parents or legal guardians. The local ethics committee of the Technical University of Munich Medical School controlled and approved the study (project number 570/16S). Tissue sampling and the establishment of the cardiovascular biobank was also approved by the local ethics committee of the Technical University of Munich Medical School (project number 5943/13). All experimental procedures were performed in accordance with the principles outlined in the Declaration of Helsinki.

Animals

Murine cardiac fibroblasts were generated from adult mouse hearts. Transgenic mice were housed in an accredited facility in compliance with the European Community Directive related to laboratory animal protection (2010/63/EU). For extraction of adult murine ventricles, mice were anesthetized with isoflurane (2-chloro-2-(difluoromethoxy)-1,1,1-trifluoroethane, Baxter, Deerfield, IL) and then euthanized by cervical dislocation. For this experiment, transgenic Nkx2.5 cardiac enhancer eGFP (NkxCE GFP) mice, characterized previously by Wu *et al.* [30], were used. Neonatal rat cardiomyocytes (NRCMs) were isolated from 0-1 day old Sprague Dawley rats (non-transgenic) after decapitation. The rats were housed in an accredited facility in compliance with the European Community Directive related to laboratory animal protection (2010/63/EU).
All animal sacrifices for harvesting organs were approved by the relevant authority “Regierung von Oberbayern” [Regional Government of Upper Bavaria], German TierSchG (Animal protection law). All animal experiments (organ extractions) were performed in accordance with the European guidelines and regulations for animal care and handling (Directive 2010/63/EU).

Generation and cultivation of human cardiosphere-derived cells (CDCs)

Cardiosphere-derived cells (CDCs) were isolated from fresh human atrial appendage tissue according to Messina et al. [20]. In brief: Myocardial tissue was rinsed with 1x D-PBS (Biochrom, Merck Millipore, Merck KGaA, Darmstadt, Germany) several times. The biopsy was then cut into

1-2 mm^3 sized pieces and again washed with 1x D-PBS. The tissue was digested thrice, each time for 5 min at 37 °C with a COG digestion solution consisting of 80% (v/v) 0.25% trypsin-EDTA (Life Technologies™, Thermo Fisher Scientific, Waltram, MA), 10% collagenase IV solution (Collagenase, Type IV, Life Technologies™, Thermo Fisher Scientific) and 10% 1x D-PBS. Each digestion step consisted of short vortexing, rotating the tubes at 37°C inside a hybridization incubator (GENEO BioTechProducts GmbH, Hamburg, Germany) for 5 min, followed by a short centrifugation step (pulse mode in the centrifuge 5417R, Eppendorf, Hamburg, Germany) and a subsequent replacement of the COG digestion solution. After the last digestion step, the tissue fragments were plated into a 24-well plate (Greiner Bio-One International GmbH, Frickenhausen, Germany) in complete explant medium (CEM) consisting of 20% FBS (Thermo Fisher Scientific), 100 units/mL penicillin, 100 µg/mL streptomycin (PanReac AppliChem, AppliChem GmbH, Darmstadt, Germany), 0.1 mmol/L 2-mercaptoethanol (Gibco® by Life Technologies™, Thermo Fisher Scientific) in Isocove's basal medium with stable glutamine (Biochrom). When the outgrowing cell layer reached confluence, the sample was considered to be ready for harvest. The growing period of the explant outgrowth usually lasted approximately two weeks and the culture medium was changed every other day. Only the phase bright cells should be harvested in the next step to build spheres. To do so, several washing steps were performed and medium and 1x D-PBS from all steps was collected and pooled: First, the conditioned medium was collected, then the cells were washed with 1x D-PBS, a second wash was performed with 0.05% trypsin-EDTA solution (1x D-PBS dilution of 0.25%) for 1 min at RT and finally one more wash with cardiosphere-growing medium (CGM) was done (CGM: 100 units/mL penicillin g, 100 µg/mL streptomycin , 3.5% FBS, 0.1 mmol/L 2-mercaptoethanol, 20 ng/mL recombinant human FGF basic (Peprotech, Rocky Hill, NJ), 40 ng/mL cardiotrophin-1 (Peprotec, Rocky Hill, NJ), 2% B-27 (Invitrogen™ by Thermo Fisher Scientific), 40 nmol/L thrombin (Sigma-Aldrich, Merck Millipore, Merck KGaA), 250 ng/mL recombinant human EGF (R&D System, Minneapolis, MN) in 65% DMEM/Ham's F12, with +L-glutamine (Biochrom) and 35% Isocove's basal medium with stable glutamine. After the harvested cells were centrifuged (300 x g, 5 min, RT), they were plated on poly-D-lysine- (Sigma-Aldrich, Merck Millipore, Merck KGaA,) coated 4-well plates (VWR International, Radnor, PA) at a concentration of $8×10^4$/mL in CGM. Harvesting of cardiosphere-forming cells was performed thrice, with intervals of one week.

After about 5 days, the three-dimensional cardiospheres were re-plated on fibronectin- (Sigma-Aldrich, Merck Millipore, Merck KGaA) coated 12-well plates yielding one-dimensional culture of cardiosphere-derived cells (CDCs). CDCs were cultured in complete explant medium (CEM) changing the medium every other day. Passaging was performed with 0.25% trypsin-EDTA.

Generation and cultivation of human adipose tissue-derived fibroblasts (AFs) and cardiac fibroblasts (CFs)

AFs and CFs were isolated from fresh human adipose tissue or human atrial appendage tissue, respectively, according to a previously published protocol [10]. The tissue samples were minced manually and incubated with a 0.2% collagenase II solution (Life Technologies™, Thermo Fisher Scientific), w/v in 1x D-PBS, supplemented with 100 U/mL penicillin und 100 µg/mL streptomycin, while rotating inside a hybridization incubator (GENEO BioTechProducts GmbH) at 37°C for 2.5 h. The incubation time was extended for large tissue samples to obtain optimal

digestion. In case tissue pieces remained in the digestion solution after the incubation period, the solution was filtered through a 70 µM filter (Greiner Bio-One International GmbH).
After digestion, the solution was centrifuged at 300 x g for 5 min at RT. The pelleted cells were plated in fibroblast medium consisting of 10% FBS, 100 units/mL penicillin G, 100 µg/mL streptomycin, 1% sodium pyruvate (Thermo Fisher Scientific) in DMEM high glucose with stable glutamine (Biochrom) on 12-well plates. The medium was changed twice a week and the cells were passaged with 0.25% trypsin-EDTA.
Murine cardiac fibroblasts (mCFs) were isolated with the same protocol from ventricles of adult transgenic Nkx2.5CE-GFP mice.

Generation and cultivation of human endothelial cells (ECs) and smooth muscle cells (SMCs)

ECs and SMCs were isolated from fresh human vessels (veins or internal mammary arteries) according to a previously published protocol [10].
In brief, residual blood was washed off the inner parts of the vessels with sterile 1x D-PBS using a 10 mL sterile injection syringe (B. Braun Melsungen AG, Melsungen, Germany). Then, after the vessel was closed on one side using titanium ligating clips (Teleflex Inc., Wayne, Pennsylvania) under sterile conditions, 0.2% collagenase II solution w/v in 1x D-PBS, supplemented with 100 U/mL penicillin und 100 µg/mL streptomycin was injected into the interior of the vessel followed by closure of the vessel on the other side. The vessel was subsequently placed into a 15 mL falcon tube filled with 1x D-PBS which was placed into a water bath (Lauda Aqualine AL 12, LAUDA-Brinkmann) for 30 min at 37°C. After incubation, ECs were washed off the inner part of the vessel by flushing it with 1x D-PBS. The cell suspension was centrifuged at 300 x g for 5 min and the cells were plated on a 12-well culture plate in EC medium (Promocell GmbH, Heidelberg, Germany) supplemented with all provided supplements and 100 units/mL penicillin G, 100 µg/mL streptomycin.
For SMC generation, the remaining vessel was cut into 1-2 mm^2 pieces. They were put on a 6-well plate (Greiner Bio-One International GmbH), the inner side of the vessel facing the bottom of the well to allow smooth muscle cell outgrowth. After 2-3 h without medium, the vessel pieces attached to the culture plate and SMC medium (Promocell GmbH) was added.
For both, ECs and SMCs, the medium was changed every other day and the cells were passaged with 0.125% trypsin-EDTA (1:2 dilution of the commercial 0.25% trypsin-EDTA with 1x D-PBS).

Generation and cultivation of sphere-derived cells out of human cardiac fibroblasts (CFSPhs)

Human CFs (p0) were passaged by trypsin-EDTA to 12-well plates (Greiner Bio-One International GmbH) at a 1:3 ratio. When grown to confluence, the cells were trypsinized and re-plated to poly-D-lysine- (Sigma-Aldrich, Merck Millipore, Merck KGaA) coated 4-well plates (VWR International) at a concentration of 8×10^4 cells/mL in a cardiosphere-growing medium (CGM, see “Generation and cultivation of human cardiosphere-derived cells (CDCs)”). After about 5 days, the three-dimensional cardiospheres were re-plated to fibronectin- (Sigma-Aldrich, Merck Millipore, Merck KGaA) coated 12-well plates in CEM (see “Generation and cultivation of human cardiosphere-derived cells (CDCs)”). In most cases, the spheres did not attach to the 4- well plates and could be easily collected by taking off the supernatant. Otherwise, they were detached from

the 4-well plates by gently rinsing the cell layer with a small volume of CEM. Analogically to CDCs, cells grew out of the spheres after several days. These cells were called cardiac fibroblast sphere-derived cells (CFSPhs). Analogically to CDCs, the medium was changed every other day and the cells were passaged with 0.25% trypsin-EDTA.

Cultivation of human induced pluripotent stem cells (hiPSCs)

We used an established human induced pluripotent stem cell (hiPSC) line for this study. Its generation by the reprogramming of fibroblasts of a healthy control individual and its characterization have been previously published [26]. HiPSCs were cultured in TeSR™-E8™ medium (StemCell™ Technologies, Cologne, Germany) on 6-well culture plates (Greiner Bio-One International GmbH) coated with Corning® Matrigel® hESC-qualified matrix (Corning, Tewksbury, MA), according to manufacturer's instructions. Passaging was performed with ReLeSR™ (StemCell™ Technologies) according to manufacturer's instructions.

Cardiac differentiation of hiPSCs

HiPSCs underwent a previously published cardiac differentiation protocol, modulating the Wnt signaling pathway with several small molecules [6]. Two days before starting differentiation, hiPSCs were passaged by 0.5 mM EDTA in 1x D-PBS (Gibco® by Life Technologies™, Thermo Fisher Scientific) to matrigel-coated 24-well plates to achieve a cellular monolayer. After two days, hiPSCs grew to a dense monolayer and cardiac differentiation was started (day 0) by adding 3.5 µM CHIR99021 (LC Laboratories, Woburn, MA,) in CDM3 differentiation medium, consisting of 500 µg/mL recombinant human serum albumin (Sigma-Aldrich, Merck Millipore, Merck KGaA, Darmstadt, Germany), 213 µg/mL L-ascorbic acid 2-phosphate (Sigma-Aldrich, Merck Millipore, Merck KGaA) in Roswell Park Memorial Institute (RPMI) 1640 medium (Biochrom). At day 2 (D2), the medium was changed to CDM3 containing 2 µM Wnt-C59 (Sellekchem, Houston, TX). From D4, the medium was changed every other day (pure CDM3). Usually, first beating cells were observed by D8 and by D14, large parts of the wells contained beating areas. RNA was isolated at D6 and D8 of differentiation, considering most hiPSCs to be in a cardiac progenitor state or early cardiomyocyte state, respectively.

Isolation of neonatal rat cardiomyocytes (NRCMs)

NRCM isolation procedure was performed according to previously published standard procedures [23]. In brief: 0–1-day old Sprague Dawley rats were decapitated after disinfection of their neck area. Whole hearts were then explanted and digested with collagenase type II (Worthington) and pancreatin (Sigma Aldrich) in CBFHH buffer (120 mM NaCl, 5 mM KCl, 0.8 mM $MgSO_4$, 0.5 mM KH_2PO_4, 0.3 mM Na_2HPO_4, 20 mM HEPES, 5.6 mM glucose, pH 7.3 including penicillin/streptomycin) at 37 °C for 1 h. In intervals of 10 min, digestion solution containing dissociated tissue cells was collected in FCS (Sigma Aldrich) and fresh enzymatic solution was added to the undigested heart tissue pieces (5 times). Thereafter, the collected cells were centrifuged at 50 × g for 5 min and resuspended in MEM medium containing 5% FCS. After undigested tissue was filtered out with a 40 µm strainer (BD), the cells were pre-plated at 37°C and 1% CO_2 for 75 min on 10 cm cell culture dishes (Nunc) to adhere cardiac fibroblasts. Cell culture supernatant containing NRCMs was then collected, centrifuged at 100 x g for 2 min and plated on 96-well plates (Greiner Bio-One International GmbH) coated with sterile 0.1% gelatin

in bi-distilled water (w/v, Sigma-Aldrich, Merck Millipore, Merck KgaA) with NRCM medium consisting of 5% horse serum (Life Technologies™, Thermo Fisher Scientific), 100 units/mL penicillin g, 100 µg/mL streptomycin in DMEM/Ham´s F12 (Biochrom) at a concentration of 4×10^4 cells /96-well.

RNA isolation and purification

Total RNA was isolated from primary cells (CDCs, CFs, AFs, ECs, SMCs) at passage 0 and from iPSCs on D6 and D8 of cardiac differentiation using the peqGOLD Total RNA Kit (S-Line), (Peqlab, VWR International) according to manufacturer's instructions. The cells were lysed by denaturation with the provided RNA lysis buffer and lysates were stored at -20°C until RNA purification procedure. DNA digestion with the peqGOLD DNase I Digest Kit (Peqlab) was performed on the column according to manufacturer's instructions.

To analyze microRNA content in extracellular vesicles (EVs) (isolation protocol see below), total RNA was isolated using the SeraMir Exosome RNA Purification Column Kit (System Biosciences, Palo Alto, CA) according to manufacturer's instructions. In brief, the pellet in the final step of EV isolation was directly resuspended in 350 µL lysis buffer provided in the kit. Exosomal RNA was purified with RNA-binding spin columns and finally eluted with 30 µL elution buffer.

Total RNA concentrations and quality were determined by a Nanodrop 2000c spectrophotometer (Thermo Fisher Scientific) and RNA was then stored at -80°C (long term).

Reverse transcription for gene expression analysis

For gene expression analysis by qRT-PCR, 100 ng of total RNA were reverse transcribed with the M-MLV Reverse Transcriptase Kit (Invitrogen™, Thermo Fisher Scientific) and random hexamer oligonucleotides (Invitrogen™, Thermo Fisher Scientific) in a Thermocycler C1000 (BioRad Laboratories GmbH, Hercules, CA) according to manufacturer's instructions. The following conditions were used: 5 min at 65°C, 2 min at 37°C, 10 min at 25°C, 50 min at 37°C and 15 min at 75°C. cDNA was stored at -20°C.

Reverse transcription for microRNA analysis

For microRNA expression analysis by qRT-PCR, 100 ng of total RNA was reverse transcribed with miRCURY LNA™ RT Kit (Qiagen, Hilden, Germany) according to manufacturer's instructions in a C1000 Thermal Cycler (Bio-Rad Laboratories GmbH): 42 min at 60 °C, 5 min at 95 °C. cDNA was stored at -20°C.

Quantitative real-time PCR (qRT-PCR)

Semi-quantitative real time PCR (qRT-PCR) for the evaluation of gene expression was performed using the Power SYBR® Green PCR Master Mix kit (Applied Biosystems by Life Technologies™, Thermo Fisher Scientific) on a Quant Studio 3 (QS3) instrument (Applied Biosystems by Thermo Fisher Scientific). Gene-specific primer sets were purchased at ELLA Biotech, (Munich, Germany) (Suppl. Table 14). Cycling conditions: 2 min at 50°C and 10 min at 95°C followed by 40 cycles with 15 s at 95°C and 60 s at 60°C. Subsequently, a melting curve analysis was performed by applying temperature modes of 15 s at 95°C, 1 min at 60°C and 1 s at

95°C. Gene expression was then calculated using arbitrary units (AU) and each sample was normalized to the housekeeping gene *β-ACTIN (ACTB) or β-actin (Actb).*

To assess relative expression of microRNA, qRT-PCR was performed on a QS3 using the miRCURY LNA™ microRNA PCR ExiLENT SYBR® green master mix (Exiqon, now Qiagen) according to manufacturer's instructions and microRNA primer sets (Exiqon, Suppl. Table 15). After an initial activation step of 2 min at 95°C, amplification was performed for 40 cycles (10 s at 95°C followed by 1 min at 56°C). The melting curve analysis was performed in the same manner as described above. Quantification of microRNA expression was performed using the $2^{-\Delta\Delta CT}$-method by normalization of each sample to its expression of microRNA 423-3p and the mean of all samples.

Flow cytometry

Flow cytometry analysis of cell surface markers of CDCs, AFs, CFs, SMCs and ECs was performed.

The cells were grown to passages 2 or 3 to have enough cells for staining. For analyzing the surface markers CD90, CD45, CD105, and CD31, the cells were detached using 0.25% trypsin-EDTA. Then, the cells were centrifuged (300 x g, 5 min, RT) and resuspended in FACS buffer, consisting of 0.5% (w/v) bovine serum albumin (Sigma-Aldrich, Merck Millipore, Merck KGaA) and 2 mM EDTA (EDTA, Carl Roth GmbH + Co. KG, Karlsruhe, Germany) in 1x D-PBS. The cells to be co-stained against CD90/CD45 or against CD105/CD31 were incubated with fluorophore-labeled antibodies at a 1:11 dilution for 30 min on ice in the dark (see information on antibodies in Suppl. Table 16). Subsequently, the cells were washed twice with FACS buffer and then resuspended in fresh FACS buffer. All samples were then filtered through 30 µm filters (30 µm Syringe Filcons, BD Biosciences) and incubated with DAPI solution (final conc. 1 µg/mL, 4',6-diamidino-2-phenylindole, Biolegend, San Diego, CA) to stain dead cells. Gating strategy: After filtering out small particles (e.g. cell debris) by setting the gates on the majority of bigger particles in the FSC (forward scatter) vs. SSC (side scatter) window, DAPI negative cells were chosen for analysis. Gates for the according channels (PE-Cy5/FITC or PE-Cy7/APC) were set depending on the respective negative controls.

Extracellular vesicles (EVs) were analyzed for the surface markers CD63 and CD81 with the Exo-Flow Exosome Capture Kit (System Biosciences) according to manufacturer's instructions. In brief, magnetic beads were prepared by washing them twice with the Bead Wash Buffer. Biotinylated antibodies (either CD81 or CD63) were bound to the magnetic beads, which are coated with a streptavidin layer for 2 h on ice. Residual antibody was washed off with the Bead Wash Buffer (3 x). Isolated EVs from the conditioned medium of one T75 flask were diluted to a total volume of 100 µL in 1x D-PBS (PAN-Biotech GmbH, Aidenbach, Germany) and incubated with the stained beads overnight at 4°C. The negative control was incubated with serum-free IMDM medium consisting of 10 mg/mL bovine serum albumin, 100 units/mL penicillin g and 100 µg/mL streptomycin, 0.1 mmol/L 2-mercaptoethanol in Isocove's basal medium with stable glutamine. To detect the beads coupled to EVs by flow cytometry, the samples were incubated with Exo-FITC exosome stain for 2 h on ice. Residual dye solution was washed off with Bead Wash Buffer (3 x). During flow cytometry analysis, the first gate was set on the majority of bead

singlets, which could be distinguished from bead multimers in the FSC vs. SSC window. The second gate was made according to the FITC signal of the negative control.

Flow cytometry analysis was performed on a FACS BD LSR Fortessa (Becton, Dickinson and Company, BD Biosciences) with the software BD FACSDiva^{TM} version 8.0.1, (Becton, Dickinson and Company, BD Bioscience). The FCS files were analyzed with FlowJo Version version 7.6.5 (FlowJow LLC, Ashland, OR).

Immunocytochemistry (ICC)

To assess typical cell markers on protein level ICC of AFs, CDCs, CFs, SMCs and ECs was performed according to the following protocol: The cells were fixed with ice-cold methanol (Sigma-Aldrich, Merck Millipore, Merck KGaA) at -20°C for 15 min.

NRCMs were fixed with 4% (v/v) para-formaldehyde (Carl Roth GmbH + Co. KG), 4% (w/v) sucrose (Sigma-Aldrich, Merck Millipore, Merck KGaA) in 1x D-PBS.

For the detection of intracellular markers (α-SMA, α-Actinin, TropT, αMHC, Tnni3), the cells were permeabilized with 0.25% (v/v) Triton-X-100 (Sigma-Aldrich, Merck Millipore, Merck KGaA) in 1x D-PBS for 15 min and subsequent washes were performed with 0.1% (v/v) Triton-X-100 in 1x D-PBS (0.1% PBS-T). Unspecific binding was blocked with 5% (v/v) normal goat serum (Abcam, Cambridge, UK) in 1x D-PBS/ 0.1% PBS-T for 30 min. Primary antibodies were diluted in 1x D-PBS/ 0.1% PBS-T (concentrations shown in Suppl. Table 16) and incubated overnight at 4°C. After washing with 1x D-PBS/ 0.1% PBS-T (2 x), the cells were incubated with secondary antibodies (dilutions in Suppl. Table 17) for 1 h at RT in the dark. After washing twice with 1x D-PBS/ 0.1% PBS-T and one time with Aq. bidest. slides were air-dried, mounted with Mounting Medium with DAPI (Abcam), sealed with cover slips (Menzel^{TM} Microscope Coverslips 24 x 60 mm, Thermo Fisher Scientific) and evaluated under a fluorescence microscope (Axiovert 200M, Zeiss, Oberkochen, Germany) equipped with a camera (AxioCam MRm; Zeiss) and using the software Axio Vision Rel. version 4.8.2 (Zeiss) for taking pictures.

Preparation of cell samples (CDCs, CFs, ECs, SMCs) for single-cell RNA sequencing (sc-RNAseq)

The cells (CDCs, CFs, ECs, SMCs; see Suppl. Table 5) were grown in T75 flasks to 70-80% confluence. After washing with 1x D-PBS, the cells were detached with 0.125% trypsin-EDTA (1:2 dilution of 0.25% trypsin-EDTA with 1x D-PBS). Trypsin was stopped with the triple amount of the respective cell culture medium and the cells were centrifuged at 100-300 x g for 10 min at 4°C. The cell pellets were resuspended in the respective culture medium and filtered through 30 µm pre-separation filters (Miltenyi Biotech, Bergisch Gladbach, Germany). Cells were then counted with an automated cell counter (Countess II FL, Thermo Fisher Scientific) after staining the live and dead cells with fluorescent dyes (Ready Probe Viablity Imaging Kit Blue/Green (Thermo Fisher Scientific) according to manufacturer’s instructions (dilution of 1:10; 15 min incubation). Only cell preparations with > 95% positive cells in the DAPI channel (live and dead cells) and < 10% positive cells in the GFP channel (dead cells) were used for cDNA library preparation.

Cell samples 1-3 (CFs, ECs, SMCs; Suppl. Table 5) were processed according to the library preparation protocol Chromium Single Cell 3’ Reagent Kits v2 (10x Genomics, Pleasanton, CA),

whereas samples 4 and 5 (CDCs, Suppl. Table 5) were processed according to the protocol of Chromium Next GEM Single Cell 3´Reagent Kits v3.1 (10x Genomics). Cell stock concentrations and the targeted cell recovery are depicted in Suppl. Tab. 18. Prior to loading the cells on the chip, the cells were mixed with nuclease-free water and the master mix according to the corresponding protocol's instructions.

As indicated in the corresponding protocols, Chip A Single Cell (10x Genomics) was used for samples 1-4 and Chromium Next GEM Chip G for samples 5 and 6. The empty wells of the chips were filled with 50% glycerol solution (Ricca Chemical Company, Arlington, TX). Both chips were run on the Chromium Controller with the firmware version fitting to the chip. Immediately thereafter, GEMs were transferred to a tube strip and GEM-RT PCR was performed on a Veriti™ Dx 96-well Fast Thermal Cycler (Applied Biosystems by Thermo Fisher Scientific): 45 min at 53°C, 5 min at 85°C and holding at 4°C. The lid temperature was set to 53°C.

Post GEM-RT cleanup and cDNA amplification were performed according to the corresponding protocols. As the targeted cell recovery was 1000-2000 cells, a total cycle number of 12-14 cycles was chosen, as recommended. Thereafter, one-sided size selection was performed as cDNA was cleaned up with 0.6 x SPRIselect reagent (Beckman Coulter GmbH, Krefeld, Germany). Quality check was done with an Agilent Bioanalyzer 2100 (Agilent, Santa Clara, CA) using the Agilent High Sensitivity DNA Kit (Agilent).

Next, cDNA amplicon size was optimized by fragmentation and size selection. As recommended in the protocol, 25% of the generated cDNA was used to achieve optimal complexity. So different samples could be distinguished from one another when pooled together for sequencing, sample indices were annealed to each DNA fragment by a 12-cycle PCR using a Chromium i7 Sample Index plate (10x Genomics) of the Chromium i7 Multiplex Kit (10x Genomics).

Preparation of right atrial biopsy samples (RA-1, RA-2) for single-nuclei RNA sequencing (sn-RNAseq)

Two previously published samples of sn-RNAseq [15] from right human atria were included into the analysis (Suppl. Table 9). As already published, samples were collected from two patients with no history of coronary artery disease at the German Heart Center Munich and directly snap-frozen in liquid nitrogen in the operating room. Tissue samples were minced and nuclei extracted in lysis buffer containing 5 mM CaCl2, 3 mM magnesium actetate, 2 mM EDTA, 0.5 mM EGTA, 10 mM Tris, 0.2%, Triton X-100, protease inhibitors, DTT and RNAse inhibitors. Nuclei were purified by gradient centrifugation with 1 M sucrose and then resuspended in PBS. After staining with Draq7 (Thermo Fisher Scientific), samples were further purified by fluorescence-activated nuclei sorting (FANS). Nuclei were counted under the microscope and diluted for subsequent 10x Genomics Chromium™ Next GEM Single Cell 3' Solution v3. Barcoding, cDNA amplification and gene expression library construction were done according to the manufacturer's recommendations (10x Genomics, described in detail above).

Preparation of right atrial biopsy samples (RA-3, RA-4) for single-cell RNA sequencing (sc-RNAseq)

Left atrial appendage biopsies were obtained during heart surgery in Ringer's solution (Suppl. Table 9). Transportation from operating room to the laboratory was performed on ice. Tissue was

washed twice with ice-cold 1x D-PBS. Before mincing the biopsy, adipose tissue and white fibers were removed. The remaining tissue was minced to small pieces of approximately 1 mm^3 and incubated in 0.25% trypsin-EDTA at 37°C for 10 min. Then, 14 mL of digestion solution containing 10 mg /mL collagenase II (#17101-015, Life TechnologiesTM, Thermo Fisher Scientific, Waltram, MA) and 20% FBS in 1x D-PBS were added, and the tissue was digested at 37°C for another 80 min. To remove undigested tissue the digest was filtered through a 70 µM filter. Cell suspension was centrifuged (300 x g, 10 min, 4°C) and the pellet was resuspended in 600 µL ice-cold HBSS. 6 mL of a 1:10 dilution of 10x red blood lysis solution (#130-094-183, Miltenyi Biotech, Bergisch Gladbach, Germany) in Aq. bidest were added and cells were incubated at RT for 3 min with continuous inversion. After centrifugation (300 x g, 10 min, RT), the pellet was resuspended in 1000 µL ice-cold HBSS and filtered through a 30 µm filter. Cell suspension was centrifuged again (300 x g, 10 min at 4°C) and the cell pellet was then incubated with 100 µL magnetic beads of the Dead Cell removal Kit (#130-090-101, Miltenyi Biotech, Bergisch Gladbach, Germany). Magnetic separation was performed twice according to manufacturer's instructions. In brief, the living cell fraction (flow through from the magnetic column) after the first separation was collected and centrifuged at 300 x g for 5 min at 4°C. The pellet was incubated a second time with 100 µL of magnetic beads. The second living cell fraction was centrifuged (300 x g for 5 min at 4°C) and resuspended in 1x D-PBS. Analysis of cell viability was performed using Ready Probe Viability Kit as described above. Cells were counted under the microscope and diluted for subsequent 10x Genomics Chromium™ Next GEM Single Cell 3' Solution v3.1. Barcoding, cDNA amplification and gene expression library construction were done according to the manufacturer's recommendations (10x Genomics, described in detail above).

Sequencing of cDNA libraries

Sequencing of samples 1-3 (CF, SMC, EC) was performed at the laboratory of PD Dr. Tim-Matthias Strom (Helmholtz Zentrum Munich) on the HiSeq 3000/4000 instrument (Illumina, San Diego, CA). Sequence length was set to 151 bp with a total count of 250 million reads. Sequencing of samples 4 and 5 (CDC adult, CDC infant) and RA-3 was performed by the Laboratory for Functional Genome Analysis (LAFUGA) at the Gene Center, LMU, Munich on a HiSeq 1500 (Illumina). A 100 bp flow cell was used in a paired-end mode with a total count of 250 million reads. Library sequencing for single-nuclei human right atria (RA-1, RA-2) was done at the EMBL Heidelberg Genomics Core Facility. The sequencing parameters were 28 bp for read1, 8 bp for the index, and 56 bp for informative read2. Sequencing for RA-4 was done at MLL (MLL, Munich) on a NovaSeq (Illumina). A 100 bp flow cell was used in a paired-end mode. About 100 million reads were done for RA-4.

Analysis of sequenced data of sc-RNAseq and snRNAseq (bioinformatics)

The raw data were analyzed with Cell Ranger (version 3.1.0, 10X Genomics Inc., samples 1-3 (CF, SMC, EC): 3' Gene Expression V2 Libraries, samples 4 and 5 (CDC adult, CDC infant): 3' Gene Expression V3 Libraries). The demultiplexed FASTQ files were generated using the Cell Ranger *mkfastq* command. Then, the FASTQ files were aligned to the human genome reference sequence GRCh38. Subsequently, the preliminary data analysis that included alignment, filtering, barcode counting and the UMI quantification for determining gene transcript counts per cell, quality control, clustering and statistical analysis were performed using the Cell Ranger *count*

command and generated files for each sample that contained a barcode table, a feature table and an expression matrix.

These files were further processed with the Seurat v3.1.2 [7, 19, 24] package implemented in R (v 3.5.3.). The individual samples were first cleaned and normalized. Only data from cells with a minimum of 300 detected genes, and from genes expressed in at least 5 cells, were retained. As described (https://satijalab.org/seurat/), filtering of low quality cells such as doublets and cells containing high numbers of mitochondrial genes was performed. Suppl. Table 6 shows the filtering parameters used for individual samples, which were chosen in a way that excludes most of the outliers for each parameter.

For each sample, a Seurat object was created with "CreateSeuratObject" and the formulas "NormalizeData" (normalization.method = LogNormalize, scale.factor = 10,000), "FindVariableFeatures" and "ScaleData" were applied which present the standard workflow for sample preprocessing prior to a principal component analysis (PCA). When several samples were compared, the "merge" formula was utilized prior to the PCA pre-processing workflow.

When comparing samples 1, 2 and 3 to sample 4 (CDC adult), the samples were merged separately into two objects based on the kit version used for GEM preparation. Since the data came from two different batches of sequencing, canonical correlation analysis (CCA) was performed with the formula "RunCCA" [7] to correct for batch effects among samples and experiments. Thereafter, the principal components were identified with "RunPCA" and uniform manifold approximation and projection (UMAP) [4] dimensionality reduction was utilized to project sample populations into two dimensions ("RunUMAP" formula). Suppl. Table 19 indicates the total number of principal components and dimensions, which were used in PCA and UMAP reduction. These settings were chosen after testing of different parameter combinations based on the biological interests of this work. Graph-based, semi-unsupervised clustering was performed using "FindNeighbors" and "FindClusters" formulas (default parameters), following UMAP projection for visualization ("DimPlot" function). To identify marker genes samples and clusters, they were compared for differential expression using the "FindAllMarkers" formula with the Wilcoxon rank-sum test (default settings). All analyses of output data were performed only with genes with an adjusted *p*-value < 0.05 (indicated in the table provided from Seurat as "p_val_adj"). Enriched genes for a sample or clusters were considered if the average natural logarithm of the gene's fold change (avg_logFC) was ≥ 0.25. The data were visualized in Seurat using violin plots ("VlnPlot" function), FeaturePlot ("FeaturePlot"), Clustertrees ("BuildClusterTree" and "PlotClusterTree") and Heat maps ("DoHeatmap") of individual genes.

The identification of gene ontology terms enriched in a group of genes was performed with gene set enrichment analysis (GSEA), which uses gene set collections from the MSigDB26 [25]. Overlaps were calculated for the three-gene ontology (GO) gene sets (biological process, cellular component and molecular function) [3] and the top 50 terms were selected to display. GO terms were considered as significantly enriched if their *p*-value's negative decadic logarithm was higher or equal to 6 (–LOG (*p*-value) ≥ 6).

Heat maps were generated using the website http://heatmapper.ca/expression/ to illustrate the gene expression of genes not expressed in all samples. In case an avg_logFC-value was not displayed for a sample in the FindAllMarkers table (avg_logFC < 0.25 or > -0.25), the value was manually set to 0 in such heat maps.

Venn diagrams were generated using http://bioinformatics.psb.ugent.be/webtools/Venn/ and formatted with the software Inkscape version 0.92.4 (https://inkscape.org/).
Sample similarity calculations (Fig 2E and Fig 4G) were performed according to the method published by Wang *et al.* [29]. FindVariableFeatures was used to identify variable genes in the CCA-corrected merged object (including CDCs, CFs, SMCs and ECs) which were then selected based on their average expression level, and dispersion was restricted to values between 0.01 and 3. The dispersion cutoff was set as greater than 1. Next, the genes were divided into 10 equally sized bins based on mean expression, and 10% of the genes with the lowest variance from each bin were selected, excluding the high and low extremes. The average gene expression profile of a sample was calculated using the selected genes as the center. The Pearson correlation was calculated for each sample to the center and shown as a box plot and density line graph.

Transcripts for previously published single-nuclei human right atria samples (RA-1, RA-2) were mapped to the Human Genome GRCh38-2020-A Reference Package modified to include "pre-mRNA" sequences according to 10xGenomics instructions using Cell Ranger Version 5.0.1. Transcripts for single-cell samples from human right atria (RA-3, RA-4) were mapped to the standard Human Genome GRCh38-2020-A Reference Package.

Sc-RNAseq data from cardiac differentiated individual embryonic stem cells previously published by Mononen et al. [21] was uploaded to the Galaxy web platform [2] and we used the public server at usegalaxy.eu for data processing. Count matrices for the Smart-Seq2 single cell reads mapped on the human genome hg38 with TopHat were generated with FeatureCounts [18] with GRCh38.p12 gene annotation file. Then, matrices from single cells were combined to groups according to the developmental age as detailed in the original publication using the Column Join Text manipulation tool.

Seurat objects for count matrices for samples RA-1, RA-2, RA-3, RA-4, and scRNAseq data from Mononen et al. [21] were created for downstream analyses. After quality filtering, the data was normalized and variable features were detected using FindVariableFeatures [12].
Sc-RNAseq data from adult CDCs (sample 4) and scRNAseq data from Mononen et al. [21] or right atria samples (RA-1, RA-2, RA-3, RA-4), respectively, was integrated using the FastMNN function of the "SeuratWrappers" R-package (Rahul Satija, Andrew Butler, Paul Hoffman and Tim Stuart (2020). SeuratWrappers: Community-Provided Methods and Extensions for the Seurat Object. R package version 0.3.0.). „RunUMAP“, „FindNeighbors“ as well as „FindClusters“ were used for uniform manifold approximation and projection (UMAP) (McInnes, L., Healy, J. & Melville, J. UMAP: Uniform Manifold Approximation and Projection for dimension reduction. Preprint at https://arxiv.org/abs/1802.03426 (2018)), dimension reduction as well as clustering cells into distinct biological identities. FindAllMarkers was used to identify top expressed genes for each cluster and cell clusters were identified based on the expression of known marker genes.

Cell trajectory analysis
For cell trajectory analysis, the R implementation of Monocle3 [8, 22, 27] was used. First, the integrated and clustered Seurat object was converted to a Monocle3-compatible cell data set. This dataset was then processed using the Monocle3 command „cluster_cells“ with standard

parameters, followed by „learn_graph“ with parameter „learn_graph_control“ parameter set to minimal_branch_len=7 and geodesic_distance_ratio=0.5. Defining marker genes for the identified trajectories 1, 2 and 3 were manually selected. „FindClusters“ with parameter „resolution“ set to 1 was used on the integrated Seurat object to identify 22 distinct cell populations. We then correlated top expressed genes identified using „FindAllMarkers“ for the clusters that showed most overlap for the trajectories 1, 2 and 3 with genes that could also be identified with Monocle function „top_markers“ with parameter „group_cells_by=„seurat_clusters" and selecting for the category „specificity“ in the resulting table.

RNA velocity analyses

RNA velocity was calculated on the basis of spliced and unspliced transcript reads according to La Manno et al. [14]. Based on the velocyto pipeline, annotation of spliced and unspliced reads was performed using the Python script velocyto.py on the Cell Ranger „count“ output folder for each single cell sequencing sample.

The integrated and clustered Seurat object was subset to only contain single-cell samples. The .loom files generated by the velocyto pipeline were loaded and added to the seurat object as assays using the R packages „SeuratWrappers“, „SeuratDisk“, „hdf5r“ and „loomR“ with the functions „ReadVelocity“ followed by „CreateAssayObject“

This dataset was used as input for R implementation of velocyto analysis pipeline. The spliced assay was normalized using the SCTransform function with standard parameters. Then, „RunVelocity“ was performed on the object with „reduction=mnn“, „deltaT=1“, „kCells=25“, „fit.quantile=0.02“ paramters selected.

To generate the velocity-plot, „show.velocity.on.embedding.cor“ was employed with the following parameters: emb = Embeddings(object = bm, reduction = "umap"), vel = Tool(object = bm, slot = "RunVelocity"), n = 200, scale = "sqrt", cell.colors = ac(x = cell.colors, alpha = 0.5), cex = 0.8, arrow.scale = 3, show.grid.flow = TRUE, min.grid.cell.mass = 0.5, grid.n = 40, arrow.lwd = 1, do.par = FALSE, cell.border.alpha = 0.1)

Isolation of extracellular vesicles (EVs) from CDCs, CFs, and CFSPhs

CDCs, CFs and CFSPhs were cultivated in five T75 cell culture flasks (Kisker Biotech GmbH & Co. KG, Steinfurt, Germany). When cells reached 80-90% confluence, they were washed thrice with 1x D-PBS to remove all traces of FBS containing medium. Then, the cells were conditioned with serum-free IMDM medium consisting of 10 mg/mL bovine serum albumin, 100 units/mL penicillin g and 100 µg/mL streptomycin, 0.1 mmol/L 2-mercaptoethanol in Isocove’s basal medium with stable glutamine for 7 days to allow sufficient EV release to the medium. After conditioned medium was collected, the cells were detached using 0.25% trypsin-EDTA to a single-cell suspension, which was diluted with 0.2% trypan blue solution (Biochrom) and counted in a Neubauer chamber slide (chamber depth: 0.1 mm, Thomas Scientific, Swedesboro, NJ) to assess the percentage of dead cells.

EVs were isolated from conditioned medium by ExoQuick TC (System Biosciences) according to manufacturer’s protocol. In brief, the conditioned medium was centrifuged at 3000 x g for 15 min to pellet cells and debris. The supernatants were incubated overnight with ExoQuick TC in a volume ratio of 5:1. On the next day, the medium-Exoquick TC solutions were centrifuged (1500 x g, 30 min) followed by removal of most of the supernatant. After a second centrifugation step

(1500 x g, 5 min), any remaining supernatant was removed and the pellet resuspended in 50 µL of 25 mM trehalose (Carl Roth GmbH + Co. KG) in 1x D-PBS (PAN-Biotech GmbH), which was reported to be beneficial for the cryopreservation of EVs [5]. Exosome preparations were stored at -80°C until further use.

Nanoparticle tracking for EV preparations

For measurement of EV concentration and particle size, the nanoparticle tracking instrument ZetaView PMX110 (Particle Metrix, Inning, Germany) was used. Calibration was performed with polystyrene beads of known size and concentration (100 nm NanoStandards, Applied Microspheres, Leusden, the Netherlands), as previously reported [11]. EVs were diluted with 1x D-PBS to a concentration of 100-200 particles per video frame, according to manufacturer's instructions. Each sample was measured at eleven positions with one reading cycle at each position. Camera control settings were set to a sensitivity of 75, a shutter of 50 and a frame rate of 50. Post-acquisition parameters were set to minimal brightness of 20, maximal size of 200, minimal size of 5, and trace length of 15.

Tube formation assay on matrigel with human ECs

Tube formation assay on matrigel was performed according to previously published protocols with certain modifications [13, 28]. Primary human ECs (see paragraph "Generation and cultivation of human endothelial cells (ECs) and smooth muscle cells (SMCs)") were cultured in T75 flasks (< p6). One day prior to the experiment, the complete EC medium was removed and the cells were cultivated with serum-free EC medium consisting of 10.7 mg/mL bovine serum albumin, 100 units/mL penicillin g and 100 µg/mL streptomycin in endothelial cell growth medium 2 without supplements (Promocell GmbH) overnight. 96-well plates were coated with 50 µL undiluted matrigel (Corning) per well according to manufacturer's instructions and incubated at 37°C for 30 min to allow polymerization. ECs were then detached from the T75 cell culture flask by using a 1:1 dilution of accutase (StemCell™ Technologies) with 1x D-PBS at 37°C for 5 min. The detached cells were washed with 1x D-PBS, centrifuged at 300 x g for 10 min, and the cell pellet was resuspended in 200 µL 1x D-PBS. The cells were plated at 2×10^4 cells per well, either with complete EC medium (positive control) or serum-free IMDM medium (negative control) or CDC-/CF-/CFSPh-EV preparations in serum-free IMDM medium (1×10^9 particles per well). After 16 h, pictures of the wells were taken with a ZEISS microscope (Axiovert 200M), using the Carl Zeiss™ Axio Vision Rel. 4.8.2 software. Several pictures were taken per well. Parameters of tube formation were analyzed using the ImageJ angiogenesis analyzer tool written by Gilles Carpentier, 2012, with default settings [1, 9]. The macro is available here: https://imagej.nih.gov/ij/macros/toolsets/Angiogenesis%20Analyzer.txt. The means of the output data for each parameter were calculated and divided by the mean of the negative control (fold change/FC over negative control).

Migration assay (scratch assay) with human ECs and human CFs

Scratch assays were performed according to published protocols with modifications [17].

Primary human ECs (see passage "Generation and cultivation of human endothelial cells (ECs) and smooth muscle cells (SMCs)") or human CFs were cultured in T75 flasks (< p6). On the day of the experiment, ECs and CFs were trypsinized and ECs were seeded at 7.5×10^4 cells per well

and CFs at 5×10^4 cells per well on 96-well ImageLock™ microplates (Essen Biosciences, Hertfordshire, UK). On the next day, the cells were incubated with 20 µg/mL mitomycin C (Sigma-Aldrich, Merck Millipore, Merck KGaA) solution in serum-free endothelial cell medium (see above) or fibroblast medium (see above) for 2 h, to avoid cell proliferation.

For migration assays with ECs, the cells were washed twice with 1x D-PBS and a scratch was performed in the cell layer using WoundMaker™ (Essen Biosciences). Subsequently, ECs were incubated with either ECM (PosCtr), serum-free IMDM medium (NegCtr) or EVs isolated from conditioned medium of CDCs, CFs or CFSPhs of different patients for 48 h. The EVs were diluted in serum-free IMDM medium to a concentration of 1×10^9 particles per 96-well. Pictures of the scratch area were taken every hour using the IncuCyte ZOOM® system camera (Essen Biosciences).

Migration assays with CFs were performed either by scratching the cell layer manually and taking pictures with the Axiovert 200M microscope, or with the automated IncuCyte ZOOM® 96-Well Migration Assay System. For the manual CF scratch assay, the scratch was performed by using a 10 µL pipette tip. Then, CFs were incubated with either fibroblast medium (positive control), serum-free IMDM medium (negative control), or EVs isolated from conditioned medium of CDCs derived from different patients for 48 h. The EVs were diluted in serum-free IMDM medium to a concentration of 2.5×10^9 particles per well of the 96-well plate. Pictures of the scratch area were taken at 0 h, 12 h, 24 h and 48 h after the scratch (Axiovert 200M, Zeiss), using the Carl Zeiss™ Axio Vision Rel. 4.8.2 software.

All images were evaluated using ImageJ [1]. The cell-free area was measured using the function "freehand selections" at 0 h, 8 h, 12 h, 24 h and 48 h after the scratch. The difference of the area at a certain timepoint to the area at time point 0 (Diff (x)) was calculated as:

Diff (x)= Area (x)- Area (0), x=8 h; 12 h; 24 h; 48 h;

For each assay, the mean of the respective time point difference was calculated for the wells with NegCtr (Mean (Diff $(x)_{Negctr}$)). The fold change value over the NegCtr was calculated as follows:

FC over NegCtr (x) = Diff $(x)_{sample}$ / Mean (Diff $(x)_{Negctr}$)

Apoptosis assay with NRCMs

To test whether CDC/CF/CFSPh-derived EVs reduce apoptosis of stressed cardiomyocytes, neonatal rat cardiomyocytes (NRCMs) were plated at 4×10^4 cells per well of a 96-well plate in NRCM medium, consisting of 5% horse serum (Life Technologies™, Thermo Fisher Scientific), 100 units/mL penicillin g and 100 µg/mL streptomycin in DMEM/Ham´s F12. After two days, beating cells were visible in the cell layer. To mimic hypoxic conditions, the cells were incubated with 3 mM cobalt chloride solution (cobalt(II) chloride hexahydrate, Sigma-Aldrich, Merck Millipore, Merck KGaA, Darmstadt, Germany) in DMEM/Ham´s F12 for 1 h in a cell incubator (Safe 2020 Class II Biological Safety Cabinets, Thermo Fisher Scientific) at 37°C, 5% CO_2. $CoCl_2$ was washed off the cells twice with 1x D-PBS. Subsequently, NRCMs were incubated with either EVs isolated from CDCs, CFs or CFSPhs from different patients at a concentration of 3×10^9 particles per well in serum-free IMDM medium, serum-free IMDM medium (negative control) or NRCM medium (positive control). After 4 days, the cells were lysed with RNA lysis buffer (PeqGold). RNA isolation, reversed transcription and real-time PCR of the apoptosis markers *Fas*

and *Bax* were performed, as mentioned above. This protocol relied on previously published protocols with modifications [16, 28].

Statistics

Statistical analysis and graphs for each experiment were generated with the software SPSS Statistics 25 (IBM, Armonk NY). Data distribution was evaluated using box-whisker plots for each group. Values of three-fold interquartile range were seen as extreme outliers and removed. Descriptive data analysis was performed, and the Shapiro-Wilk test was used to check for normal distribution. Comparison of means was carried out using One-way ANOVA (analysis of variance). Groups were examined for homogeneity of variance by a Levene's test. The significance level α was set to $p < 0.05$. In case the Levene's test was not significant for a parameter, ANOVA was used to compare the means of the groups. Comparison of more than two groups was performed with the post-hoc test (a posteriori tests) Tukey-HSD (HSD = honestly significant difference) or Bonferroni correction. If the Levene's test was significant, robust tests such as the Welch and Brown-Forsythe tests for inhomogeneous variances were used. For multiple group comparison, the Games-Howell test and the Dunnett-T3 test were used as robust post-hoc tests. Comparison of two groups in Microsoft Excel 2010 was performed using the two-sided heteroscedastic Student's t-test. Bar diagrams show means +/- standard error of the mean (SEM). Significance levels are shown as: * for $p < 0.05$; ** for $p < 0.01$ and *** for $p < 0.001$.

5. Supplemental References

1. Abramoff MD (2004) Image Processing with ImageJ. Biophotonics Intl. 11:36-42
2. Afgan E, Baker D, Batut B, van den Beek M, Bouvier D, Cech M, Chilton J, Clements D, Coraor N, Gruning BA, Guerler A, Hillman-Jackson J, Hiltemann S, Jalili V, Rasche H, Soranzo N, Goecks J, Taylor J, Nekrutenko A, Blankenberg D (2018) The Galaxy platform for accessible, reproducible and collaborative biomedical analyses: 2018 update. Nucleic Acids Res 46:W537-W544 doi:10.1093/nar/gky379
3. Ashburner M, Ball CA, Blake JA, Botstein D, Butler H, Cherry JM, Davis AP, Dolinski K, Dwight SS, Eppig JT, Harris MA, Hill DP, Issel-Tarver L, Kasarskis A, Lewis S, Matese JC, Richardson JE, Ringwald M, Rubin GM, Sherlock G (2000) Gene ontology: tool for the unification of biology. The Gene Ontology Consortium. Nat Genet 25:25-29 doi:10.1038/75556
4. Becht E, McInnes L, Healy J, Dutertre C-A, Kwok IWH, Ng LG, Ginhoux F, Newell EW (2019) Dimensionality reduction for visualizing single-cell data using UMAP. Nature Biotechnology 37:38-44 doi:10.1038/nbt.4314
5. Bosch S, de Beaurepaire L, Allard M, Mosser M, Heichette C, Chretien D, Jegou D, Bach JM (2016) Trehalose prevents aggregation of exosomes and cryodamage. Sci Rep 6:36162 doi:10.1038/srep36162
6. Burridge PW, Holmstrom A, Wu JC (2015) Chemically Defined Culture and Cardiomyocyte Differentiation of Human Pluripotent Stem Cells. Curr Protoc Hum Genet 87:21 23 21-15 doi:10.1002/0471142905.hg2103s87

7. Butler A, Hoffman P, Smibert P, Papalexi E, Satija R (2018) Integrating single-cell transcriptomic data across different conditions, technologies, and species. Nature Biotechnology 36:411-420 doi:10.1038/nbt.4096
8. Cao J, Spielmann M, Qiu X, Huang X, Ibrahim DM, Hill AJ, Zhang F, Mundlos S, Christiansen L, Steemers FJ, Trapnell C, Shendure J (2019) The single-cell transcriptional landscape of mammalian organogenesis. Nature 566:496-502 doi:10.1038/s41586-019-0969-x
9. Carpentier G MM, Courty J and Cascone I. (2012) Angiogenesis Analyzer for ImageJ. In:4th ImageJ User and Developer Conference proceedings, Mondorf-les-Bains, Luxembourg, p 198-201
10. Doll S, Dressen M, Geyer PE, Itzhak DN, Braun C, Doppler SA, Meier F, Deutsch MA, Lahm H, Lange R, Krane M, Mann M (2017) Region and cell-type resolved quantitative proteomic map of the human heart. Nature communications 8:1469 doi:10.1038/s41467-017-01747-2
11. Gartner K, Battke C, Dunzkofer J, Huls C, von Neubeck B, Kellner MK, Fiestas E, Fackler S, Lang S, Zeidler R (2018) Tumor-derived extracellular vesicles activate primary monocytes. Cancer Med 7:2013-2020 doi:10.1002/cam4.1465
12. Hafemeister C, Satija R (2019) Normalization and variance stabilization of single-cell RNA-seq data using regularized negative binomial regression. Genome Biol 20:296 doi:10.1186/s13059-019-1874-1
13. Ibrahim AG, Cheng K, Marban E (2014) Exosomes as critical agents of cardiac regeneration triggered by cell therapy. Stem Cell Reports 2:606-619 doi:10.1016/j.stemcr.2014.04.006
14. La Manno G, Soldatov R, Zeisel A, Braun E, Hochgerner H, Petukhov V, Lidschreiber K, Kastriti ME, Lonnerberg P, Furlan A, Fan J, Borm LE, Liu Z, van Bruggen D, Guo J, He X, Barker R, Sundstrom E, Castelo-Branco G, Cramer P, Adameyko I, Linnarsson S, Kharchenko PV (2018) RNA velocity of single cells. Nature 560:494-498 doi:10.1038/s41586-018-0414-6
15. Lahm H, Jia M, Dressen M, Wirth F, Puluca N, Gilsbach R, Keavney BD, Cleuziou J, Beck N, Bondareva O, Dzilic E, Burri M, Konig KC, Ziegelmuller JA, Abou-Ajram C, Neb I, Zhang Z, Doppler SA, Mastantuono E, Lichtner P, Eckstein G, Horer J, Ewert P, Priest JR, Hein L, Lange R, Meitinger T, Cordell HJ, Muller-Myhsok B, Krane M (2021) Congenital heart disease risk loci identified by genome-wide association study in European patients. J Clin Invest 131 doi:10.1172/JCI141837
16. Lang JK, Young RF, Ashraf H, Canty JM, Jr. (2016) Inhibiting Extracellular Vesicle Release from Human Cardiosphere Derived Cells with Lentiviral Knockdown of nSMase2 Differentially Effects Proliferation and Apoptosis in Cardiomyocytes, Fibroblasts and Endothelial Cells In Vitro. PloS one 11:e0165926 doi:10.1371/journal.pone.0165926
17. Liang CC, Park AY, Guan JL (2007) In vitro scratch assay: a convenient and inexpensive method for analysis of cell migration in vitro. Nat Protoc 2:329-333 doi:10.1038/nprot.2007.30
18. Liao Y, Smyth GK, Shi W (2014) featureCounts: an efficient general purpose program for assigning sequence reads to genomic features. Bioinformatics 30:923-930 doi:10.1093/bioinformatics/btt656
19. Macosko EZ, Basu A, Satija R, Nemesh J, Shekhar K, Goldman M, Tirosh I, Bialas AR, Kamitaki N, Martersteck EM, Trombetta JJ, Weitz DA, Sanes JR, Shalek AK, Regev A, McCarroll SA (2015) Highly Parallel Genome-wide Expression Profiling of Individual Cells Using Nanoliter Droplets. Cell 161:1202-1214 doi:10.1016/j.cell.2015.05.002
20. Messina E, De Angelis L, Frati G, Morrone S, Chimenti S, Fiordaliso F, Salio M, Battaglia M, Latronico MV, Coletta M, Vivarelli E, Frati L, Cossu G, Giacomello A (2004) Isolation and expansion of adult cardiac stem cells from human and murine heart. Circ Res 95:911-921 doi:10.1161/01.RES.0000147315.71699.51
21. Mononen MM, Leung CY, Xu J, Chien KR (2020) Trajectory mapping of human embryonic stem cell cardiogenesis reveals lineage branch points and an ISL1 progenitor-derived cardiac fibroblast lineage. Stem Cells 38:1267-1278 doi:10.1002/stem.3236

22. Qiu X, Mao Q, Tang Y, Wang L, Chawla R, Pliner HA, Trapnell C (2017) Reversed graph embedding resolves complex single-cell trajectories. Nat Methods 14:979-982 doi:10.1038/nmeth.4402
23. Sassi Y, Avramopoulos P, Ramanujam D, Gruter L, Werfel S, Giosele S, Brunner AD, Esfandyari D, Papadopoulou AS, De Strooper B, Hubner N, Kumarswamy R, Thum T, Yin X, Mayr M, Laggerbauer B, Engelhardt S (2017) Cardiac myocyte miR-29 promotes pathological remodeling of the heart by activating Wnt signaling. Nature communications 8:1614 doi:10.1038/s41467-017-01737-4
24. Satija R, Farrell JA, Gennert D, Schier AF, Regev A (2015) Spatial reconstruction of single-cell gene expression data. Nature Biotechnology 33:495-502 doi:10.1038/nbt.3192
25. Subramanian A, Tamayo P, Mootha VK, Mukherjee S, Ebert BL, Gillette MA, Paulovich A, Pomeroy SL, Golub TR, Lander ES, Mesirov JP (2005) Gene set enrichment analysis: A knowledge-based approach for interpreting genome-wide expression profiles. Proceedings of the National Academy of Sciences 102:15545-15550 doi:10.1073/pnas.0506580102
26. Sun N, Yazawa M, Liu J, Han L, Sanchez-Freire V, Abilez OJ, Navarrete EG, Hu S, Wang L, Lee A, Pavlovic A, Lin S, Chen R, Hajjar RJ, Snyder MP, Dolmetsch RE, Butte MJ, Ashley EA, Longaker MT, Robbins RC, Wu JC (2012) Patient-specific induced pluripotent stem cells as a model for familial dilated cardiomyopathy. Science translational medicine 4:130ra147 doi:10.1126/scitranslmed.3003552
27. Trapnell C, Cacchiarelli D, Grimsby J, Pokharel P, Li S, Morse M, Lennon NJ, Livak KJ, Mikkelsen TS, Rinn JL (2014) The dynamics and regulators of cell fate decisions are revealed by pseudotemporal ordering of single cells. Nat Biotechnol 32:381-386 doi:10.1038/nbt.2859
28. Tseliou E, Fouad J, Reich H, Slipczuk L, de Couto G, Aminzadeh M, Middleton R, Valle J, Weixin L, Marban E (2015) Fibroblasts Rendered Antifibrotic, Antiapoptotic, and Angiogenic by Priming With Cardiosphere-Derived Extracellular Membrane Vesicles. J Am Coll Cardiol 66:599-611 doi:10.1016/j.jacc.2015.05.068
29. Wang L, Yu P, Zhou B, Song J, Li Z, Zhang M, Guo G, Wang Y, Chen X, Han L, Hu S (2020) Single-cell reconstruction of the adult human heart during heart failure and recovery reveals the cellular landscape underlying cardiac function. Nat Cell Biol 22:108-119 doi:10.1038/s41556-019-0446-7
30. Wu SM, Fujiwara Y, Cibulsky SM, Clapham DE, Lien CL, Schultheiss TM, Orkin SH (2006) Developmental origin of a bipotential myocardial and smooth muscle cell precursor in the mammalian heart. Cell 127:1137-1150 doi:10.1016/j.cell.2006.10.028
